# Evidence Base of Clinical Studies on Tai Chi: A Bibliometric Analysis

**DOI:** 10.1371/journal.pone.0120655

**Published:** 2015-03-16

**Authors:** Guo-Yan Yang, Li-Qiong Wang, Jun Ren, Yan Zhang, Meng-Ling Li, Yu-Ting Zhu, Jing Luo, Yan-Jun Cheng, Wen-Yuan Li, Peter M. Wayne, Jian-Ping Liu

**Affiliations:** 1 Centre for Evidence-based Chinese Medicine, Beijing University of Chinese Medicine, Beijing, China; 2 School of Basic Medical Sciences, Beijing University of Chinese Medicine, Beijing, China; 3 Xiyuan Hospital, China Academy of Chinese Medical Sciences, Beijing, China; 4 Guang’anmen Hospital, China Academy of Chinese Medical Sciences, Beijing, China; 5 Osher Center for Integrative Medicine, Brigham and Women’s Hospital and Harvard Medical School, Boston, Massachusetts, United States of America; Johns Hopkins Bloomberg School of Public Health, UNITED STATES

## Abstract

**Background:**

The safety and health benefits of Tai Chi mind-body exercise has been documented in a large number of clinical studies focused on specific diseases and health conditions. The objective of this systematic review is to more comprehensively summarize the evidence base of clinical studies of Tai Chi for healthcare.

**Methods and Findings:**

We searched for all types of clinical studies on Tai chi in PubMed, the Cochrane Library and four major Chinese electronic databases from their inception to July 2013. Data were analyzed using SPSS17.0 software. A total of 507 studies published between 1958 and 2013 were identified, including 43 (8.3%) systematic reviews of clinical studies, 255 (50.3%) randomized clinical trials, 90 (17.8%) non-randomized controlled clinical studies, 115 (22.7%) case series and 4 (0.8%) case reports. The top 10 diseases/conditions was hypertension, diabetes, osteoarthritis, osteoporosis or osteopenia, breast cancer, heart failure, chronic obstructive pulmonary disease, coronary heart disease, schizophrenia, and depression. Many healthy participants practiced Tai Chi for the purpose of health promotion or preservation. Yang style Tai Chi was the most popular, and Tai Chi was frequently practiced two to three 1-hour sessions per week for 12 weeks. Tai Chi was used alone in more than half of the studies (58.6%), while in other studies Tai Chi was applied in combination with other therapies including medications, health education and other physical therapies. The majority of studies (94.1%) reported positive effects of Tai Chi, 5.1% studies reported uncertain effects and 0.8% studies reported negative effects. No serious adverse events related to Tai Chi were reported.

**Conclusions:**

The quantity and evidence base of clinical studies on Tai Chi is substantial. However, there is a wide variation in Tai Chi intervention studied and the reporting of Tai Chi intervention needs to be improved. Further well-designed and reported studies are recommended to confirm the effects of Tai Chi for the frequently reported diseases/conditions.

## Introduction

Tai Chi, also known as Tai Chi Chuan/Quan or Taiji, originated in China, and was developed by a famous martial artist Wangting Chen in the end of Ming Dynasty (18^th^ century A.D) [1]. Tai Chi’s development has integrated the essence of Chinese folk and military martial arts, breathing and meditative techniques, and traditional Chinese medicine theory [2]. For centuries millions of Chinese have practiced Tai Chi’s flowing, meditative movements to cultivate and maintain health and well-being. In recent years, because of its health benefits, apparent safety and low cost, Tai Chi has gained popularity in both Eastern and Western countries as a promising low-intensity, mind-body exercise.

To date, an increasing number of clinical studies have documented the safety and health benefits of Tai Chi intervention. Several systematic reviews have examined the evidence available from randomized controlled trials or nonrandomized clinical trials on Tai Chi for a variety of specific diseases and health conditions, such as balance control and falls prevention [3–5], cardiovascular disease [6–8] and osteoarthritis [9], [10]. However, this peer-reviewed literature on Tai Chi is evolving rapidly, and no recent comprehensive systematic review has examined the overall evidence of the effectiveness of Tai Chi in prevention, treatment and rehabilitation for human health. Moreover, many reviews to date have been limited to English language publications thus excluding the majority of studies published in Asian languages.

We, therefore, conducted this study to systematically summarize all available clinical evidence on Tai Chi, in order to provide current state of evidence in support of the application of Tai Chi for healthcare, and to identify limitations and priorities for further clinical research.

## Materials and Methods

We systematically searched for the literature and used other systematic methods within the review.

### Data Sources and Searches

We searched for all clinical studies on Tai Chi in PubMed, the Cochrane Library, China National Knowledge Infrastructure (CNKI), Chinese Scientific Journal Database (VIP), Sino-Med, and Wanfang Database from their inception to 1^st^ July 2013. The search terms included “Taiji”, “Tai Ji”, “Tai-ji”, “Tai Chi”, “Tai Chi Chuan”, “Tai Chi Quan”, or “Taijiquan”. No language restriction was applied. As an example on one specific strategy, the search terms for PubMed database were as follows: (((((Taiji [Title/Abstract]) OR Tai Ji [Title/Abstract]) OR Tai Chi [Title/Abstract]) OR Tai Chi Quan [Title/Abstract]) OR Tai Chi Chuan [Title/Abstract]) OR Taijiquan [Title/Abstract] Filters: Humans.

### Study Selection

Two authors (GYY and LQW) screened the titles and abstracts of the hits, and full papers were retrieved and reviewed according to the inclusion criteria. The two authors (GYY and LQW) also classified all eligible clinical studies according to their study designs. If there was any uncertainty or discrepancy, a third author (JPL) was consulted.

### Inclusion/Exclusion Criteria

We included all types of clinical studies including systematic review (SR), randomized clinical trial (RCT), non-randomized controlled clinical studies (CCS) (quasi-randomized clinical trial or observational studies such as cohort or case-control study), case series (CS) and case report (CR) that included Tai Chi as the intervention for any disease/condition or healthy participants. Any type of Tai Chi, regardless of the style or training regimen was included. Studies applied Tai Chi in combination with Tai Chi pushing hands, Tai Chi sword, Tai Chi knife or other forms practiced with instruments were also included.

Anecdotes were excluded. Reports published in abstracts, and studies lacking basic information on Tai Chi interventions were excluded. Reviews irrelevant to Tai Chi intervention, or studies of complex intervention including Tai Chi as one of the intervention components which have no detailed description on Tai Chi intervention were also excluded.

### Data Extraction

Two authors (GYY and JPL) designed a structured data extraction form. The form consisted of the following sections:

(1) Publication information, including year of publication, study design, language, country, and funding information if available.
(2) Disease/condition. The names of diseases/conditions were extracted directly and then classified into different categories according to International Statistical Classification of Diseases and Related Health Problems 10^th^ Revision (ICD 10) [11]. Studies about disease prevention, health promotion or preservation were specially recorded.
(3) Tai Chi intervention. We extracted Tai Chi styles, the method of learning and practicing Tai Chi, the qualification of Tai Chi instructors. If Tai Chi was applied in combination with other therapies, other therapies were extracted.
(4) Outcomes and overall conclusions. We extracted all the outcomes directly and then classified them into different categories. If quality of life was reported, the measurement was also extracted if available. We also examined the overall authors’ conclusions (positive, negative or unclear). “Positive” was made if the study achieved its objective, and statistically favored Tai Chi; “negative” was made if the study did not achieve its objective, or did not favor Tai Chi; “unclear” was made if the study objective was unclear or the conclusions were inconclusive.

Nine authors (GYY, LQW, JR, YZ, MLL, YTZ, JL, YJC, and WYL) participated in data extraction, and prior to that, all of them were trained twice in order to fully understand the standard and skills of data extraction of this study. All extractions were verified by one author (GYY). Any discrepancies were discussed with the other authors for consensus.

### Data Analysis

We performed data analysis using SPSS17.0. Data were presented by counts, percentage and frequency.

## Results

### Selection of Studies

**Fig 1:**
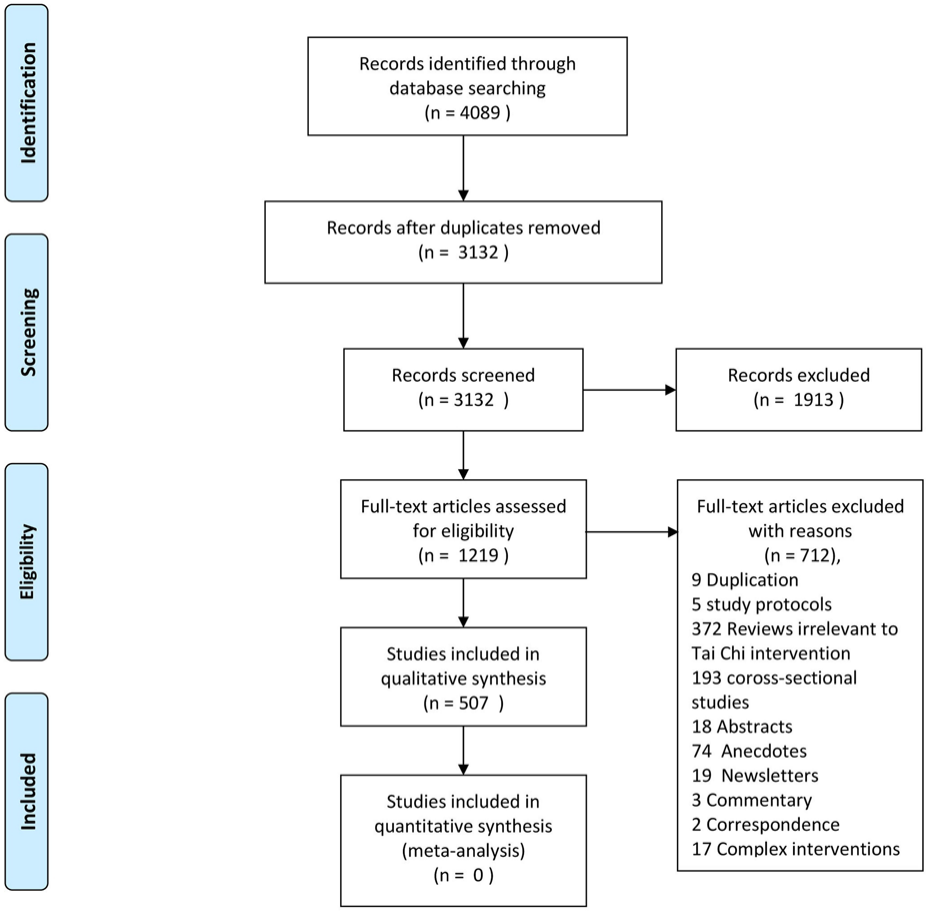
Flow diagram. Presentation of the procedure of study searching and selection with numbers of articles at each stage.

Fig. 1 shows the flow chart of study searching and selection. We identified 4089 references, and after screening according to the inclusion criteria, 3582 reports were excluded with reasons. Finally, 507 studies were included in this overview.

### General Characteristics of Included Studies

Among the 507 studies, 265 (52.3%) were published in Chinese, 236 (46.5%) in English, and 6 (1.2%) in Korean. The clinical studies on Tai Chi cover almost all levels of intervention study, including 43 (8.5%) systematic reviews of clinical studies, 255 (50.3%) randomized clinical trials, 90 (17.8%) non-randomized controlled clinical studies, 115 (22.7%) case series and 4 (0.8%) case reports.

The 507 studies were conducted in 21 different countries, and the majority of studies (317/507, 62.5%) were conducted in China, followed by the United States (104/507, 20.5%) (Table 1). 230 (45.4%) studies reported funding information, among which 228 (99.1%) studies were funded by government and two studies were funded by family foundation.

**Table 1:** Number of clinical studies on Tai Chi conducted in different countries (n = 507).

Country	Study design (number of studies)					Total (%)
	SR	RCT	CCS	CS	CR	
China	10	148	72	86	1	**317 (62.5)**
USA	9	70	4	18	3	**104 (20.5)**
South Korea	6	4	11	1	0	**22 (4.3)**
Australia	2	11	2	1	0	**16 (3.2)**
UK	8	4	1	0	0	**13 (2.6)**
Canada	2	2	0	2	0	**6 (1.2)**
Netherlands	2	2	0	1	0	**5 (1.0)**
France	0	4	0	1	0	**5 (1.0)**
Poland	1	2	0	0	0	**3 (0.6)**
Germany	0	1	0	1	0	**2 (0.4)**
Israel	1	1	0	0	0	**2 (0.4)**
Japan	0	1	0	1	0	**2 (0.4)**
New Zealand	0	2	0	0	0	**2 (0.4)**
Spain	0	0	0	2	0	**2 (0.4)**
Finland	1	0	0	0	0	**1 (0.2)**
Iran	0	1	0	0	0	**1 (0.2)**
Italy	0	1	0	0	0	**1 (0.2)**
Norway	0	0	0	1	0	**1 (0.2)**
Singapore	1	0	0	0	0	**1 (0.2)**
Switzerland	0	1	0	0	0	**1 (0.2)**

Abbreviations: SR, systematic review; RCT, randomized clinical trial; CCS, non-randomized controlled clinical studies (quasi-randomized clinical trial or observational studies such as cohort or case-control studies); CS, case series; CR, case report; USA, United States of America; UK, United Kingdom.

**Fig 2:**
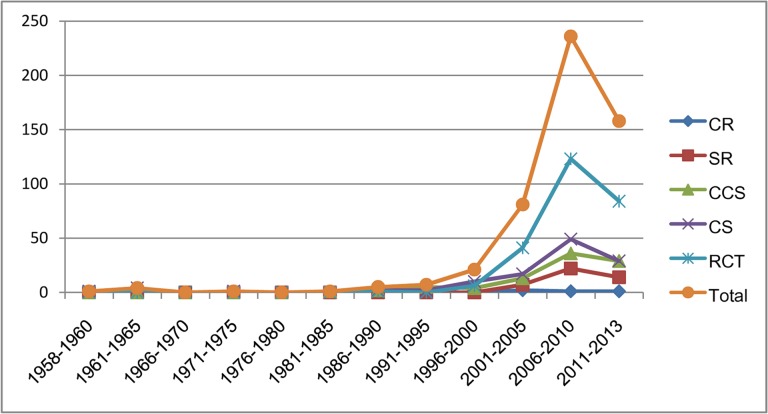
Study designs over time in the numbers of published clinical studies on Tai Chi. Abbreviation: SR, systematic review; RCT, randomized clinical trial; CCS, non-randomized controlled clinical studies (quasi-randomized clinical trial or observational studies such as cohort or case-control studies); CS, case series; CR, case report.

The first clinical study on Tai Chi published in 1958 in Chinese, which was a case series on Tuberculosis (TB) [12], and the first RCT on Tai Chi was published in 1988 in Chinese, in which Tai Chi combined with Qigong were designed as a cardiac rehabilitation program [13]. The publication of clinical studies on Tai Chi increased with years, especially after the year 2000 (Fig. 2).

### Disease/Condition Categories

192 (37.9%) of the 507 studies enrolled only healthy participants to test the effect of Tai Chi on health promotion or preservation (142/507, 28.0%), and balance control or falls prevention (50/507, 9.9%).

The remaining clinical studies addressed a total of 93 diseases/conditions. Among them, the majority were diseases/conditions of the circulatory system and the musculoskeletal system and connective tissue, based on ICD 10 categorization (Table 2). The top 10 diseases/conditions included hypertension, diabetes, osteoarthritis, osteoporosis or osteopenia, breast cancer, heart failure, chronic obstructive pulmonary disease (COPD), coronary heart disease, schizophrenia, and depression (Table 3).

**Table 2:** Clinical trials of Tai Chi organized by prevalence of disease categories based on ICD-10 classifications.

Chapter	Blocks	Disease/conditions(ICD-10 codes)	No. of study*
IX	I00–I99	I00–I99 Diseases of the circulatory system	99
XIII	M00–M99	M00–M99 Diseases of the musculoskeletal system and connective tissue	86
IV	E00–E90	E00–E90 Endocrine, nutritional and metabolic diseases	36
V	F00–F99	F00–F99 Mental and behavioural disorders	31
II	C00–D48	C00–D48 Neoplasms	21
VI	G00–G99	G00–G99 Diseases of the nervous system	18
I	A00–B99	A00–B99 Certain infectious and parasitic diseases	10
XIV	N00–N99	N00–N99 Diseases of the genitourinary system	9
XI	K00–K93	K00–K93 Diseases of the digestive system	7
XIX	S00–T98	S00–T98 Injury, poisoning and certain other consequences of external causes	7
III	D50–D89	D50–D89 Diseases of the blood and blood-forming organs and certain disorders involving the immune mechanism	3
XII	L00–L99	L00–L99 Diseases of the skin and subcutaneous tissue	3
VIII	H60–H95	H60–H95 Diseases of the ear and mastoid process	2
X	J00–J99	J00–J99 Diseases of the respiratory system	1
XVII	Q00–Q99	Q00–Q99 Congenital malformations, deformations and chromosomal abnormalities	1

Abbreviations: ICD-10, International Classification of Diseases, Tenth Revision.

* Some systematic reviews involved more than one type of diseases or conditions.

**Table 3:** Top 10 diseases/conditions included in clinical studies on Tai Chi.

Disease/condition	Study design(number of studies)					Total (%)
	SR*	RCT	CCS	CS	CR	
Hypertension	6	10	7	9	0	32 (6.3)
Diabetes	2	14	5	7	0	28(5.5)
Osteoarthritis	10	8	2	2	0	22(4.3)
Osteoporosis or osteopenia	2	14	0	1	0	17(3.4)
Breast cancer	5	9	1	0	0	15(3.0)
Heart Failure	0	9	5	1	0	15(3.0)
COPD	1	8	4	1	0	14(2.8)
Coronary heart disease	3	4	4	1	0	12(2.4)
Schizophrenia	1	7	2	0	0	10(2.0)
Depression	3	4	1	0	0	8(1.6)

Abbreviations: SR, systematic review; RCT, randomized clinical trial; CCS, non-randomized controlled clinical studies (quasi-randomized clinical trial or observational studies such as cohort or case-control studies); CS, case series; CR, case report; USA, United States of America; UK, United Kingdom; COPD, chronic obstructive pulmonary disease.

* Some systematic reviews involved more than one type of diseases or conditions.

### Tai Chi Intervention

To avoid duplication of information, we here only analyzed the Tai Chi intervention in the 464 clinical studies including randomized clinical trials, non-randomized controlled clinical studies, case series and case reports, and excluded information in systematic reviews.

Table 4 shows the Tai Chi styles applied in the 464 clinical studies. Among the popular Tai Chi styles, namely Chen, Yang, Wu, Wu/Hao, Sun, the most commonly applied Tai Chi style was Yang style, among which the 24-form Simplified Yang style Tai Chi was the most popular one. Some studies applied more than one type of Tai Chi intervention.

**Table 4:** Tai Chi styles applied in 464 clinical studies including RCT, CCS, CS and CR.

Tai Chi style	No. of study	Frequency *
**Yang style**	**338**	**72.84%**
*24-form Simplified Yang style*	200	43.10%
*37-form Yang style*	10	2.16%
*42-form Yang style*	28	6.03%
*48-form Yang style*	17	3.66%
*85-form Yang style*	3	0.65%
*108-form Yang style*	8	1.72%
*Modified Yang style*	61	13.15%
*Unspecified forms of Yang style*	11	2.37%
**Chen style**	**18**	**3.88%**
*32-form Chen style*	1	0.22%
*42-form Chen style*	2	0.43%
*83-form Chen style*	1	0.22%
*Modified Chen style*	3	0.65%
*Unspecified forms of Chen style*	11	2.37%
**Sun style**	**21**	**4.53%**
Modified Sun style**	18	3.88%
*Unspecified forms of Sun style*	3	0.65%
**Wu style**	**6**	**1.29%**
*Modified Wu style*	2	0.43%
*Unspecified forms of Wu style*	4	0.86%
**Others**	**110**	**23.71%**
Modified Sun and Yang style**	8	1.72%
*Unspecified style*	102	21.98%

Presentation of Tai Chi interventions in the 464 clinical studies including randomized controlled trials, clinical controlled trials, case series and case reports.

Abbreviation: RCT, randomized clinical trial; CCS, non-randomized controlled clinical studies (quasi-randomized clinical trial or observational studies such as cohort or case-control studies); CS, case series; CR, case report.

*Some studies applied more than one type of Tai Chi.

**Most of the modifications were developed by Dr. Paul Lam and colleagues for arthritis, diabetes, etc.

In the 464 clinical studies, Tai Chi practice varied from 10 minutes to 2 hours each session, 2 to 14 sessions per week, and three 1-hour sessions per week (61/464, 13.1%) and two 1-hour sessions per week (58/464, 12.5%) were most popular. The duration of Tai Chi intervention varied from five days [14] to three years [15], [16], and the most common duration was 12 weeks (122/464, 26.3%), followed by 24 weeks (83/464, 17.9%). 47(10.1%) studies reported follow-up information, and the follow-up duration varied from four weeks (5/464, 1.1%) to six years [17].

Three hundred and twenty-three studies reported the methods of learning Tai Chi. In the majority of studies participants learned Tai Chi under the guidance of Tai Chi instructors (319/464, 68.8%). Two studies (2/464, 0.4%) reported participants learned Tai Chi by themselves with videotape/DVD, and two studies (2/464, 0.4%) enrolled participants who were already Tai Chi practitioner.

Out of the 319 studies which participants learned Tai Chi from instructors, 227 studies (227/319, 71.2%) described the qualification of Tai Chi instructors. Among them, 42 studies reported instructors’ specific years of Tai Chi teaching experience, which ranged from 4 years to 31 years with an average of 13.4 years. However, the majority of studies described the qualification of instructors with simple words including ‘professional’, ‘certified’, ‘qualified’, ‘trained’, ‘registered’, ‘experienced’, ‘expert’, ‘master’, ‘coach’ and ‘exercise therapist’.

Participants practiced Tai Chi by themselves at home with support of videos and/or paper guidance such as guide books (18/464, 3.9%), only practice Tai Chi during class under the guidance and supervision of instructors (250/464, 53.9%), or both under the guidance of instructors during class and by themselves at home with support of video and/or paper guidance (59/464, 12.7%). Six studies stated participants were organized to practice Tai Chi together, but it was not clear whether training took place with the supervision of instructors. The remaining 131 studies not reported how participants practiced Tai Chi.

In more than half of studies Tai Chi intervention was used alone (272/464, 58.6%), while in the remaining studies Tai Chi was applied in combination with other therapies including conventional medications, herbal medications, acupuncture, diet and lifestyle guidance, health education, psychological therapy, yoga, qigong, and other physical therapies.

### Outcomes and Main Findings

Among the 507 studies, the most frequently reported outcomes were physical performance, symptoms and psychological well-being. 46 studies (46/507, 9.1%) reported incidence of health-related events including falls, fracture, angina and others; 110 studies (110/507, 21.7%) reported quality of life, including SF-36, SF-12, and disease-specific questionnaires such as Minnesota Living With Heart Failure Questionnaire and St George's Respiratory Questionnaire; 163 studies (163/507, 32.1%) reported symptoms; 369 studies (369/507, 72.8%) reported physical performance including strength, flexibility, cardiovascular function, balance, pulmonary function, body mass index, biomarker, etc.; 139 studies (139/507, 27.4%) reported psychological outcomes including depression, stress, mood, fear of falling, self-efficacy, anxiety, self-esteem, quality of sleep, etc.; 10 studies (10/507, 2.0%) reported satisfaction; 42 studies (42/507, 8.3%) reported compliance; and 105 studies (105/507, 20.7%) reported safety.

Out of the 105 studies, 65 studies (65/507, 12.8%) stated that there were no adverse events during the overall course of intervention, and 40 studies (40/507, 7.9%) reported adverse events that were related to other therapies such as conventional medications. No serious adverse events were reported.

The majority of studies (477/507, 94.1%) found positive effect of Tai Chi, 26 (5.1%) studies found uncertain effect of Tai Chi including 20 systematic reviews, and four (0.8%) studies found negative effect of Tai Chi which were all RCTs, assessing Tai Chi for health promotion, type 2 diabetes and fall prevention. Systematic reviews which found uncertain results assessed various diseases including osteoarthritis, rheumatic arthritis, breast cancer, Parkinson disease, coronary heart disease, hypertension, and type 2 diabetes. Four systematic reviews assessed the effect of Tai Chi on psychological well-being in patients with different diseases and four systematic reviews focused on fall prevention of Tai Chi for the elderly.

Out of the 460 studies including RCT, CCS and CS, 189 (41.1%) studies reported information on dropout or withdrawal, among which 20 studies stated there was no withdrawal or dropout during interventions. The main reasons for dropout or withdrawal varied, and included loss of interest in the Tai Chi program, poor attendance, time conflict, moving out of the area, medical illness unrelated to Tai Chi, travel abroad, hospitalized or bedridden, death and refusal of follow-up assessment.

## Discussion

This systematic review reflects the most comprehensive analysis to date of the clinical evidence on Tai Chi for rehabilitation and health, including narrative syntheses of systematic reviews, randomized controlled trials, non-randomized clinical controlled trials, case series and case reports, and with no language exclusions.

Our findings show that there is a steady growth of clinical evidence for Tai Chi, especially higher level evidence such as RCTs. The number of publication increased from 1991 with the peak during 2006 to 2010. One possible reason for the recent decline in publication rate is publication lags in different databases for the year 2011, 2012 and 2013. In addition, we searched all the available databases until 1^st^ July 2013, thus an update searching should be conducted in the future to confirm whether there are more publications identified in the more recent time.

A wide range of diseases/conditions such as hypertension, diabetes, osteoarthritis, osteoporosis, breast cancer, heart failure, COPD, coronary heart disease, schizophrenia, and depression were addressed in clinical studies on Tai Chi. Most diseases were in the circulatory system, the musculoskeletal system and connective tissue. Possible reasons that these conditions were most studied include the physiological and biomechanical processes Tai Chi training influences. With respect to cardiorespiratory conditions, it has been proposed that Tai Chi might have benefits through its moderate aerobic activity, and enhanced respiratory efficiency [18], [19]. With respect to musculoskeletal conditions, evidence supports positive effects on lower extremity strength and flexibility, proprioception, and neuromuscular and cognitive-motor coordination [20–23]. Almost all included studies in this review demonstrated important health benefits of Tai Chi in prevention, treatment or rehabilitation.

Tai Chi has been examined in clinical trials in 21 countries, including both developed and developing countries worldwide. Of note, this research is mainly supported by governments, indicating the broad higher level interest in Tai Chi among national health care organizations.

Adequate intervention details reported in a clinical trial are important for other researchers to make a clinical decision and replicate the treatments. In this review, the majority of included clinical trials provided inadequate description of Tai Chi intervention, such as the reporting of Tai Chi style and form, session, frequency, duration, learning methods and qualification of instructors. We recommend that the design, conduct and reporting of Tai Chi intervention should be standardized. One systematic review [24] assessed the adequate reporting of Tai Chi intervention in the existing English randomized clinical trials by a 10-item mini-checklist and CONSORT statement, and the findings also underscored the necessity of developing a guideline for further investigators to report the Tai Chi intervention in clinical trials.

There are different styles of Tai Chi practiced in modern society and these can be broadly classified into traditional styles (e.g. Chen, Yang, Wu/Hao, Wu, and Sun) and competition forms according to the General Administration of Sport of China. All the different styles of Tai Chi are developed from traditional Chen style Tai Chi. In this review, we found that the most frequently applied Tai Chi style was Yang style, especially the 24-form Simplified Yang style Tai Chi. As we can see from the history of Tai Chi development, Yang style Tai Chi became the most popular Tai Chi due to various reasons by the end of the 19^th^ century. Another possible reason for its popularity is that the Chinese government has promoted its generalization since the establishment of People’s Republic of China. To make Tai Chi easier to learn, practice and remember, the General Administration of Sport of China issued a book titled ‘*Simplified Tai Chi*’ in 1956 according to traditional Yang style Tai Chi, which deleted difficult and duplicate forms and simplified the 81-form Yang style Tai Chi into 24 forms [25]. Therefore, the high frequency is not necessarily due to its better effects on health. More studies are warranted in the future to understand the effects of different styles of Tai Chi intervention.

It is still unclear about the potential advantages and disadvantages of different Tai Chi styles and forms for people with different health status. In this review, clinical studies applied different styles of Tai Chi, and within a style different forms were used. For example, studies that applied the Yang style variously employed 108, 37, 24, and 9 movement forms, or they extracted subsets of movements from these forms to be practiced in novel sequences or repetitively as single movement phrases. Some included studies did not specify the Tai Chi styles practiced.

For Tai Chi instructors, few studies provide any detailed information on the experience or qualifications of Tai Chi instructors. Since Tai Chi interventions can include many qualitative components, including imagination, philosophy, encouragement, and even the apparent embodiment of Tai Chi principles, it is likely that individual qualifications and personalities play a key role in the success or failure of Tai Chi intervention. In addition, in the majority of clinical studies, participants practiced Tai Chi supervised by only one instructor, so the effects of the instructors cannot be separated from the effects of Tai Chi intervention.

For Tai Chi intensity and duration, the studies included in this review exhibit a wide range of variability with respect to Tai Chi training formats, frequencies, and durations. The majority of participants practiced 1 hour Tai Chi (session), 2 to 3 times per week (frequency), for 12 weeks (duration). Exercise intensity may vary greatly depending on the fitness of the individual, the style, depth of stances, the vigor of performing the movement, etc. There are still no conclusive recommendations for Tai Chi intensity and duration for people with different health status.

The adherence or compliance to intervention has direct influence on the evaluation of interventions. As for Tai Chi, patients’ participation plays a critical role in assessing the effects. In this review, less than half of included studies reported the information of dropout or withdrawal. The most common reasons were loss of interest, and time conflict. Further research should consider these factors when designing and analyzing results from clinical studies.

The majority of the included clinical studies (94.1%) found positive results of Tai Chi. Among the 43 systematic reviews on Tai Chi, 20 found the potential beneficial effects of Tai Chi, however, due to methodological flaws or small sample sizes of trials, they could not draw confirm conclusions based on current evidence. These systematic reviews assessed various diseases including osteoarthritis, rheumatic arthritis, breast cancer, Parkinson disease, coronary heart disease, hypertension, and type 2 diabetes; they also focused on fall prevention of Tai Chi for the elderly. However, conclusions across systematic reviews are inconsistent. Further studies are still needed to confirm the effects of Tai Chi for these diseases.

No intervention-related serious adverse events were reported in included studies. A review systematically assessed the frequency and quality of adverse events reports in RCTs of Tai Chi that were published in English [26]. It concluded that ‘Tai Chi was unlikely to result in serious adverse events, but it might be associated with minor musculoskeletal aches and pains. ‘ Similarly, this review was also unable to draw conclusions regarding the safety of Tai Chi due to poor and inconsistent reporting of adverse events.

There are some limitations in our review. Firstly, we did not include cross-sectional studies that also contribute a large amount of evidence on Tai Chi, since we aimed at summarizing clinical evidence on Tai Chi as an intervention. Publication bias was also a limitation in this review. Although we did not limit our search by language, we only found articles published in English, Chinese and Korean. Thus, it is possible that we missed many trials published in other languages. Thirdly, we were able to describe trends and characteristics of clinical studies on Tai Chi as a whole; however, we failed to focus on methodological quality of included studies and specific diseases/conditions to provide detailed information on the effects of Tai Chi.

We have some recommendations for further clinical studies on Tai Chi. Firstly the reporting of Tai Chi interventions in clinical trials should be improved. In this review, we found the majority of included clinical trials inadequately reported Tai Chi intervention. Based on CONSORT and Revised Standards for Reporting Interventions in Clinical Trials of Acupuncture (STRICTA) [27], the following items are highly recommended for reporting Tai Chi intervention in clinical trials: Tai Chi style and forms, session, frequency, duration, the rational using a particular Tai Chi style or protocol, learning method, practicing method, instructor qualification or experience, assessment of the proficiency of performance, compliance or adherence, and follow-up. Secondly, the effects of instructor qualification should be addressed in further studies on Tai Chi, and guidelines are needed for better selecting and reporting of the characteristics of instructors, including experience and personality. Thirdly, since few studies examined the relationship between the intensity and/or duration and the effects of Tai Chi, further studies are needed to optimize effective evidence-based dose-response effects of Tai Chi. Fourthly, we recommend future studies set methods to encourage the compliance to intervention in Tai Chi clinical trials, and consider the compliance when calculating sample size during study design. And finally, patient-centered outcomes such as quality of life should be paid more attention to in further studies on Tai Chi.

## Conclusions

In conclusion, the existing clinical studies on various diseases/conditions suggest that the quantity and evidence base of clinical studies on Tai Chi is substantial. However, there is a wide variation of Tai Chi intervention in style, intensity, duration and learning and practicing methods. The reporting of Tai Chi intervention needs to be improved. Though the majority of studies report positive health-related effects of Tai Chi, further studies with better reporting are needed to confirm the effects of Tai Chi for frequently reported diseases/conditions.

## Supporting Information

S1 Text PRISMA Checklist. (DOC)

S1 Table Characteristics of included clinical studies on Tai Chi. (DOCX)
