# Supplementary material for: Evidence Base of Clinical Studies on Tai Chi: A Bibliometric Analysis
Source: PLoS One. 2015 Mar 16;10(3):e0120655. doi: 10.1371/journal.pone.0120655 (PMC4361587; doi:10.1371/journal.pone.0120655)
Supplement: S1 Table — (DOCX) [file pone.0120655.s002.docx]

**Table S1.** Characteristics of included clinical studies on Tai Chi.

| **Study ID** | **Study design** | **Country** | **Disease/conditions** | **Tai Chi Intervention** | **Outcomes** |
| --- | --- | --- | --- | --- | --- |
| Han YB 2012 | CCS | China | Metabolic syndrome | 24-form simplified Tai Chi, 60 minutes per time, 3 times per week, for 12 weeks. | Physical performance: BMI |
| Wang YG 2007 | CCS | China | Type 2 diabetes | 24-form simplified Tai Chi, practiced under the instruction of a coach for one week before the beginning of study to grasp the skills and key points．60 minutes per time, once per week, for 12 weeks. Adjusted the intensity according to patients' health status. | Physical performance; |
| Shen YW 2011 | CCS | China | Type 2 diabetes | Tai Chi, 30 minutes per time, with 10 minutes of warm-up and 5 minutes of cooling down,3 or 6 times per week, for 12 weeks. | Symptom; |
| Xie W 2012 | CCS | China | Health promotion | Tai Chi was practiced in three periods-training (2 months), reinforcement (2 months) and improvement (2 months). In training period, the coach taught the movements; in enforcement period, the coach corrected the movements if necessary; in the improvement period, the coach evaluated patients' movements and skills. | Aging; |
| Chang MY2012 | CCS | China | Health promotion | Cheng 37 standardized Tai Chi, practiced under the instruction of a teacher with 10 years of Tai Chi teaching experience, 60 minutes per session, three times per week, for 12 weeks. | Physical performance: cardiovascular function, BMI; Psychological: anxiety; |
| Lan C2008 | CCS | China | Health promotion | Each session included 20 min of warm up, 24 min of TCC practice and 10 min of cool down. Warm up exercises comprised of 10 movements (range of motion exercises, stretching and balance training) with 10 to 20 repetitions. Each TCC set included 108 postures, with some repeated sequences. | Physical performance: flexibility, cardiovascular function, pulmonary function, BMI; |
| Lin MR2006 | CCS | China | Falls Prevention | Chen-style tai chi with 13 movements, practiced under the instruction of an instructor and 5 assistants who volunteered from a local association for Chen-style tai chi in Taichung County. 1 hour per day in the morning at 5:30 to 6:30am, 6 days per week, and each 1-hour session consisted of a 10-minute warm-up, 45 minutes of tai chi practice, and a 5-minute cool-down. | Incidence: falls; Physical performance: balance; Psychological: fear of falling; |
| Lee HY2006 | CCS | South Korea | Knee Osteoarthritis | Tai Chi: 8 basic movements and 6 complex movements. Twice per week, 60 minutes per session, consisting 10 minutes of warm-up, 30 minutes of TCC practice and 10 minutes of cool down, for 8 weeks. | Symptom; |
| Nnodim JO2006 | CCS | USA | Balance control | 24-form simplified Yang style Tai Chi, three 1-hour sessions per week, for 10 weeks. | Physical performance: balance; |
| Chen YS2011 | CCS | Australia | Balance control | 24-form simplified Yang style Tai Chi, 3 times a week for 12 weeks. Each session consisted of 20 minutes of warm-up exercises and 40 minutes of Tai Chi practice. Warm-up exercises consisted of 22 modified Tai Chi activities. The first 6 weeks was a learning period. Participants were taught the 13-form Yang style Tai Chi. In the next 6 weeks, participants repeatedly practiced the sequence at a slow pace, and their performance was closely monitored by the instructor. | Physical performance: perception, balance; |
| Hung JW2009 | CCS | China | Type 2 diabetes | Cheng’s TCC, 3 sessions a week, 60 min a session, for 12 weeks. Each session included a 10-min warm-up (including stretching and balancing exercises), 40 min TCC exercise, and a 10-min cool-down, from 8.30 am to 9.30 am. | Physical performance: perception; |
| N. Tsang WW2004 | CCS | China | Balance control | Subjects in the Tai Chi group undertook supervised Tai Chi training in the Ng style for 1.5 h in the morning, 6 times per week for 8 weeks. The first 10 min were allocated for warm-up, with the rest of the time for Tai Chi practice. | Physical performance: perception, balance; |
| Lee LYK2007 | CCS | China | Health promotion | Tai Chi, three times a week, 60 minutes each session, for 26 weeks. | QOL:HRQOL; Psychological: self-esteem; |
| Chen YS2012 | CCS | Australia | Health promotion | 24-form simplified Yang style Tai Chi, 60 minutes each session, 3 times per week, for 12 weeks. Each session consisted of 22 warm-up exercises modified from TC activities that lasted 20 min and 40 min of TC practice. | Physical performance: strength, perception; |
| Li YH2007 | CCS | USA | Balance control | 24-form simplified Yang style Tai Chi, one 1-hour session per week for 48 weeks. Each session began with stretching, breathing and stepping exercises for about 30 min, and the remaining time was TC practice. | Physical performance: strength, flexibility, balance; |
| Choi HJ2005 | CCS | South Korea | Falls Prevention | Sun-style Tai Chi, 35 minutes per session, 3 sessions per week, for 12 weeks. Each session consisted of 10 minutes of warming-up exercise, 20 minutes of 12 Tai Chi movements, and 5 minutes of cooling-down exercise. | Incidence: falls; Physical performance: strength, flexibility, balance; |
| Lee HY2008 | CCS | South Korea | Knee Osteoarthritis | Sun-style 24 forms of Tai Chi, supervised by qualified instructor, twice per week, 60 minutes per session, for 12 weeks. Each session consisted of 10 minutes of warn up, 30 TC practice, and 10 minutes of cool down. | Symptom; Physical performance: flexibility, balance; Psychological: fear of falling; |
| Ahn S2012 | CCS | South Korea | Type 2 diabetes | 21 movements from the combined Yang and Sun style of Tai Chi, completed a supervised 1-hour session twice weekly for 12 weeks. | QOL:SF-36;Symptom; Physical performance: balance; |
| Liu MR2008 | CCS | South Korea | Health promotion | Tai Chi, twice per week, led by a instructor with 15 years of teaching experience, 60 minutes per session, for 12 weeks. Each session consisted of 10 minutes of warm up, 40 minutes of TC and 10 minutes of cool down. | Physical performance: cardiovascular function; |
| Song R2009 | CCS | South Korea | Health promotion | Sun and yang-Tai chi, 3 sessions per week, 60 minutes per session, for 24 weeks. Each session consisted 5 minutes of warm up, 40-50 minutes of TC, and 5-10 minutes of cool down. | QOL: SF-36; Psychological performance: cardiovascular function; |
| Lee LYK2012 | CCS | China | Health promotion | Chen-style Tai Chi, 80-min per session, practiced under instruction of a instructor with more than 10 years of teaching experience, once per week, for 10 weeks. Each Tai Chi session started with a 15-min warm-up phase, followed by a 60-min activity phase and ended with a 5-min cool-down phase. | Psychological: stress; |
| Thornton EW2004 | CCS | UK | Health promotion | 24-form simplified Yang style Tai Chi, practiced under instruction of a certified Tai Chi instructor from the Hong Kong Tai Chi Association, 3 times per week, 60 minutes each session, for 12 weeks. | Physical performance: cardiovascular function, balance; |
| Park IS2010 | CCS | South Korea | Coronary artery disease | Combined form of the Yang and Sun styles, once a week for 60 minutes, in combination with a requirement for home exercise for 30 minutes at least three times a week to meet the recommended frequency of exercise, for 24 weeks. | QOL:SF-36; Physical performance: cardiovascular function; |
| Cheung SY2007 | CCS | China | Lower-limb disabilities | Wu-style TCC, 2 sessions per week, for 15 weeks. Each session lasted 60 minutes, which included 5–10 minutes warm-up, about 45 minutes of Tai Chi learning and practice, and 5–10 minutes of cool-down exercises. | Physical performance: flexibility, cardiovascular function, pulmonary function; |
| Yeh SH2009 | CCS | China | Type 2 diabetes | Cheng’s Tai Chi 37 forms, 60 min per session, 3 sessions per week, administered by a Tai Chi instructor with 31 years of teaching experience at a community cultural centre. Each session included a 10 min warm-up, a 40 min practice and a10 min cool-down. | Physical performance; biomarker; |
| Lee LYK2009 | CCS | China | Health promotion | Chen-style Tai Chi, three times a week, 60 minutes per session, for 26 weeks. | QOL:SF-12; Physical performance: flexibility, balance; Satisfaction; |
| Lee HY2012 | CCS | South Korea | Rheumatoid arthritis | Sun Style Tai Chi, practiced under instruction of a certificated Tai-Chi tutor instructor for arthritis in Australia and Korea, 1 hour per session, twice a week for 12 weeks. | QOL: Arthritis Impact Measurement Scale2;Symptom; Physical performance: strength, flexibility, balance; Psychological: self-efficacy; Satisfaction; |
| Lee EN2007 | CCS | South Korea | Ankylosing spondylitis | 21 movements based on Sun-style tai chi developed by Dr Paul Lam et al, practiced under instruction of two qualified tutors with 4 years of teaching experience. 60 minutes per session, 2 sessions per week for 8 weeks. | Symptom; Physical performance: flexibility; Psychological: depression; |
| Yan JH1999 | CCS | USA | Health promotion | 24-form simplified Yang style Tai Chi, 3 times per week, 45 minutes per session, while under the supervision of trained personnel, for 8 weeks. | Physical performance: strength; |
| Evangelos A2003 | CCS | USA | Health promotion | 12 forms of Chen-style TC, 60 minutes per session, 3 sessions per week, for 20 weeks. | Physical performance: strength, flexibility; |
| Lee KY2006 | CCS | South Korea | Rheumatoid arthritis | Sun-style TC, once per week, 50 minutes per session, for 12 weeks. Each session consisted of 15 minutes of warm up, 20 minutes of practice and 15 minutes of cool down. | Symptom; Physical performance: ADL, balance; |
| LAN C1999 | CCS | China | Coronary artery disease | Classical Yang TCC (108 forms), 55 minutes per session, 3 sessions per week, for 48 weeks. | Physical performance: cardiovascular function; |
| Lee EN2004 | CCS | South Korea | Essential Hypertension | Sun-style TC (12 movements), 2 sessions per week, 60 minutes per session, for 6 weeks. | Physical performance: cardiovascular function; |
| Lee LYK2010 | CCS | China | Health promotion | Tai Chi, practiced under instruction of a instructor with more than 10 years of teaching experience, one hour per session, 3 sessions per week, for 26 weeks. Each session started with a 15-minute warm-up phase, followed by a 40-minute activity phase and ending with a five-minute cool down phase. | QOL:SF-12; Psychological: self-esteem, social support; Satisfaction; Compliance; |
| Cui YQ 2010 | CCS | China | Health promotion | 24-form simplified Yang style Tai Chi, 45 minutes per session, 3 sessions per week, for 8 weeks. | Physical performance: BMI; |
| Jing JH 2012 | CCS | China | Health promotion | 24-form simplified Yang style Tai Chi, 60 minutes per session, 7 sessions per week, for 8 weeks. | Physical performance: strength, flexibility, cardiovascular function, pulmonary function, BMI; |
| Liu YY 2003 | CCS | China | Health promotion | 24-form simplified Yang style Tai Chi, 40 minutes per session, 3 sessions per week, for 8 weeks. | Psychological: general status; |
| Zhang CH 2012 | CCS | China | COPD | 24-form simplified Yang style Tai Chi, 30 minutes per session, 3 sessions per week, for 12 weeks. | Physical performance; |
| Tu HL 2005 | CCS | China | Health promotion | Tai Chi, 12 minutes per session, 3 sessions per week, for 10 weeks. | Physical performance: cardiovascular function; |
| Wang XC 2009 | CCS | China | Health promotion | Yang style Tai Chi, practiced under instruction of an experienced tutor, 40-60 minutes per session, 4-6 sessions per week, for 96 weeks. | Physical performance: cardiovascular function, pulmonary function, BMI; |
| Liu JW 2006 | CCS | China | Health promotion | 24-form simplified Yang style Tai Chi, 30 minutes per session, 2 sessions per day, for 12 weeks. In 4th-5th weeks, Tai Chi philosophy and ideas were added, 120 minutes each session. Participants were instructed to understand Tai Chi by group discussion. | Psychological: general status; |
| Sun XS 1996 | CCS | China | Coronary artery disease | Modified 24-form simplified Yang style Tai Chi | Physical performance: cardiovascular function, BMI; |
| Chen JY 2011 | CCS | China | Hypertension | Tai Chi, 60 minutes per session, 5 sessions per week, for 92 weeks. | Symptom; |
| Gu G 2012 | CCS | China | COPD | 24-form simplified Yang style Tai Chi, 60 minutes per session, 3 sessions per week, for 40 weeks. Each session consisted of warm up, practice and cool down. | QOL:SF-36;Symptom; Physical performance: pulmonary function; |
| Zhao XM 2006 | CCS | China | Health promotion | 24-form simplified Yang style Tai Chi, 30-40 minutes per session, 3-5 sessions per week, for 16 weeks. | Physical performance: cardiovascular function; |
| Zhang L 1994 | CCS | China | Health promotion | Tai Chi, 30 minutes per session, 5 sessions per week, for 48 weeks. | Physical performance: cardiovascular; |
| Li CW 2007 | CCS | China | Health promotion | 24-form simplified Yang style Tai Chi and 48-form Tai Chi, 45 minutes per session, 5 sessions per week, for the first four weeks; then practiced 60 minutes per session, 5 sessions per week for 20 weeks. | Physical performance; |
| Ma ZJ 2012 | CCS | China | Hyperlipidemia | Tai Chi, 30-60 minutes per session, 4 sessions per week, for 24 weeks. | Physical performance; |
| Chen J 2009 | CCS | China | COPD | Tai Chi, 60 minutes per session, 6 sessions per week, for 44 weeks. | Incidence: COPD |
| Sun YX 1988 | CCS | China | Cardiopulmonary disease | Tai Chi, 60 minutes per session, 3 sessions per week, for 72 weeks. | Physical performance: pulmonary function; |
| Kui RQ 1990 | CCS | China | Cardiopulmonary disease | Tai Chi for 72 weeks. | Physical performance: pulmonary function; |
| Gao J 2011 | CCS | China | Health promotion | Tai Chi for 12 weeks. | Psychological: response time; |
| Tan L 2004 | CCS | China | Health promotion | Tai Chi, 30-40 minutes per session, 5-7 sessions per week, for 28 weeks. | Physical performance: cardiovascular function; |
| Liu XD 2010 | CCS | China | Health promotion | 24-form simplified Yang style Tai Chi, 60 minutes per session,4 sessions per week, for 8 weeks. Each session consisted of 10 minutes of warm up, 40 minutes of TC practice and 10 minutes of cool down. | Physical performance: cardiovascular function, pulmonary function; |
| Jin H 2000 | CCS | China | Health promotion | 24-form simplified Yang style Tai Chi, 30 minutes per session, 6 sessions per week, for 48 weeks. | Physical performance: biomarker; |
| Gu G 2011 | CCS | China | COPD | 24-form simplified Yang style Tai Chi, 60 minutes per week, 3 sessions per week, for 12 weeks. | QOL:COPD clinical questionnaire; Physical performance: cardiovascular function, pulmonary function; |
| Zhang WJ 1991 | CCS | China | Health promotion | Tai Chi, 30 minutes per session, practiced under instruction of physical exercise teacher, 6 sessions per week, for 48 weeks. | Physical function: cardiovascular function; |
| Wang YS 1994 | CCS | China | Health promotion | Tai Chi, 30 minutes per session, practiced under instruction of physical exercise teacher, 6 sessions per week, for 48 weeks. | Physical performance: cardiovascular function, balance; |
| Wen HX 2010 | CCS | China | Attention deficit hyperacticity disorder(ADHD) | Tai Chi, 45 minutes per session, 3 sessions per week, for 12 weeks. | Symptom; |
| Tie Y 2008 | CCS | China | Health promotion | 24-form simplified Yang style Tai Chi, 60 minutes per session, 3-5 sessions per week, for 24 weeks. | Incidence: infection of the upper respiratory tract; Symptom; Physical performance: biomarker; |
| Zou WL 1995 | CCS | China | Cerebral arteriosclerosis | 48-form Tai Chi, 60 minutes per session, 7 sessions per weeks, for 144 weeks. | Symptom; Physical performance; |
| Chen J 2011 | CCS | China | Hypertension | Tai Chi, 7 sessions per week, for 48 weeks. | Symptom; |
| Jiang JX 1984 | CCS | China | Arteriosclerosis | 24-form simplified Yang style Tai Chi,40 minutes per session, 3 sessions per week. | Physical performance; |
| Teng GH 2012 | CCS | China | Lumbar disc herniation | Chen-style Tai Chi, 60 minutes per session, 6 sessions per week, for 24 weeks. | Symptom; |
| He JH 2011 | CCS | China | Essential Hypertension | 24-form simplified Yang style Tai Chi, 42-form and 48-form Tai Chi, 40-50 minutes per session, 6 sessions per week, for 20 weeks. | Symptom; |
| Guo SJ 2002 | CCS | China | Health promotion | 24-form simplified Yang style Tai Chi, twice per day, 60 minutes per session, 3 sessions per week, for 96 weeks. At least 5 sessions were required per week. | Physical performance; |
| Liu Q 2011 | CCS | China | Health promotion | Tai Chi for 8 weeks. | Physical performance: perception; |
| Bao XM 2008 | CCS | China | Health promotion | 24-form simplified Yang style Tai Chi, once per week, for 20 weeks. | Psychological: general status; |
| Sun JS 2002 | CCS | China | Neurasthenia | 24-form simplified Yang style Tai Chi, 30 minutes per session，twice per day,14 sessions per week, for 8 weeks. | Symptom; |
| Zhou QQ 2011 | CCS | China | schizophrenia | 24-form simplified Yang style Tai Chi, 2 hours per session, twice per day(in the morning and in the afternoon), 14 sessions per week, for 12 weeks. Each session consisted of 60 minutes of basic movements, 30 minutes of TC practice and 30 minutes of discussion. | Symptom; |
| Zhu XQ 2009 | CCS | China | schizophrenia | 24-form simplified Yang style Tai Chi, 60 minutes per session, 5 sessions per week, for 12 weeks. Each session consisted of 10 minutes of warm up, 40 minutes of TC practice and 10 minutes of cool down. Participants learnt TC during the first 2 weeks, and practiced from the 3rd week. | Symptom; Physical performance; |
| Wang C 2007 | CCS | China | Hypertension | 48-form Tai Chi, 60 minutes per session, for 24 weeks. | Symptom; |
| Deng YM 2001 | CCS | China | Health promotion | 24-form simplified Yang style Tai Chi class, for 1.5 hours. Participants were required to practice by themselves for 30-60minutes per session, 3 sessions per week, for 72 weeks. | Psychological: general status; |
| Zhang LJ 2012 | CCS | China | Arteriosclerosis | 85-form Yang style Tai Chi, 30-50 minutes per session, 5-8 sessions per week, for 72 weeks. | Physical performance: cardiovascular function; |
| Ding Z 2006 | CCS | China | Hyperlipidemia | 24-form simplified Yang style Tai Chi, for 20 weeks. The first 6 weeks were learning period, and the remaining time was practicing period. 50 minutes per session, 5 sessions per week. Each session consisted of 10 minutes of warm up, 30 minutes of TC practice and 10 minutes of cool down. | Symptom; |
| Li JZ 2006 | CCS | China | Coronary artery disease | 24-form simplified Yang style Tai Chi, and 48-form Tai Chi, practiced under instruction of qualified coach, 40 minutes per session, 6 sessions per week, for 24 weeks. | Physical performance; |
| Wang Y 2011 | CCS | China | Hypertension | Tai Chi, 40 minutes per session, for 40 weeks. | Symptom; |
| Wang YL 2010 | CCS | China | Breast cancer | 24-form simplified Yang style Tai Chi, twice per day, 20 minutes per session, for 24 weeks. The time per session could be modified according to health status. | QOL: WHOQOL-BREF; Symptom; |
| Xie YL 2011 | CCS | China | Hyperlipidemia | Tai Chi, 30 minutes per session, 7 sessions per week, for 28 weeks. Each session consisted of 10 minutes of warm up, 10 minutes of TC practice and 10 minutes of cool down. | Symptom; |
| Cai Y 2010 | CCS | China | Depression | Chen style Tai Chi, twice per day (in the morning and in the afternoon), 20-30 minutes per session, for 8 weeks. | Psychological: depression; |
| Zhang W 2007 | CCS | China | Male sexual dysfunction | Tai Chi, 60 minutes per session, 4 sessions per week, for 96 weeks. | Symptom; Physical performance; |
| Li X 2011 | CCS | China | Stroke | Tai Chi, practiced under instruction of therapist, focusing on imagination, 30-35 minutes per session, 5 sessions per week, for 6 weeks. | Physical performance: strength, flexibility; |
| Liu XK 2011 | CCS | China | Health promotion | 24-form simplified Yang style Tai Chi, practiced under instruction of qualified teacher, 2 hours per session, once per week, for 16 weeks. Each session consisted of 45 minutes of learning, 45 minutes of practicing and 30 minutes of rest. | Psychological: depression, anxiety, self-efficacy; |
| Yu JF 2005 | CCS | China | Gynecologic cancer | 4-form Chen style Tai Chi, 60 minutes per session, supervised by qualified nurse, 7 sessions per week, for 1 week. | Physical performance: biomarker; Psychological: general status; |
| Zhang L 1994 | CCS | China | Health promotion | Tai Chi, 30 minutes per session, 5 sessions per week, for 48 weeks. | Physical performance; |
| Gong YH 2012 | CCS | China | Internet addiction | 24-form simplified Yang style Tai Chi, 100 minutes per session, 3 sessions per week, for 12 weeks. Each session consisted of 15 minutes of warm up,75 minutes of practice and 10 minutes of cool down. | Psychological status; |
| Zeng YH 2012 | CCS | China | Health promotion | 24-form simplified Yang style Tai Chi, 30-40 minutes per session, 3 sessions per week, for 96 weeks. | Physical performance: cardiovascular function, BMI; |
| Chen YP 2004 | CCS | China | Hypertension | 24-form simplified Yang style Tai Chi or 42-form Tai Chi, 30-45 minutes per session, 1-2 sessions per day, 4 days per week, for 20 weeks. Before each session, there was a 10-15 minutes of warm up. | Symptom; |
| Zheng J 2011 | CCS | China | Climacteric Syndrome | 24-form simplified Yang style Tai Chi, for 12 weeks. | QOL:MENQOL; Symptom; |
| Lin YB 2009 | CCS | China | Health promotion | 24-form simplified Yang style Tai Chi, 60 minutes per session, 3 sessions per week, for 28 weeks. | QOL; Psychological: general status; |
| Jin M 2012 | CR | China | Spinal cord injury | Tai Chi, practiced when sat in the wheelchair, 90 minutes per session, 3 sessions per week, for 10 weeks. | Psychological: general status; |
| Venglar M 2005 | CR | USA | Parkinson disease | Yang style, 60 minutes per session, once a week for 8 weeks, taught by a Tai Chi instructor certified by Grand Master William C C Chen. Participants were asked to practice for at least 90 minutes each week, and were provided with video and paper supplements. | Incidence: falls; Physical performance: flexibility, balance; Psychological: confidence, self-efficacy; |
| Shapira MY 2001 | CR | USA | Head trauma | Tai Chi training program consisted of gradual steps: sitting, standing, and movement. In the first several weeks training was performed only in the sitting position. The next step was to convince the patients to stand. Several weeks later, the TCC forms were gradually taught, weekly session and then twice a week. Patients were encouraged to train at home, practicing an hour a day, for 2-4 years. | Incidence: falls; Physical performance: strength, flexibility, balance; |
| Kluding P 2006 | CR | USA | Parkinson disease | 4 movements from 24-form simplified Yang style Tai Chi. The first month consisted of participation in a group balance exercise class led by a physical therapist, on hour, twice per week for 4 weeks. The second month, the participants purchased a 3-month membership at a local community-based fitness center to continue exercise. The third month, Tai Chi classes were offered twice each week for 4 weeks. The Tai Chi classes consisted of warm-up activities in standing emphasizing breathing, weight shifting, and trunk rotation, and four Tai Chi movements. | Physical performance: balance; |
| Li YQ 2011 | CS | China | Lumbar muscle strain | 42-form Tai Chi, 40 minutes per session, 7 sessions per week, for 12 weeks. | Symptom; |
| Yu DH 2011 | CS | China | Balance control | 24-form simplified Yang style Tai Chi and 42-form Tai Chi, 60 minutes per session, 4 sessions per week, for 24 weeks. Each session consisted of 10 minutes of warm up, 45 minutes of practice, and 5 minutes of cool down. | Physical performance: balance; |
| Xie YL 2011 | CS | China | Health promotion | 24-form simplified Yang style Tai Chi, 42-form Tai Chi and breathing practice, 60 minutes per session, 4 sessions per week, for 24 weeks. Each session consisted of 10 minutes of warm up, 45 minutes of practice, and 5 minutes of cool down. | Physical performance: cardiovascular function; |
| Zhao JG 2000 | CS | China | Health promotion | 42-form Tai Chi, 1.5 hour per session, 7 sessions per week, for 48 weeks. | Physical function: cardiovascular function; |
| He YF 2009 | CS | China | Lumber muscle strain | 42-form Tai Chi, taught be qualified coach, 40 minutes per session, two sessions per day, for 4 weeks. | Symptom; |
| Wei Y 2007 | CS | China | Health promotion | 48-form Tai Chi, 1-1.5 hour per session, 3 sessions per week, for 24 weeks. | Physical function: cardiovascular function, pulmonary function; |
| Wu HC 2008 | CS | China | Health promotion | Tai Chi, 60 minutes per session, two sessions per day, for one week. | Physical performance: biomarker; |
| Chen KM 2007 | CS | China | Health promotion | 24-form simplified Yang style Tai Chi, 50 minutes per session, 3 sessions per week, for 48 weeks. This study encompasses the three phases of warm-up, Tai Chi, and cool-down. Instructor-led Tai Chi exercises for the first six months and video-guided Tai Chi exercises for the latter six months. One instructor demonstrated and led the program, while the other corrected the positions of the participants and prevented any possible harm. | QOL:SF-36; Physical performance: cardiovascular function, pulmonary function, BMI; Psychological: general status; |
| Liu X 2008 | CS | Australia | Health promotion-diabetes prevention | Kai Mai Tai Chi, participants attended Tai Chi and Qigong exercise training for 1 to 1.5h, 3 times per week for 12 weeks, and were encouraged to practice the exercises at home on days when they did not attend the group sessions. Each session consisted of 28 minutes' warm-up, 30 minutes' practice, and 6 to 28 minutes' cool-down. The intensity of the exercise during each session varied among participants according to individual health and fitness levels. | QOL:SF-36; Physical performance: cardiovascular function, pulmonary function, BMI; Psychological: depression, stress; Compliance; |
| Taylor-Piliae RE 2006 | CS | USA | Health promotion-psychosocial status | 24-form simplified Yang style Tai Chi, 60-minute per session, three times per week for 12 weeks. Each session consisted of a 20-minute warm-up period, 30-minutes of TC exercise and a 10-minute cool-down period. Participants were encouraged to practice the same routine at home at least two other days. | Psychological: stress, mood, self-efficacy, social support; |
| Jahnke RA2010 | CS | USA | Health promotion | 24-form simplified Yang style Tai Chi. Phase I: Training Non expert Practice. The training consisted of studying the Tai Chi Easy Practice Leader Training Manual and practicing the methods to take at least 25 hours. Phase II: After the practice leaders were trained, each committed to teach the 8-to 10-week series of sessions to groups of enrollees, at their respective sessions in i week, but most offering 1 session per week. | Symptom; Psychological: stress; |
| Richerson S 2006 | CS | USA | Balance control | 24-form simplified Yang style Tai Chi, once a week for an hour, for 24 weeks. A certified Tai Chi instructor taught the subjects the first set of the Yang Style of Tai Chi. All training sessions built on one another, and subjects were encouraged to go at their own place. | Physical performance: perception, balance; |
| Ling KW2003 | CS | China | End-stage renal disease | Basic Tai Chi practice, participant were advised to exercise by following the demonstration on the videotape, 30 minutes per session, 3-7 times weekly, for 12 weeks. | QOL: Kidney Disease Quality of Life Short Form[KDQOL-SF]; Physical performance: strength, flexibility; Compliance; |
| Jones AY2005 | CS | China | Health promotion | Cheng 119 style Tai Chi, taught by a Tai Chi master for 1.5 hours, 3 times weekly, for 12 weeks. | Physical performance: flexibility, cardiovascular function, pulmonary function, balance; Satisfaction; |
| Romero-Zurita A2012 | CS | Spain | Fibromyalgia | 24-form simplified Yang style Tai Chi, three 60-minute Tai-Chi sessions weekly for 28 weeks. | QOL:SF-36;Symptom; Physical performance: strength, flexibility, BMI, balance; Psychological: depression, stress, self-esteem, general status, self-efficacy; |
| Taggart HM2003 | CS | USA | Fibromyalgia | 24-form simplified Yang style Tai Chi, 1-hour per session, twice weekly, for 6 weeks. | QOL:SF-36;Symptom; |
| Shen CL2007 | CS | USA | Type 2 diabetes | 24-form simplified Yang style Tai Chi, two 60-minute instructed Tai Chi exercise sessions each week for 12 weeks. | QOL: Diabetes Quality of Life Questionnaire-39; Physical performance; Psychological: self-efficacy; |
| Wang YT2004 | CS | USA | Health promotion-physical and mental health | 24-form simplified Yang style Tai Chi, taught by an experienced Tai Chi instructor, 1-hour per session, twice a week, for 3 months. Each session included 10 minutes of breathing and stretching exercises followed by 50 minutes of Tai Chi. | QOL:SF-36; Physical performance: strength, flexibility; Psychological: general status; |
| Chang RY2011 | CS | China | Health promotion-cardiovascular disease prevention | 24-form simplified Yang style Tai Chi, taught by a professional Yang’s style TC teacher, 60-minute per session. | Physical performance: cardiovascular function, BMI; |
| Shen CL2008 | CS | USA | Knee Osteoarthritis | 24-form simplified Yang style Tai Chi, 60-minutes per session, two sessions per week, instructed by Tai Chi teacher, for 6 weeks. Each class consisted of 10 min of warm-up exercise, 40 min of Tai Chi, and 10 min of cool-down exercise. | Symptom; Physical performance: balance; Psychological: self-efficacy; |
| Barbat-Artigas S2011 | CS | Canada | Type I dynapenia | 24-form simplified Yang style Tai Chi, for 12 weeks. The 12-week intervention program started with a short form composed of 24 basic movements, which then gradually progressed into a sequence of 42 movements. Each session started with a warm-up composed of a sequence of 24 movements. The instructor progressively added one to two movements every week. | QOL:SF-36; Physical performance: strength, cardiovascular function, pulmonary function, BMI, balance; |
| Palasuwan A2011 | CS | France | Health promotion-antioxidant capacity | 24-form simplified Yang style Tai Chi, 75 minutes per session; two sessions per week, for 8 weeks. Each session included 5 min of check-in,10 min of warm-up exercise and 60 min of Tai Chi. In addition, all participants were given a video CD demonstrating Tai Chi postures and were asked to practice twice a week at home, with 60 min per session. | Physical performance: strength, flexibility, cardiovascular function, pulmonary function, BMI, balance; |
| Logghe IHJ2011 | CS | Netherlands | Falls Prevention | Modified Yang style Tai Chi, taught by a professional Tai Chi instructors experienced in teaching Tai Chi in older people, 1 hour per session, twice a week for 13 weeks. Each lesson consisted of 20 min warming-up, 30 min TCC and 10 min cooling down. Participants were asked to practice the learned TCC positions at home at least twice a week for about 15 min per session. | Compliance; |
| Uhlig T2010 | CS | Norway | Rheumatoid arthritis | ‘Twelve Movement Tai Chi for arthritis, taught by two qualified instructors, twice weekly, 60 minutes per session, for 12 weeks. | QOL:SF-36;Symptom; Physical performance: strength, flexibility, BMI, balance; Psychological: fear of falling, self-efficacy; |
| Yao L2013 | CS | USA | Dementia | 24-form simplified Yang style Tai Chi, 60 minutes per session, twice per week for 4 weeks, led by an experienced exercise instructor under the supervision of a certified Tai Chi instructor. After 2 weeks of group training, participants were asked to start an additional session each week at home to promote a smooth transition to the home exercise stage. During the home phase, caregivers facilitated patients practicing three 20-min Tai Chi sessions each week for additional 12 weeks. | Symptom; Physical performance: balance; |
| Lee EO2010 | CS | South Korea | Gastric cancer | 11 combination forms of Sun and Yang style Tai Chi, led by trained tai chi practitioners, weekly, for 24 weeks. Each session consisted of a 10-minute warm-up exercise to loosen the body and Chi Kung exercise, a 30- to 40-minute period of tai chi and chi kung exercise, and a 10-minute cooling down and chi kung exercise. | QOL: HRQOL, Functional Assessment of Cancer Therapy-General; Physical performance: biomarker; Psychological: depression; |
| Wu G 2006 | CS | USA | Balance control | 24-form simplified Yang style Tai Chi, 60 minutes per session, three times per week for 15 weeks. Each class started with a 20-minute warm-up including deep breathing and stretching, followed by Tai Chi, and ended with a 2-minute cool-down including visualization exercises. | QOL:SF-36; Physical performance: balance; Psychological: fear of falling; Satisfaction; |
| Taylor-Piliae RE2006 | CS | USA | Health promotion-cardiovascular disease prevention | 24-form simplified Yang style Tai Chi, led by a proficient TC Instructor, 60-minutes per session, 3 times per week for 12 weeks. Each session consisted of a 20-minute of warm- up period, 30-min of TC exercise, and a 10 -min cool- down period. | Physical performance: strength, flexibility, balance; Compliance; |
| Li L2010 | CS | USA | Peripheral Neuropathy | Yong style Tai Chi, three, one-hour, instructor-led group sessions per week, for 24 weeks. | Physical performance: flexibility, cardiovascular function, balance; |
| Esch T2007 | CS | Germany | Health promotion-stress | 24-form simplified Yang style Tai Chi, once a week, delivered for 90 minutes each by a certified TC teacher. In addition, participants were asked to self-train selected TC exercises once to twice a day. | QOL:SF-36; Physical performance: cardiovascular function; Psychological: stress; |
| Carbonell-Baeza A2011 | CS | Spain | Fibromyalgia | 24-form simplified Yang style Tai Chi, led by a master TC instructor, three 60-minute Tai Chi sessions conducted weekly for 16 weeks. Each session included 15 minutes of warm up 30 minutes of Tai Chi exercise and finally 15 minutes of various relaxation methods. | Incidence: chronic pain; QOL: SF-36; Symptom; Physical performance: strength, flexibility, cardiovascular function, pulmonary function, BMI; Psychological: depression; |
| Yeh SH2006 | CS | China | Health promotion-physical and immune response | Cheng's 37 forms, led by a TCC master with 30 years of experience, 60 minutes with a 10 minute warm up, a 40 minute practice, and a 10 minute cool down. Group sessions were given three times a week for 12 weeks. Each participant also received a take home video tape demonstrating the tai chi 37 forms before the program. | Physical performance: cardiovascular function, pulmonary function, biomarker; |
| Wall RB2005 | CS | USA | Health promotion-stress | 24-form simplified Yang style Tai Chi, led by a certified instructor of Tai Chi, 1 hour per week, for 5 weeks. | Psychological: general status; |
| Li F2007 | CS | USA | Parkinson disease | 24-form simplified Yang style Tai Chi, 90-min per day, for 5 days, led by a instructor, certified in first aid and CPR, with over 8 years of teaching and research experience in Tai Chi. | QOL:SF-12; Physical performance: cardiovascular function, pulmonary function, BMI; Satisfaction; |
| Yeh SH 2007 | CS | China | Type 2 diabetes | Cheng's 37 form TC, 60 minutes per session, 3 sessions per week, for 12 weeks. Each session included a 10-min warm-up, 40-min practice, and 10-min cool down. | Physical performance: biomarker; |
| Lan C2000 | CS | China | Health promotion-muscular strength and endurance | 108-form Yang style Tai Chi, weekly, for 24 weeks. Each session consisted of 20 minutes of warm-up, 24 minutes of structured TCC training, and 10 minutes of cool-down exercises. | Physical performance: strength, flexibility; |
| Reid-Arndt SA2012 | CS | USA | Cancer | 24-form simplified Yang style Tai Chi, led by a licensed occupational therapist, 60-minute per session, two times per week for 10 weeks. | Symptom; Physical performance: strength, balance; Psychological: stress, mood; |
| Tamim H2009 | CS | Canada | Health promotion-musculoskeletal fitness | Tai Chi, led by a professional TC practitioner, 50 minutes per session, two sessions per week, for 12 weeks. | Physical performance: strength, cardiovascular function, BMI; Psychological: stress, general status; |
| Nomura T2011 | CS | Japan | Health promotion-physical function and ability | Tai Chi Yuttari-exercise, once a week for 12 weeks. Each session lasted 90 min including a break time. The instructor for the exercise was a coach from the NPO Osaka Wushu Taijiquan Federation. | Physical performance: strength, flexibility, cardiovascular function; Compliance; Safety |
| Chang JY2011 | CS | USA | Cognitive impairment | Sun-style TC for Arthritis, twice a week for 20–40 minutes per session, for 15 weeks. | QOL: SF-36; Symptom; |
| Tsai PF2009 | CS | USA | Knee Osteoarthritis | 12-form Sun-style TC, led by a certified TC instructor with 4 years of experience, 20-40 minutes per session, twice a week for 15 weeks. Each session included a warm-up, 12-form Sun-style TC, and a cool-down period. | QOL:SF-36; Symptom; |
| Ross MC1999 | CS | USA | Health promotion-movement, pain and mood | Tai Chi, three times each week, for 8 weeks. Each session included 10 minutes of preparation and wrap-up administrative time and 50 minutes of actual Tai Chi movements. | Physical performance: strength, flexibility, balance; |
| Chen KM2008 | CS | China | Health promotion-physical health | 24-form simplified Yang style Tai Chi, led by two certified instructors, three times a week, 50 min per session for 24 weeks. | QOL:SF-36; Physical performance: strength, flexibility, cardiovascular function, pulmonary function, balance; |
| Li FZ2008 | CS | USA | Falls Prevention | Tai Chi, twice per week, led by trained community tai chi instructors, 1 hour per session for 12 weeks. | Incidence: falls; QOL:SF-12; Physical performance: strength, flexibility; Compliance; |
| Chen KM2007 | CS | China | Health promotion-well-being | 24-form simplified Yang style Tai Chi, led by a national-certified Tai Chi master, twice a week for 60 minutes per practice for 24 weeks. | Incidence: falls; QOL:SF-36; Physical performance: cardiovascular function; Psychological: fear of falling, quality of sleep; |
| Gao XF 2011 | CS | China | Insomnia | 42-form Tai Chi, twice per day, 40 minutes per session, for 4 weeks. | Symptom; |
| Liu Y 2007 | CS | China | Defects of vertebral column | Chen style Tai Chi, 5-8 times per day, for 24 weeks. | Symptom; Physical performance: pulmonary function; |
| Yang ZH 2005 | CS | China | Health promotion | Chen style Tai Chi, 60 minutes per session, 5 sessions per week, for 20 weeks. | Physical performance: pulmonary function; biomarker; |
| Jiang Y 2013 | CS | China | Health promotion | 24-form simplified Yang style Tai Chi, 10-20 minutes per session, 5 sessions per week, for 48 weeks. | Psychological: general status: |
| Ji CN 2010 | CS | China | Lumbar disc herniation | 24-form simplified Yang style Tai Chi, 60 minutes per session, 6 sessions per week, for 8 weeks. | Symptom; |
| Deng YX 2012 | CS | China | Ankylosing spondylitis (AS) | Tai Chi for 12 weeks. | Symptom; Physical performance: biomarker; |
| Tian GH 2011 | CS | China | Functional dyspepsia | 42-form Tai Chi, three times per day, at 2 hours after meal, for 4 weeks. | Symptom; |
| Zhongn YJ 2009 | CS | China | Essential Hypertension | 83-form Chen style Tai Chi, 50 minutes per session, once per week, for 16 weeks. | Symptom; Physical performance: cardiovascular function; |
| Sun XS 1998 | CS | China | Health promotion | 24-form simplified Yang style Tai Chi | Physical performance: cardiovascular function; |
| Cao J 2012 | CS | China | Fatty liver | Tai Chi, 60 minutes per session, 4 sessions per week, for 16 weeks. | Symptom; Physical performance; |
| Shen HJ 2012 | CS | China | Oligozoospermia | 24-form simplified Yang style Tai Chi, 50 minutes per session, with 10 minutes of warm up, 30 minutes of practice and 5-10 minutes of cool down, for 24 weeks. | Incidence: partner became pregnant; Physical performance; Psychological: confidence; general status; |
| Zhang YX 2012 | CS | China | Low Back Pain | 24-form simplified Yang style Tai Chi, once daily, for 10 times. | Symptom; |
| Kuang GJ 2005 | CS | China | Chronic Heart Failure | 42-form Tai Chi for 24 weeks. | Physical performance: cardiovascular function; |
| 重庆市结核病医院中西医结合小组 1975 | CS | China | Tuberculosis (TB) | 24-form simplified Yang style Tai Chi, twice per day, for 104 weeks. | Symptom; Physical performance: BMI; |
| Lv JB 1987 | CS | China | Hypertension | 24-form simplified Yang style Tai Chi, twice per day, for 24 weeks. Each session consisted of 30 minutes of Qigong, 15 minutes of TC practice, and 10-15 minutes of cool down. | Symptom; |
| Wang JH 2002 | CS | China | Type 2 diabetes | Tai Chi, 30 minutes per session, once daily, for 8 weeks. | Physical performance: biomarker; |
| Zhou T 2012 | CS | China | Obese | 24-form simplified Yang style Tai Chi, 60 minutes per session, once daily, 5 sessions per week, for 48 weeks. | Physical performance; |
| Chen H 2012 | CS | China | Hypertension | 24-form simplified Yang style Tai Chi, 60 minutes per session, 6 sessions per week, for 10 weeks. | Physical performance: biomarker; |
| Qian JA 1986 | CS | China | Heart disease | Tai Chi, 40 minutes per session daily, for 48 weeks. | Symptom; Physical performance: cardiovascular function; |
| Wang Y 2000 | CS | China | Chronic disease | Tai Chi, 1-2 times per day, 20-40 minutes per session, for 48 weeks. | Physical performance: strength, flexibility, cardiovascular function, pulmonary function, BMI; |
| Wang KH 2010 | CS | China | Health promotion | 24-form simplified Yang style Tai Chi, once per week, for 16 weeks. | Physical performance: biomarker; Psychological: general status; |
| Huang YY 2010 | CS | China | Health promotion | 24-form simplified Yang style Tai Chi, 1.5 hour per session, once per week, for 32 weeks. Participants were required to practice by themselves at least twice per week, 60 minutes per session. | Psychological: general status; |
| Yu WJ 1997 | CS | China | Hypertension | Tai Chi, twice daily, 1-1.5 hour per session, for 144 weeks. | Physical performance: cardiovascular function; |
| Zheng JQ 2004 | CS | China | Coronary artery disease | 24-form simplified Yang style Tai Chi, once daily, for 12 weeks. Each session consisted of warm up, TC practice and cool down. | Physical performance: cardiovascular function; |
| Pan Y 2011 | CS | China | Anxiety | 24-form simplified Yang style Tai Chi, 30 minutes per session, 7 sessions per week, for 6 weeks. | Psychological: anxiety; |
| Ma XP 2003 | CS | China | Health promotion | 24-form simplified Yang style Tai Chi and 48-form Tai Chi, led by professional TC instructor, 50 minutes per session, twice per day, 6 days per week, for 48 weeks. | Physical performance: strength, balance |
| Fu CY 1996 | CS | China | Health promotion | 24-form simplified Yang style Tai Chi, 30 minutes per session, 6 sessions per week, for 48 weeks. | Physical performance: balance; |
| Yu CL 2001 | CS | China | Frozen shoulder | Tai Chi | Symptom; |
| Han YZ 2010 | CS | China | Health promotion | Tai Chi, 60 minutes per session, 4 sessions per week, for 24 weeks. | Physical performance: cardiovascular function, pulmonary function, balance; |
| Wang LL 2010 | CS | China | Health promotion | 42-form Tai Chi, 60 minutes per session, for 12 weeks. 成 | Physical performance; |
| Wu YH 2000 | CS | China | Health promotion | Tai Chi for 24 weeks. | Physical performance: cardiovascular function, pulmonary function; Psychological: general status; |
| Wang LJ 2001 | CS | China | Balance control | 24-form simplified Yang style Tai Chi and 48-form Tai Chi, twice per day, led by processional Wushu instructor, 30 minutes per session, 6 sessions per week, for 48 weeks. | Physical performance: balance; |
| Yin H 2007 | CS | China | Health promotion | Tai Chi, 60 minutes per session, twice per week, for 17 weeks. Participants were required to practice by themselves three times per week. | Symptom; |
| Wang JH 2001 | CS | China | Type 2 diabetes | Tai Chi, 30 minutes per session, once daily, for 8 weeks. | Physical performance; |
| Wu XP 1996 | CS | China | Chronic disease | 24-form simplified Yang style Tai Chi, twice daily, 40 minutes per session, for 12 weeks. | Physical performance: cardiovascular function; |
| Liu SK 2010 | CS | China | Health promotion | 24-form simplified Yang style Tai Chi, 1-2 hours per session, 3-5 sessions per week. | Physical performance: BMI; |
| Ke JB 2007 | CS | China | Health promotion | 24-form simplified Yang style Tai Chi, 40 minutes per session daily for 12 weeks. | Physical performance: cardiovascular function; pulmonary function; BMI; |
| Ren HY 1992 | CS | China | Health promotion | Tai Chi, 60 minutes per session, once daily, for 12 weeks. | Physical performance; |
| Wang XJ 2009 | CS | China | Diabetes | 24-form simplified Yang style Tai Chi, 20-30 minutes per session daily, for 24 weeks. | Psychological: depression, anxiety; |
| Wang LJ 2001 | CS | China | Balance control | Tai Chi, 30 minutes per session, twice daily, 6 days per week, for 48 weeks. | Physical performance: balance; |
| Liu JS 1991 | CS | China | Heart disease | Tai Chi, twice daily, 30-60 minutes per session, for 12 weeks. | Physical performance: cardiovascular function; |
| He YP 2012 | CS | China | Essential Hypertension | Chen style Tai Chi, twice daily, 40 minutes per session, for 12 weeks. | Symptom; Physical performance; |
| Liu J 2009 | CS | China | Health promotion | 24-form simplified Yang style Tai Chi, 60 minutes per session, 3 times per week, for 24 weeks. | Physical performance: biomarker; |
| Ji ZY 2012 | CS | China | Hypertension | 24-form simplified Yang style Tai Chi, 2 hours per session, once daily, for 24 weeks. | Physical performance; cardiovascular function; |
| Sui CF 2012 | CS | China | Climacteric syndrome | 42-form Tai Chi, twice daily, 30 minutes per session, for 12 weeks. | Symptom; |
| Zhang ZR 2012 | CS | China | Health promotion | 24-form simplified Yang style Tai Chi and 48-form Tai Chi, 45 minutes per session, 5 sessions per week, for 20 weeks. Participants were required to practiced by themselves 60 minutes per day and 5 days per week. | Physical performance: cardiovascular function, pulmonary function; |
| Wang DP 2009 | CS | China | Fasciitis | 24-form simplified Yang style Tai Chi and 42-form Tai Chi, 20 minutes per session, twice per day, for 4 weeks. | Symptom; |
| Wang DC 1958 | CS | China | Tuberculosis (TB) | Tai Chi, 20-45 minutes per session, 7 sessions per week. | Symptom; Physical performance: pulmonary function; |
| Zhang LW 2008 | CS | China | Lumbar disc herniation | 24-form simplified Yang style Tai Chi and 42-form Tai Chi, twice daily, 40 minutes per session. | Symptom; |
| Yang DH 2011 | CS | China | Health promotion | 48-form Tai Chi, 1-1.5 hour per session, 3 sessions per week, for 24 weeks. | Physical performance: biomarker; |
| Xu H 2010 | CS | China | Lumbar disc herniation | 13-form simplified Wu style Tai Chi, 60-90 minutes per session, once daily, for 8-12 weeks. | Symptom; |
| Jiang XL 2010 | CS | China | Frozen shoulder | 42-form Tai Chi, three times per day, 20 minutes per session, for 4 weeks. | Symptom; |
| Ni HY 2001 | CS | China | Health promotion | 42-form Tai Chi, 30 minutes per session, for 12 weeks. | Physical performance: cardiovascular function; |
| Li N 2006 | CS | China | Health promotion | 24-form simplified Yang style Tai Chi | Physical performance; |
| Zhao LY 1963 | CS | China | Tuberculosis (TB) | Tai Chi, twice daily, 30 minutes per session, for 12 weeks. | Physical performance: pulmonary function; |
| Ke JB 2009 | CS | China | COPD | 24-form simplified Yang style Tai Chi, 40 minutes per session, once daily, for 12 weeks. | Physical performance; |
| Xia Q 2004 | CS | China | Osteoporosis | 48-form Tai Chi, 40 minutes per session, twice daily, for 48 weeks. | Incidence: falls; Physical performance: balance; |
| Qian YW 1962 | CS | China | Hypertension | Tai Chi, once daily, for 3 weeks. | Symptom; |
| Liu J 2007 | CS | China | Health promotion | 24-form simplified Yang style Tai Chi, 60 minutes per session, 4 sessions per week, for 24 weeks. | Physical performance: biomarker; |
| Liu J 2003 | CS | China | Health promotion | 24-form simplified Yang style Tai Chi, 60 minutes per session, 4 sessions per week, for 24 weeks. | Physical performance: cardiovascular function, pulmonary function; |
| Zhao CF 2008 | CS | China | Lumbar disc herniation | 24-form simplified Yang style Tai Chi and 42-form Tai Chi, twice daily, 40 minutes per session, for 8 weeks. | Symptom; |
| Wang KS 2009 | CS | China | Essential Hypertension | 24-form simplified Yang style Tai Chi and 42-form Tai Chi, twice daily. | Symptom; |
| Ji CN 2010 | CS | China | Fasciitis | 42-form Tai Chi, twice daily, 40 minutes per session, for 4 weeks. | Symptom; |
| Zhang XH 2009 | CS | China | Lumbar disc herniation | 24-form simplified Yang style Tai Chi and 42-form Tai Chi, twice daily, 40 minutes per session, for 4-8 weeks. | Symptom; |
| Gao XF 2009 | CS | China | Essential Hypertension | 24-form simplified Yang style Tai Chi and 42-form Tai Chi, twice daily. The intensity could be modified according to health status. | Symptom; |
| Ni HY 2000 | CS | China | Health promotion | 42-form Tai Chi, 30 minutes per session, 1-2 times daily, for 12 weeks. | Physical performance: cardiovascular function; |
| Chen JF 2013 | CS | China | Stenocardia | 24-form simplified Yang style Tai Chi, 60 minutes per session, 4 sessions per week, for 24 weeks. | Physical performance: cardiovascular function; |
| Du JZ 1961 | CS | China | Dysentery | 24-form simplified Yang style Tai Chi, twice daily, 30 minutes per session. | Symptom; |
| Nie CR 1961 | CS | China | Canker | Tai Chi, twice daily (in the morning and in the afternoon) | Symptom; |
| Wang DW 2009 | CS | China | Type 2 diabetes | 24-form simplified Yang style Tai Chi, 30~40 minutes per session, 3 sessions per week, for 16 weeks. Participants were required to practice by themselves 1-2 times per day, 30 minutes per session at home. | Symptom; Physical performance: BMI; |
| Sun W2012 | RCT | China | Health promotion | 24-form simplified Yang style Tai Chi, 60 minutes per session, 5 sessions per week. Each session consisted of 10 minutes of warm up, 40-45 minutes of practice and 5 minutes of cool down. | Physical performance: pulmonary function; |
| Sun YX1988 | RCT | China | Cardiovascular disease | Tai Chi, 3 sessions per week, for 12 weeks. | Symptom; Physical performance: cardiovascular function; |
| Wu F2010 | RCT | China | Type 2 diabetes | 24-form simplified Yang style Tai Chi, 60 minutes per session before breakfast, 3 sessions per week, for 24 weeks. | QOL:SF-36; Physical performance: biomarker; |
| Burgener SC2011 | RCT | USA | Dementia | Yang style Taiji/Qigong intervention, 1-hour per session, three times weekly for 20 weeks. | Psychological: stress, social support; |
| Tsai PF2013 | RCT | USA | Cognitive Impairment and Osteoarthritic Knee | 12-form Sun style Tai Chi, led by a senior certified TC instructor who had six years' experience in teaching Sun TC and other two certified instructors, three sessions a week, for 20 weeks. TC exercise started at 20 minutes per session and gradually increased to 40 minutes per session. | Symptom; Physical performance: strength, flexibility; |
| Mustian KM2006 | RCT | USA | Breast cancer | Yang style Tai Chi, 60 minutes per session, 3 times per week for 12 weeks, led by an ACSM-certified health and fitness instructor who had extensive training in Yang-style TCC and more than 6 years of experience teaching TCC to varied populations. | Physical performance: strength, flexibility, cardiovascular function, BMI; Compliance; Safety |
| Woo J2007 | RCT | China | Health promotion | 24-form simplified Yang style Tai Chi, three times per week, for 24 weeks. | Physical performance: strength, flexibility, balance; |
| McCain NL2008 | RCT | USA | HIV | Tai Chi, 90-min sessions weekly for 10 weeks, led by an investigator who had received extensive group and individual training from a tai chi master. | QOL: Functional Assessment of HIV Infection; Physical performance: biomarker; Psychological: stress, general status; |
| Kim D2012 | RCT | USA | Fibromyalgia | 8-form Yang-style Tai chi, 90 minutes per session, twice weekly for 12 weeks, led by a Tai chi master with over 30 years of practice and instruction experience. The overall exercise integrated both static and dynamic postures with a progression of increasing time spent in most postures. | Symptom; Physical performance: strength, flexibility; Psychological: quality of sleep, self-efficacy; Compliance; |
| Tsang TW2009 | RCT | Australia | Obese | 24-form simplified Yang style Tai Chi, led by a TC instructor, 60 minutes per session, three sessions per week, for 24 weeks. | Physical performance: strength, BMI; Compliance; |
| Abbott RB2006 | RCT | USA | Headaches | 24-form simplified Yang style Tai Chi, 60 minutes per session, bi-weekly, for 15 weeks, led by an instructor with over 20 years of experience in Tai Chi instruction. | QOL:SF-36;Symptom; |
| Song R2009 | RCT | South Korea | Osteoarthritis | 31 forms of Sun-style Tai Chi, 60 minutes per session, 2 sessions per week to learn TC for 3 weeks from a certified Tai Chi instructor; then 1 session per week for 24 weeks. Participants were encouraged to practice 20 minutes per day at home. | Physical performance: strength, flexibility, BMI; Psychological: fear of falling; |
| Nowalk MP2001 | RCT | USA | Falls Prevention | Tai Chi, three times per week, for 96 weeks, led by a professional instructor. | Incidence: falls; Physical performance: strength, flexibility, ADL, balance; Psychological: depression, stress, fear of falling, cognitive function; |
| Wang CC2010 | RCT | USA | Fibromyalgia | 10 forms from the classic Yang style of tai chi, 60 minutes per session, twice a week for 12 weeks, led by a tai chi master with more than 20 years of teaching experience. Throughout the intervention period, participants were instructed to practice tai chi at home for at least 20 minutes each day. At the end of the 12-week intervention, participants were encouraged to maintain their tai chi practice, using an instructional DVD, up until the follow-up visit at 24 weeks. | QOL:SF-36; Symptom; Psychological: quality of sleep, self-efficacy; Safety |
| Voukelatos A2007 | RCT | Australia | Falls Prevention | Sun, Yang and mixture Tai Chi, 60 minutes per session weekly, for 16 week. Tai Chi instructors have at least 5 years experience teaching tai chi or have completed tai chi trainers’ course, as well as having previously taught tai chi. | Incidence: falls; Physical performance: balance; |
| Chan K2004 | RCT | China | Postmenopausal osteopenia | Yang style Tai Chi, 45 minutes a day, 5 days a week, for 48 weeks. | Incidence: fracture; Physical performance: BMI; Compliance; |
| Gemmell C2006 | RCT | New Zealand | Brain injury | 5 movements from 38-form of Chen style Tai Chi, led by fully qualified instructors, twice weekly for 45 minutes over 6-weeks. | QOL:SF-36; Psychological: mood, self-esteem; |
| Wayne PM2013 | RCT | USA | Health promotion | Tai Chi, 30 minutes per session, two times per week for 24 weeks. Participants were also asked to practice a minimum of 30 minutes, two additional days per week. DVDs or printed materials were provided to facilitate home practice. | QOL:SF-36; Symptom; Physical performance: strength, flexibility, cardiovascular function, balance; Psychological: mood; Compliance; Safety |
| Barrow DE2007 | RCT | UK | Heart Failure | Tai Chi, 55 minutes per session, two sessions per week, for 16 weeks. Each session consisted of Chi Kung exercises, rest period, Tai Chi practice, and cooling down. | QOL: Minnesota living with Heart Failure questionnaire; Physical performance: strength, flexibility, cardiovascular function, pulmonary function; Psychological: mood; |
| Li FZ2001 | RCT | USA | Health promotion | 24-form simplified Yang style Tai Chi, 60 minutes per session, twice a week for 24 weeks. Each practice session consisted of a 15-min warm-up, 30 min of Tai Chi, and a 15-min cool-down period. Three Tai Chi instructors involved in the study; each had taught Tai Chi in the community for at least 7 years (M = 8.4). | QOL: Short-Form General Health Survey(SF-20); Compliance; |
| Caminiti G2011 | RCT | USA | Heart Failure | Modified 10-movement Yang style, led by an experienced Tai Chi instructor similar in style to the routine proposed by Wolf, 50 minutes per session, for 12 weeks. Every session included 10 minutes of warm-up exercises, 30 minutes of Tai Chi practice, and 10 minutes of cool down exercises. | QOL: MacNew QLMI; Physical performance: strength, flexibility, cardiovascular function, balance; |
| Irwin MR2007 | RCT | USA | Varicella Zoster Virus | Tai Chi, 40 minutes per session, given three times per week for 16 weeks. | QOL:SF-36;Symptom; Physical performance: biomarker; Psychological: depression; Compliance; |
| Wolfson L1996 | RCT | USA | Balance control | Tai Chi, 1hour per session, once per week, for 24 weeks. | Physical performance: strength, flexibility, balance; |
| James A2012 | RCT | China | Health promotion | Tai Chi, 50 minutes per session, three times per week in the morning, for 40 weeks. Each session included 20 min of warm-up exercises, 20 min of Tai Chi practice, and 10 min of cool-down exercise. | Physical performance |
| Channer KS1996 | RCT | UK | Acute myocardial infarction | Wu style Tai Chi, led by an instructor, twice weekly for three weeks then weekly for a further five weeks. Each session was of one hour duration with one five-minute break. | Physical performance: cardiovascular function; |
| Jing XL2009 | RCT | Canada | Health promotion | 24-form simplified Yang style Tai Chi, organized and monitored by a qualified TC instructor. In the first six weeks, the subjects were taught the 24-form TC for at least four sessions per week, 60 min per session. Each session included a warm-up for 10 min, learning new forms for 20 min, reviewing for 20 min, and cool-down for 10 min. In the subsequent 10 weeks, subjects practiced TC every morning for 1 h guided by the instructor. Each session included a 5 min warm-up, 50 min practice and refinement, and 5 min cool-down. | Physical performance: strength, flexibility; Compliance; |
| Hammond A2006 | RCT | UK | Fibromyalgia | Tai Chi, started initially with a 15-min warm-up from the Tai Chi for Arthritis program in week 2 and increased to 45-min of combined exercises by meeting 10. Participants were allowed to pace themselves within sessions and a Tai Chi for Arthritis video or DVD was provided to support practice at home. home walking program was recommended to be followed, increasing up to at least 30 min on five days per week. | Symptom; Psychological: self-efficacy; Compliance; |
| Hui CH2009 | RCT | China | Falls Prevention | Tai Chi movements designed by the coaches and the researcher, three times per week for 40 minutes in the early morning, consisted of 13 simple Tai Chi Chuan movements. | Incidence: falls; Physical performance: strength, flexibility; Psychological: fear of falling; |
| Taylor-Piliae RE2011 | RCT | USA | Stroke | 24-form simplified Yang style Tai Chi, 60 minutes per session, three times per week for 12-weeks. Each session consisted of a 20-minute warm-up period, 30-minutes of Tai Chi exercise, and a 10-minute cool-down period. The Tai Chi instructor for this study is a long-term practitioner of the Yang Style of Tai Chi, certified to teach the Yang Style of Tai Chi with over 30 years of teaching experience, including older adults with chronic diseases. | QOL:SF-36; Physical performance: strength, flexibility, cardiovascular function, balance; Psychological: depression, quality of sleep; Compliance; |
| Wu G2010 | RCT | USA | Falls Prevention | 24-form simplified Yang style Tai Chi, led by a certified instructor, 60 minutes per week, 3 sessions per week, for 15 weeks. | Incidence: falls; Symptom; Physical performance: strength, flexibility, balance; Psychological: fear of falling; Compliance; |
| Shen CL2007 | RCT | USA | Health promotion | 24-form simplified Yang style Tai Chi, led by an experienced instructor, 40 minutes per session, 3 sessions per week for 24 weeks. | Physical performance; |
| Lavretsky H2011 | RCT | USA | Depression | Tai Chi, 2 hours per session, once a week. Each TCC class was conducted in 120 minutes and also included 10 minutes of warm-up (e.g., stretching and breathing) and 5 minutes of cool down exercises. | QOL:SF-36; Physical performance: cardiovascular function, BMI; Psychological: depression, mood; Satisfaction; Compliance; Safety |
| Li FZ2002 | RCT | USA | Health promotion | Participants in the intervention group attended a 60-minute Tai Chi session twice a week for 6 months. | QOL: Physical function(SF-GHS); Psychological: depression; Compliance; |
| Shen CL2012 | RCT | USA | Postmenopausal osteopenia | 24-form simplified Yang style Tai Chi, 60 minutes per session, three sessions per week, for 24 weeks. | Symptom; Physical performance: strength, cardiovascular function, BMI; |
| Maciaszek J2012 | RCT | Poland | Balance control | 24-form simplified Yang style Tai Chi, led by a certified Tai Chi instructor, 45 minutes per session, twice a week, for 18 weeks. It included ten minutes of warm up exercise, 30 minutes of Tai Chi practice, and five minutes of cool down exercise. We used five sequences of movement chosen from the simplified 24 forms of Tai Chi. | Physical performance: balance; |
| Maciaszek J2007 | RCT | Poland | Balance-Osteoporosis | 24-form simplified Yang style Tai Chi, 45 minutes per session, twice a week in the morning, for 18 weeks. Each session included 10 min of warm up exercise, 30 min of Tai Chi practice, and 5 min of cool down exercise. | Physical performance: balance; |
| Chan AWK2010 | RCT | China | COPD | Tai Chi, 60 minutes per session, twice a week for 12 weeks. 13 movements of Breathing Regulating Tai Chi were selected and modified from the 18 movements of Tai Chi Qigong produced by the Department of Health. Participants were also advised to practice for an hour everyday apart from the two sessions. DVD and pictures were given to each subject to facilitate daily self practice. | QOL: St George's Respiratory Questionnaire; Psychological: social support; |
| Taylor D2012 | RCT | New Zealand | Falls Prevention | Modified 10-form Sun style, 60 minutes per session, once or twice a week for 20 weeks. | Incidence: falls; Physical performance: strength, flexibility, balance; |
| Zhang Y2008 | RCT | China | Type 2 diabetes | 24-form simplified Yang style Tai Chi, 1 hour per day, 5 days a week for 14 weeks. Each session consisted of 10 min warm-up and then exercised under the supervision of a Tai Chi coach. | Physical performance: cardiovascular function; |
| Li JX2008 | RCT | China | Health promotion | Tai Chi, 60 minutes per session, 4 sessions per week, for 16 weeks. | Physical performance, strength, flexibility, balance; Compliance; |
| Lelard T2010 | RCT | France | Health promotion | 10 movements of Tai Chi specially adapted for the older people, 30 minutes per session, 2 sessions per week, for 12 weeks. | Physical performance: strength, flexibility; |
| Irwin MR2003 | RCT | USA | varicella zoster virus | Tai Chi, led by a instructor with 20 years of experience, 45 minutes per session, 3 sessions per week, for 15 weeks. Each session consisted of a 10-minute warm-up, a 30-minute practice, and a 5-minute cool-down. | QOL:SF-36; Physical performance: biomarker; |
| Peppone LJ2010 | RCT | USA | Breast cancer | 15 movements of Yang style Tai Chi, 3 times per week, 60 minutes per session, for 12 weeks. The instructor was extensively trained in Yang-style TCC and had more than 6 years of TCC teaching experience in a variety of populations. | Physical performance: strength, flexibility, pulmonary function, BMI; |
| Song R2007 | RCT | South Korea | Osteoarthritis | Sun style Tai Chi, 60 minutes per session, three times a week for the first two weeks until they learned the 12 forms of Tai Chi movement step-by-step enough to perform them correctly by themselves at home. From the third week to the last week, the exercise group came to the supervised exercise session once a week, but they were expected to perform Tai Chi movement 3-4 times a week at home for another 10 weeks. | QOL: Health Behavior Scale for cardic patient; Symptom; Physical performance: strength, flexibility, cardiovascular function; |
| Faber MJ2006 | RCT | Netherlands | Falls Prevention | Tai Chi, started with 1 session per week for 4 weeks, followed by twice-weekly sessions for 16 weeks. Each session lasted 90 minutes, including a 30-minute social component of sitting together with a drink, intended to maintain and increase motivation. The first 4 sessions were meant to familiarize the group with the aim of the program and with the exercises. | Incidence: falls; Physical performance: strength, flexibility, ADL, balance; Compliance; |
| Dechamps A2010 | RCT | France | Health promotion | 24-form simplified Yang style Tai Chi, 4 sessions of 30 minutes a week over 24 weeks. | Physical performance: strength, flexibility, ADL; Psychological: mood; Compliance; |
| Thomas GN2005 | RCT | China | Health promotion | 24-form simplified Yang style Tai Chi, led by a registered Tai Chi master, three times per week for 1 hour per session. The subjects were encouraged to practice the Tai Chi forms between classes. | Physical performance: cardiovascular function; |
| Taylor-Piliae RE2010 | RCT | USA | Health promotion | 24-form simplified Yang style Tai Chi, taught by a Tai Chi grand master. A new posture was taught each week during the first 12 weeks, with the entire sequence of postures practiced together during the remainder of the adoption phase. During the maintenance phase 12 additional postures were taught, with participants learning a total of 24 postures. Each class consisted of 45 min of instruction including warm-up exercises, with chairs available for participants to rest as needed. | Physical performance: strength, flexibility, cardiovascular function, balance; Psychological: cognitive function; Compliance; |
| Janelsins MC2011 | RCT | USA | Breast cancer | 15-movement short form of Yang-style Tai Chi, led by a instructor extensively trained in Yang-style TCC and had more than six years of teaching experience. 60 minutes per session, for 12 weeks. Each session consisted of a 10-minute warm-up, followed by a 40-minute TCC, and 10 minutes of structured breathing, imagery, and meditation. | Physical performance: BMI; |
| Song R2003 | RCT | South Korea | Osteoarthritis | Sun-style Tai Chi developed by Lam and colleagues, 20-30 minutes per session, 3 times a week for 12 weeks either with instructors or by themselves. | QOL:SF-36;Symptom; Physical performance: strength, flexibility, cardiovascular function, balance; |
| Chyu MC2010 | RCT | USA | Postmenopausal osteopenia | 24-form simplified Yang style Tai Chi, three 60-minute instructed sessions each week for 24 weeks. Each session consisted of 10 minutes of warm-up, 45-minute training and 5 minutes of cool down. | Incidence: falls; QOL:SF-36; Physical performance: strength, flexibility, balance; |
| Courtney D2009 | RCT | USA | Health promotion | 24-form simplified Yang style Tai Chi, 1.5-hour session, twice per week, for 12 weeks. | Physical performance: perception; |
| Tsang T2008 | RCT | Australia | Type 2 diabetes | Sun and Yang style Tai Chi, 60 minutes per session, two supervised sessions per week, for 16 weeks. | Physical performance: cardiovascular function;, BMI; Compliance; |
| Li DX2013 | RCT | China | Protracted Abstinence Syndrome | Tai Chi, a 60-minute per session every two days, for 24 weeks. Each session consisted of a 10 min of warm-up, 1 h of Tai Chi supervised by a professional coach, and 10 min of cool-down exercises. | Symptom; Physical performance: biomarker; |
| Chang RY2010 | RCT | China | Coronary artery disease | 108-form Yang style Tai Chi, weekly 90-min sessions for 24 weeks. Each session was preceded by 25-min warm-up, and followed by a 90-min TC practice with light classic Chinese music playing in the background. During the rest of the week, the participants were encouraged to practice TC at home on their own at least twice a week using a 50-min video CD provided by the TC instructors. | Physical performance: cardiovascular function; |
| Lu X2013 | RCT | China | Health promotion | 12-forms Yang-style Tai Chi, led by an experienced instructor, 1.5 hour per session, three sessions per week. Each session involved a 15-minute warm-up, 1 hour of Tai Chi practice, and 15 minutes of cooling-down. | Physical performance: strength, flexibility, cardiovascular function; |
| Liu J2012 | RCT | China | Health promotion | 24-form simplified Yang style Tai Chi, led by a Tai Chi master with more than 10 years of teaching experience, 45 minutes per session, twice weekly for 16 weeks. Each session included 5 min of warm-up and a review of TC principles, 30 min of TC movement, 5 min of breathing techniques, and 5 min of cool down. | Physical performance: strength, flexibility, perception; Satisfaction; |
| Yeh GY2004 | RCT | USA | Heart Failure | Five core movements, adapted from Yang-style short form, led by an experienced instructor, 60 minutes per session, twice weekly for 12 weeks. Patients were encouraged to practice at home at least three times per week. | QOL: Minnesota Living with Heart Failure Questionnaire; Physical performance: strength, flexibility, cardiovascular function; Safety |
| Gloria Y2008 | RCT | USA | Heart Failure | Five core Tai Chi movements were adapted from Master Cheng Man Ching’s Yang-style Tai Chi, The intervention consisted of one-hour classes held twice weekly for 12 weeks. Patients were encouraged to practice at home with a 35 min instructional videotape at least three times per week. | QOL: Minnesota Living with Heart Failure Questionnaire; Physical performance: cardiovascular function, pulmonary function; Psychological: quality of sleep; |
| Blake H2009 | RCT | UK | Brain injury | 18-style form of Tai Chi Qigon, led by a certified instructor, 1 hour per session weekly over eight weeks. Each session started with chair-based stretching and warm-up followed by work in self-hand massage reflexology. | Symptom; Psychological: mood, self-esteem, social support; |
| Li FZ2005 | RCT | USA | Falls Prevention | 24-form simplified Yang style Tai Chi, 60 minutes per session, 3 sessions per week, for 24 weeks, taught by experienced Tai Chi instructors who followed the classical 24-Form Yang style. Each session included 5–10 min of warm-up, approximately 45 min of Tai Chi practice, and 5–10 min of cool-down. Each class practice session included musical accompaniment. | Psychological: fear of falling, self-efficacy; |
| Campo RA2013 | RCT | USA | Cancer | Westernized form Tai Chi, 60-minute per session, 3 times a week, for 12 weeks, led by an experienced instructor certified in this form. It consisted of 19 simple, repetitive, nonstrenuous movements and 1 standing pose. | QOL:SF-36; Satisfaction; Compliance; |
| Shen CL2009 | RCT | USA | Postmenopausal osteopenia | 24-form simplified Yang style Tai Chi, taught by a qualified instructor, 60 minutes per session, three times a week, for 24 weeks. Each session consisted of 10 minutes of warm-up exercise, 40 minutes of TC, and 10 minutes of cool-down exercise. | Physical performance; Compliance; Safety |
| Shen CL2010 | RCT | USA | Postmenopausal osteopenia | 24-form simplified Yang style Tai Chi, 60 minutes per session, 3 sessions per week, for 24 weeks. | QOL:SF-36; Physical performance: cardiovascular function, BMI; Psychological: depression; Compliance; Safety |
| Brismee JM2007 | RCT | USA | Knee Osteoarthritis | 24-form simplified Yang style Tai Chi, 40 minutes per session, 3 sessions per week, for six weeks, followed by another six weeks (weeks 7 to 12) of home video Tai Chi practice at the same frequency. | Symptom; Physical performance: flexibility; Safety |
| Tsang T2007 | RCT | Australia | Type 2 diabetes | 12 movements from Sun and Yang style Tai Chi, 60 minutes per session, 2 sessions per week, for 16 weeks. Each session commenced with warm-up exercises (10 minutes), cool-down exercises (5 minutes), and 45 minutes of Tai Chi practice and guidance in breathing techniques and visualization. | QOL:SF-36; Physical performance: strength, flexibility, cardiovascular function, BMI, balance; Psychological: cognitive function; Compliance; |
| Hackney ME2009 | RCT | USA | Parkinson disease | 37 postures of the Yang Short Style of Cheng Manching, led by an experienced instructor, 20 one-hour sessions within 13 weeks. | QOL: Parkinson Disease QUESTIONNAIRE-39; |
| Sprod LK2012 | RCT | USA | Breast cancer | 24-form simplified Yang style Tai Chi, led by an American College of Sports Medicine certified health and fitness instructor, with previous experience, 60 minutes per session, 2 sessions per week, for 12 weeks. Each session consisted of a 10-min warm-up, 40 min of tai chi and 10 min of guided breathing, imagery, and meditation. | QOL:SF-36; Physical performance; |
| Dechamps A2009 | RCT | France | Health promotion | 24-form simplified Yang style Tai Chi, 30 minutes per session, 4 sessions per week, for 24 weeks. Deep breathing  and traditional TC muscle-reinforcement exercises were integrated in the TC routine. The instructor highlighted the TC theory of mind–body balance, which focuses on cognitive and behavioral strategies to strengthen determination. | QOL:SF-12; Physical performance: strength, flexibility; Psychological: self-efficacy; Compliance; Safety |
| Gatts SK2007 | RCT | USA | Balance-impaired | 12 movements of Yang style Tai Chi, 1.5 hour per day, 5 days per week for 3 weeks. | Physical performance: strength, flexibility; |
| Wayne PM2012 | RCT | USA | Postmenopausal osteopenia | Tai Chi, two sessions per week for the first month, and one session per week for eight months thereafter (minimum class duration of one hour). Participants were asked to practice an additional two times per week during the first month, and three times per week thereafter (minimum of 30 minutes per session), which could be home practice or additional sessions at their school. A total of 99.5 hours over the 9 month interventions. | QOL:SF-36, Menopause Quality of Life; Physical performance: strength, flexibility, balance; Compliance; |
| Day L2012 | RCT | Australia | Impairment, Functional Limitation, and Disability | Modified Sun style Tai Chi (46 movements), led by qualified leaders, 60 minutes per session, twice weekly for 24 weeks. | Physical performance: strength, flexibility, cardiovascular function; Psychological: depression; Safety |
| Lam P2008 | RCT | Australia | Type 2 diabetes | Modified Yang and Sun style Tai Chi (20 movements), 1 hour per session, two sessions per week, for 12 weeks; then once per week for a further 12 weeks. | Physical performance; |
| Irwin MR 2008 | RCT | USA | Insomnia | Tai Chi, 40 minutes per session, 3 times per week for 16 weeks. A total 120 minutes of weekly instruction. | Psychological: depression, quality of sleep; |
| Wolf SL2003 | RCT | USA | Falls Prevention | Tai Chi, two sessions a week at increasing durations starting at 60 minutes contact time and progressing to 90 minutes over 48 weeks. The actual ‘‘work’’ time, exclusive of warm up and cool down, progressed from approximately 10 minutes to 50 minutes over 48 weeks. One instructor was a TC grand master, and the other was his student, who had studied with him for more than 5 years. | Incidence: falls; QOL: Sickness Impact Profile; Physical performance: strength, flexibility, BMI, perception; Psychological: depression, fear of falling; Compliance; Safety |
| Lam LCW2011 | RCT | China | Progressive cognitive decline | 24-form simplified Yang style Tai Chi, led by a Tai Chi master. (1) Induction phase—The instruction course for exercise lasted for 8 to 12 weeks. Instructors would conduct weekly teaching until the participants became familiarized with the exercise intervention. (2) Maintenance phase—After the induction course, participants were given a video CD with either the Tai Chi or control exercise program. The participating centers continued to provide arrangement for regular exercise practice. The instruction for frequency of the exercise instructions was no less than 30 min per day and no less than three times per week. | Symptom; Physical performance: balance; Psychological depression; |
| Tsang TW2010 | RCT | Australia | Obese | 24-form simplified Yang style Tai Chi, three one-hour sessions each week, for 24 weeks. | Physical performance: strength, flexibility, cardiovascular function; Safety |
| Logghe IHJ2009 | RCT | Netherlands | Falls Prevention | 24-form simplified Yang style Tai Chi, led by a professional instructor, 1 hour per session, twice a week for 13 weeks. | Incidence: falls; Physical performance: strength, flexibility, cardiovascular function; Psychological: self-efficacy; |
| Irwin MR2012 | RCT | USA | Cellular Inflammation | Tai Chi, led by master’s level TCC instructors, 40 minutes per week, 3 times per week for 16 weeks. A total 120 minutes of weekly instruction. | Physical performance; Psychological: depression, quality of sleep; |
| Qian GQ2012 | RCT | USA | Postmenopausal osteopenia | 24-form simplified Yang style Tai Chi, taught by a qualified instructor, 60 minutes per session, three times a week, for 24 weeks. Each session consisted of 10 minutes of warm-up exercise, 40 minutes of TC, and 10 minutes of cool-down exercise. | Physical performance; |
| Rhonda ORR2006 | RCT | Australia | Type 2 diabetes | 12-movement hybrid from Sun and Yang styles, 55 minutes per session, 2 sessions per week, for 16 weeks. | QOL:SF-36; Physical performance: strength, flexibility, cardiovascular function, BMI, balance; |
| Fransen M 2007 | RCT | Australia | Osteoarthritis | Sun style Tai Chi, 1 hour per session, twice a week for 12 weeks. | QOL:SF-12;Symptom; Physical performance: strength, flexibility; Psychological: depression, stress, anxiety; |
| Pluchino A2012 | RCT | USA | Falls Prevention | Sun style Tai Chi, led by a certified Tai Chi exercise leader, 60 minutes per week, twice a week for 8 weeks, for a total of 16 visits. Each visit consisted of 5 minutes warm-up, 50 minutes of the training, and 5 minutes of cool down. | Physical performance: strength, flexibility; Psychological: fear of falling; |
| Dechamps A2009 | RCT | France | Obese | 24-form simplified Yang style Tai Chi, taught by an experienced instructor, 2-hour session once a week, for 10 weeks. | Physical performance: strength, flexibility, BMI; Psychological: mood, general status, self-efficacy; |
| Robins JLW2013 | RCT | USA | Breast cancer | Tai Chi, 90 minutes each week for 10 weeks. | QOL: Functional Assessment of Cancer Therapy-Breast; Physical performance: biomarker; Psychological: depression; |
| Nguyen MH2012 | RCT | Germany | Health promotion | 24-form simplified Yang style Tai Chi, 60 minutes per session, twice a week for 24 weeks. The session consisted of a 15-minute warm-up and a 15-minute cool-down period. | Psychological: fear of falling, quality of sleep; |
| Tzu TH 2010 | RCT | China | Falls Prevention | Tai Chi consisted of ten positions derived from the Yang style, taught by a professional Tai Chi instructor, who were experienced in working with older persons, five times a week, for 8 weeks. Each session consisted of warm up (10 minutes), teaching and practicing of Tai Chi program (45 minutes), and cool down (5 minutes). | Incidence: falls; QOL: WHOQOK-BREF; Physical performance: strength, flexibility; Psychological: fear of falling, social support; |
| Sattin RW2005 | RCT | USA | Falls Prevention | 6 movements from the 24-form simplified Yang style Tai Chi, two sessions per week at increasing durations starting at 60 minutes contact time and progressing to 90 minutes over the course of 48 weeks. The actual work time, exclusive of warm up and cool down, progressed from about 10 minutes to 50 minutes over the course of the 48weeks. One instructor was a tai chi grand master, and the other was his student, who had studied with him for more than 5 years. | Incidence: falls; Physical performance: strength, flexibility, ADL, balance; Psychological: depression; Compliance; |
| Wang R2013 | RCT | China | Lung cancer | 24-form simplified Yang style Tai Chi, taught by an expert Tai Chi practitioner with more than 20 years of experience, 60 minutes per session, 3 sessions per week, for 16 weeks. | Physical performance: biomarker; |
| Wolf SL2003 | RCT | USA | Falls Prevention | 10 movements from 108-form Yang style Tai Chi, 45 minutes per session, 1 session per week, for 15 weeks. Subjects were requested to practice 15 minutes per session, 2 sessions per week at home. | Incidence: falls; Physical performance: strength, flexibility, cardiovascular function, BMI, ADL, balance; Psychological: depression, fear of falling; Compliance; |
| Kutner NG1997 | RCT | USA | Health promotion | 10 movements from 108-form Yang style Tai Chi, taught by an experienced instructor, 45 minutes per session, twice per week, for 15 weeks. | QOL:SF-36; Physical performance: ADL; Psychological: confidence; self-esteem; |
| Au-Yeung SSY2009 | RCT | China | Balance-Stroke | 12 movements of Sun style Tai Chi, 1 hour per session weekly for 12 weeks, during which subjects practiced 1 to 3 new Tai Chi forms in addition to the forms learnt in previous weeks. After each group practice, each participant performed  approximately 3 hours of self-practice per week. During self-practice, subjects could refer to a video clip of their group practice and to pictures of the Tai Chi forms, with the care givers providing supervision on a standby mode. In general, the subjects could learn the 12 forms in 8 weeks so that they practiced the whole short-form Tai Chi in the remaining 4 weeks. | Physical performance: strength, flexibility, perception; |
| Fu ZL 2005 | RCT | USA | Falls Prevention | 24-form simplified Yang style Tai Chi, taught by experienced Tai Chi instructors, 1 hour three times weekly for 24 weeks. Sessions included 5 to 10 minutes of warm-up, Tai Chi practice, and 5 to 10 minutes of cool-down. Each practice session included musical accompaniment. | Incidence: falls; Physical performance: strength, flexibility, balance; Psychological: fear of falling; |
| Greenspan AI2007 | RCT | USA | Health promotion | 6 simplified forms of Tai Chi, 2 sessions per week at increasing durations starting at 60 minutes of contact time and progressing to 90 minutes over the course of 48 weeks. | Physical performance: strength, flexibility; Psychological: general status, quality of sleep; Compliance; |
| Li FZ2012 | RCT | USA | Parkinson disease | 8 movements of Tai Chi, 60 minutes per session, twice weekly for 24 weeks. | Incidence: falls; Physical performance: strength, flexibility, balance; |
| Fu ZL2004 | RCT | USA | Health promotion | 8-form Easy Tai Chi derived from the 24-form simplified Yang style Tai Chi, led by a well qualified instructor, 1 hour per session, three times per week, for 24 weeks. Regulated breathing was also emphasized as part of the exercise and integrated into the tai chi movement routine. Sessions included 5 to10 minutes of warm- up, tai chi practice, and 5 to10 minutes of cool-down. Instruction covered learning new forms and reviewing forms learned in previous sessions. Each session included musical accompaniment. | QOL: SF-12; Symptom; Physical performance: strength, flexibility; Compliance; Safety |
| McGibbon CA2004 | RCT | USA | Falls Prevention | Five movements of Yang-style Tai Chi, 70 minutes per session, once weekly, for 10 weeks. | Physical performance: strength, flexibility, balance; |
| McGibbon CA2005 | RCT | Canada | Vestibular disorders | 24-form simplified Yang style Tai Chi, 70 minutes per session, once weekly, for 10 weeks. | Physical performance: strength, flexibility, balance; |
| Motivala SJ 2006 | RCT | USA | Health promotion | 20 movements of Tai Chi, 20 minutes per session, once daily, for 25 weeks. | Physical performance: cardiovascular function; |
| Hart J2004 | RCT | Israel | Balance-Stroke | Tai Chi, taught by a certified TCC instructor, 1 hour per session, twice weekly for 12 weeks. | Physical performance: strength, flexibility, balance; |
| Chang YF2008 | RCT | China | Asthma | Classical Chen 32-style Tai Chi, 40 minutes per session, 3 times a week for 12 weeks. | Symptom; Physical performance: pulmonary function; |
| Karen M2004 | RCT | USA | Breast cancer | 15-movement from Yang style Tai Chi, 60 minutes per session, 3 sessions per week, for 12 weeks. Each session consisted of 10 minutes of warm up, 40 minutes of TCC, and 10 minutes of instructed regulatory breathing, imagery, and meditation in order to enhance their TCC skills and provide an exercise cool-down. The instructor was an American College of Sports Medicine certified health and fitness instructor, who was also an experienced TCC instructor. | QOL: Functional assessment of chronic illness therapy-fatigue; Physical performance: BMI; Psychological: self-esteem; |
| Rosado-Perez J2012 | RCT | USA | Health promotion | Tai Chi, led by appointed instructors, 1 hour per session, three sessions per week, for 24 weeks. | Physical performance; |
| Caminiti G2011 | RCT | Italy | Heart Failure | Modified 10-movement Yang style Tai Chi, 30 minutes per session, 2 sessions per week, for 12 weeks. Every session included 10 minutes of warm-up exercises, 30 minutes of Tai Chi practice, and 10 minutes of cool down exercises. | QOL: MacNew QLMI; Physical performance: strength, flexibility, biomarker; |
| Yeh GY2010 | RCT | USA | COPD | Modified TC adapted from Cheng Man-Ching's Yang-style Tai Chi, taught by 2 certified and experienced instructors (average experience of 20 y), 1 hour per session, 2 sessions per week, for 12 weeks. | QOL: Chronic Respiratory Disease Questionnaire; Symptom; Physical performance: strength, flexibility, pulmonary function; Psychological: mood, general status; |
| Hall AM2011 | RCT | Australia | Low Back Pain | Tai Chi, led by a certified tai chi instructor, 2 times per week for 8 weeks followed by once per week for 2 weeks. | QOL:PSFS; Symptom; Psychological: mood; Compliance; Safety |
| Yeh GY2011 | RCT | USA | Heart Failure | 24-form simplified Yang style Tai Chi, led by 6 total study instructors with average experience of 20 years, 1 hour per session, twice weekly for 12 weeks. We provided a 35-minute instructional videotape that outlined the exercises presented in class. Patients were encouraged to practice at home at least 3 times per week. | QOL: Minnesota Living with Heart Failure Questionnaire; Symptom; Physical performance: strength, flexibility; Psychological: mood, self-efficacy; Safety |
| Wang WC2010 | RCT | Japan | Cerebalvascular disorder | 24-form simplified Yang style Tai Chi, 50 minutes per session, once a week for 12 weeks. Each session included 10 min of warm-up and a review of Tai Chi principles, 30 min of Tai Chi practice and 10 min of cool down. | QOL:GHQ-60; Physical performance; Psychological: quality of sleep; |
| Jung S2012 | RCT | South Korea | Prostate hypertrophy | 11 basic and 9 combined movements (20 in total) developed by Dr. Paul Lam, 60 minutes per session, 3 times weekly for 12 weeks. Each session consisted of a warm up (15 min), 20 main movements (40 min), and a cool down (5 min). | QOL: Urination-related quality of life; Symptom; Physical performance; |
| Palumbo MV2012 | RCT | USA | Health promotion | 24-form simplified Yang style Tai Chi, practiced in group once a week and practiced on their own for 10 minutes each day at least 4 days per week for 15 weeks. The TC instructor had 22 years of teaching experience. | QOL:SF-36; Physical performance: strength, flexibility; Psychological: stress; Compliance; |
| Ho RTH2012 | RCT | China | Schizophrenia | Wu-style Cheng-form Tai-chi, 1 hour per session, twice weekly for 6 weeks. Additional half-hour trainer-led practice sessions were held on a weekly basis throughout the 12-week study period, accumulating to a total of 2.5 hours of Tai-chi practice per week. | Symptom; Physical performance; |
| Wang CC2008 | RCT | USA | Knee Osteoarthritis | 24-form simplified Yang style Tai Chi, 60 minutes per session, two sessions per week, for 12 weeks. Each session includes: warm up and review of Tai Chi principles and techniques, Tai Chi exercises, breathing techniques and various relaxation methods. | QOL:SF-36; Symptom; Physical performance: strength, flexibility, BMI, balance; Psychological: depression, self-efficacy, social support, outcome expectations; Compliance; Safety |
| Madeleine E2008 | RCT | USA | Parkinson disease | 24-form simplified Yang style Tai Chi, twice weekly one hour session, completing 20 lessons within 13 weeks. | Physical performance: strength, flexibility, balance; |
| Guo XN 2010 | RCT | China | Knee Osteoarthritis | 24-form simplified Yang style Tai Chi, led by an experienced instructor, 40 minutes per session, three 8-weeks sessions with frequency gradually increased, that is, 2 days per week for the ﬁrst stage, 3 days per week the second, and 4 days per week the third. | Symptom; Physical performance: strength, flexibility; Safety |
| Li FZ2004 | RCT | USA | Falls Prevention | 24-form simplified Yang style Tai Chi, 60 minutes per session, three sessions per week, for 24 weeks. Sessions included 5–10 min of warm-up, 30 min of Tai Chi practice, and 5–10 min of cool-down. Instruction covered learning new movements and reviewing movements learned in previous sessions. Each practice session included musical accompaniment. | Incidence: falls; Physical performance: strength, flexibility, balance; |
| Yeh GY2013 | RCT | USA | Heart Failure | Yang style short form Tai Chi, 1-hour session twice weekly for 12 weeks with an experienced instructor. Each patient was encouraged to practice at home at least 3 times per week. | QOL: Minnesota Living With Heart Failure Questionnaire (MLHF); Physical performance: strength, flexibility, cardiovascular function, pulmonary function; Psychological: mood, self-efficacy; |
| Wang CC2009 | RCT | USA | Knee Osteoarthritis | 10 form of classical Yang style Tai Chi with minor modifications that were suitable for people with knee pain, 60 minutes per session, 2 sessions per week, for 12 weeks. Every session included: 10 minute self-massage and a review of Tai Chi principles; 30 minutes of Tai Chi movement; 10 minutes of breathing technique; and 10 minutes of relaxation. | QOL:SF-36; Symptom; Physical performance: strength, flexibility, balance; Psychological: depression, self-efficacy; Compliance; Safety |
| Chan AWK2011 | RCT | China | COPD | 13-form TCQ modified from the 18-form Tajii Qigong developed by Master Lin Hou Sheng in 1982, led by a qualified TC instructor, two 60-min sessions each week for 12 weeks. | Physical performance: strength, flexibility, cardiovascular function, pulmonary function; |
| Yeung A2012 | RCT | USA | depression | First section of the traditional 108 movements Yang-style, taught by a senior instructor, 60 minutes per session, twice weekly for 12 weeks. | Psychological: depression, general status, social support; Satisfaction; |
| Audette JF2006 | RCT | USA | Health promotion | 24-form simplified Yang style Tai Chi, 60 minutes per session, three times per week for 12 weeks. Each session consisted of 15–20 min of warm up and 40–45 min of TC practice. | Physical performance: strength, flexibility, cardiovascular function; |
| Li FZ2001 | RCT | USA | Health promotion | 24-form simplified Yang style Tai Chi, 60 minutes per session, for 24 weeks. Each session consisted of a 15-min warm-up, 30 min of Tai Chi, and a 15-min cool down period. | QOL: SF-20; Psychological: self-efficacy; |
| Field T2013 | RCT | USA | Depression | Tai Chi, 20 minutes per session weekly for a period of 12 weeks, led by a trained yoga instructor. | Psychological: depression, anxiety, quality of sleep; |
| Ho RTH2012 | RCT | China | Schizophrenia | Wu-style Cheng-form Tai Chi, 1 hour per session, twice weekly for 6 weeks. Additional half-hour trainer-led practice sessions were held on a weekly basis throughout the 12-week study period, accumulating to a total of 2.5 hours of Tai-chi practice per week. | Symptom; Physical performance: strength, flexibility; |
| Nedeljkovic M2012 | RCT | Switzerland | Health promotion | The first 18 sequences of the 37 Chen Man-Ch’ing Yang-Style, 60 minutes per session, twice a week, for 12 weeks. Each session began with warm-up exercises (15 min) followed by practicing Tai Chi movements and reviewing the underlying principles (35 min) and concluded with Tai Chi related breathing and relaxation exercises (10 min). | Physical performance; Psychological: depression, stress; |
| Tsai JC2003 | RCT | China | Hypertension | Yang style Tai Chi, led by a qualified master, 50 minutes per session, 3 times per week, for 12 weeks. Each session included 10-minute warm-up, 30-minute Tai Chi exercise, and 10-minute cool-down. Exercise intensity was estimated to be approximately 64% of maximal heart rate. | Physical performance; Psychological: mood, anxiety; Compliance; |
| Galantino ML 2005 | RCT | USA | HIV | Tai Chi, 60 minutes per session, twice weekly, for 8 weeks. | QOL: MOS-HIV Survey; Physical performance: strength, flexibility, cardiovascular function, balance; Psychological: mood, anxiety; |
| Liu X2012 | RCT | Australia | Type 2 diabetes | Tai Chi, a supervised group-based program with 1.5 h per session, 3 sessions per week for 12 weeks; encouraged to practice at home. | QOL:SF-36; Physical performance; |
| Wolf SL1997 | RCT | USA | Postural stability | 10 forms from the classic 108-form Yang style, 60 minutes per session, 2 sessions per week, for 15 weeks. The first meeting of the week was to acquaint the group with the form. The second meeting permitted individualized attention to practice and facilitate accurate movement technique. The movement elements contributing to each form became progressively more complex and required gradual increases in head, neck, and trunk rotation, with a simultaneous reduction in base of support. | Physical performance: balance; Psychological: fear of falling; |
| Hosseini H 2011 | RCT | Iran | Health promotion | 10-form Tai Chi developed by Wolf et al, led by two physical training coaches, for 12 weeks, 3 sessions each week. While the duration of the Tai Chi exercise was 5 minutes in the first session and 5 minutes were added in each following week so that the intervention reached to 20 to 25 minutes of Tai Chi exercise in each session at the end of the first month. | Symptom; Physical performance: BMI; |
| Sandy C2008 | RCT | USA | Dementia | Tai Chi, 60 minutes per session, 3 sessions per week, for 40 weeks. | Symptom; Physical performance: strength, balance; Psychological: depression, self-esteem; |
| Zhang JG2006 | RCT | China | Falls Prevention | 24-form simplified Yang style Tai Chi, taught by a instructor with 5 years of teaching experience, 60 minutes per session, seven times per week for 8 weeks. Each session included 10 min of warm-up exercise, 40 min of TCC practice, and 10 min of cool-down exercise. Each form of TCC emphasized multidirectional weight shifting, awareness of body alignment, and multi segmental movement coordination. Regulated breathing was emphasized as part of the exercise. Home program consisted of 11 easy forms (the 1st to 11th forms of the 24 forms used in the practice session), 30 minutes per session. The subjects were asked to practice this home program when they could not participate in a practice session or whenever they wished, and were asked to record the time they practiced TCC at home in their exercise log. | Physical performance: strength, flexibility, balance; |
| Chen EW2012 | RCT | China | Balance control | 8-form Yang style Tai Chi, 1.5 h sessions, three times per week for 16 weeks. | Physical performance: strength, flexibility, perception; |
| Wolf SL2006 | RCT | USA | Health promotion | 24-form simplified Yang style Tai Chi, two sessions per week at increasing durations starting at 60 minutes of contact time and progressing to 90 minutes over 48 weeks. In addition, participants were asked to supplement their TC training with home-based exercise, with the goal of exercising 4–5 days per week, 10–30 minutes per day. The actual work time, exclusive of warm-up and cool-down, progressed from approximately 10 to 50 minutes. | Physical performance: strength, flexibility, cardiovascular function, BMI; |
| Chris J2004 | RCT | USA | Postural stability | Eight of the 24-form simplified Yang style Tai Chi that best emphasized trunk rotation, weight shifting, coordination, and narrowing of lower extremity stance were used in each class. Twice weekly, the intensity was increased from 20 to 50 minutes in each session, for 48 weeks. | Physical performance: balance; |
| Zhou LP2010 | RCT | China | Balance control | 24-form simplified Yang style Tai Chi, taught by a professional coach, 60 minutes per session, 5 sessions per week, for 16 weeks. Each session consisted of 15 minutes of warm up, 40 minutes of practice and 5 minutes of cool down. | Physical performance: perception; |
| Chen L2007 | RCT | China | Health promotion | 24-form simplified Yang style Tai Chi, 60 minutes per session, 3 sessions per week, for 12 weeks. | Physical performance; |
| Zhu JJ 2011 | RCT | China | Osteoporosis | Tai Chi, 50 minutes per session, 3 sessions per week, for 24 weeks. | Symptom; |
| Luo D2012 | RCT | China | Health promotion | Tai Chi, 45-60 minutes per session, 5 sessions per week, for 24 weeks. | Physical performance; Psychological: general status; |
| Wang YL 2012 | RCT | China | Breast cancer | 24-form simplified Yang style Tai Chi, began at 10 days 10 days after operation, twice daily, 20 minutes per session. The intensity could be modified according to health status. | QOL: World health Organization Quality of Life Questionnaire; Symptom; Physical performance: strength, flexibility, ADL; |
| Ran B2009 | RCT | China | Health promotion | Tai Chi, led by a physical training coach, 30 minutes per session, twice daily, for 12 weeks. | Physical performance: cardiovascular function; |
| Li G2012 | RCT | China | Lumbar disc herniation | Chen style Tai Chi, led by a teacher, twice daily, 40 minutes per session, for 2 weeks. | Symptom; |
| Tang QH2009 | RCT | China | Essential Hypertension | 24-form simplified Yang style Tai Chi and 48-form Tai Chi, jogging 200 meters before practicing Ta Chi. 30-60 minutes per session, 3-5 sessions per week, for 24 weeks. | Symptom; |
| Lin HZ2012 | RCT | China | Diabetic nephropathy | 24-form simplified Yang style Tai Chi, 1.5 hour per session, once daily, for 12 weeks. | Physical performance; Safety |
| Lan YS2009 | RCT | China | Health promotion | 24-form simplified Yang style Tai Chi, 45 minutes per session, 3 sessions per week, for 6 weeks. | Physical performance: strength, cardiovascular function; Psychological: general status, concentration; |
| Song H2008 | RCT | China | Lumbar disc herniation | 24-form simplified Yang style Tai Chi, led by a professional instructor, 60 minutes per session, 6 sessions per week, for 24 weeks. | Symptom; Physical performance: perception; |
| Zhang LH2012 | RCT | China | COPD | 24-form simplified Yang style Tai Chi, 2-3 times per session, once daily, for 48 weeks. | QOL: St Georges Respiratory Questionnaire (SGRQ); Physical performance: pulmonary function; |
| Jin H 2012 | RCT | China | Psychological illnesses | 24-form simplified Yang style Tai Chi, led by two professional coaches, 60 minutes per session, twice per week, for 8 weeks. | Psychological: general status; |
| Wei D 2003 | RCT | China | Heart Failure | 24-form simplified Yang style Tai Chi, once daily. | Symptom; |
| Li C2009 | RCT | China | Insomnia | 24-form simplified Yang style Tai Chi, once daily, at least 5 sessions per week, for 12 weeks. | Symptom; |
| Jin XQ2010 | RCT | China | Health promotion | 24-form simplified Yang style Tai Chi, 60 minutes per session, 5 sessions per week, for 12 weeks. | Physical performance: strength, flexibility, cardiovascular function, pulmonary function; Psychological: depression, anxiety, general status; |
| Ding FM2013 | RCT | China | Acute myocardial Infarction | 42-form Chen style Tai Chi, 60 minutes per session, 5 sessions per week, for 24 weeks. Each session consisted of warm up (15 min), TC practice (30 min), and cool down (15 min). | Incidence: angina; QOL:SF-36; Physical performance; |
| Mao HN2006 | RCT | China | Hypertension | 24-form simplified Yang style Tai Chi, 60 minutes per session, 6 sessions per week, for 8 weeks. Each session consisted of warm up (5 min), TC practice (30 min), and cool down (10-15 min). | Symptom; Physical performance; Safety |
| Gan JL 2010 | RCT | China | Health promotion | 24-form simplified Yang style Tai Chi, 60 minutes per session 2 sessions per week, for 8 weeks. | Physical performance; |
| Li RZ2013 | RCT | China | Alcheimer's disease | 24-form simplified Yang style Tai Chi, 5 sessions per week, for 4 weeks. | Symptom; |
| Wang P2009 | RCT | China | Type 2 diabetes | Yang style Tai Chi, 5-7 sessions per week, 30-50 minutes per session, for 24 weeks. | Physical performance; |
| Li JQ2007 | RCT | China | Chronic disease | 24-form simplified Yang style Tai Chi, taught by TC master, 1.5 hour per session, once weekly, for 7 weeks. | QOL:SF-36; Psychological: confidence, self-efficacy; |
| Liu D2008 | RCT | China | Postmenopausal osteopenia | 24-form simplified Yang style Tai Chi, taught by TC master, 40-45 minutes per session, for 16 weeks. | Physical performance; |
| Li HY2012 | RCT | China | Stroke | 1st period: Imagination the movements of Tai Chi, when sitting and closing eyes; practicing Tai Chi to move limbs.10 minutes per session, 3 sessions daily. 2nd period: From bed to the ground, practiced the first movement of Tai Chi when standing, 40 minutes per session, once daily. 3rd period: Practiced the first movement of Tai Chi and moved feet; the upper limbs practiced other movements of Tai Chi. 40 minutes per session, once daily. Each period should be modified according to patients health status. | Physical performance: balance; |
| Zhang SQ2011 | RCT | China | Acute myocardial infarction | 42-form Chen style Tai Chi, 5-10 minutes per session, 5 sessions per week, for 48 weeks. | Incidence: angina, myocardial infarction or arrhythmia; QOL: Minnesota Living With Heart Failure Questionnaire (MLHF); Physical performance: cardiovascular function; |
| Yu Y 2004 | RCT | China | Type 2 diabetes | Tai Chi, 60 minutes per session, once daily, for 12 weeks. Each session consisted of 5-15 minutes of warm up, 30 minutes of TC practice and 10-15 minutes of cool down. | Symptom; |
| Pan ZJ1999 | RCT | China | Health promotion | Tai Chi, 20-30 minutes per session, once daily, for 20 weeks. | Physical performance; |
| Sha P2007 | RCT | China | Atherosclerosis | 24-form simplified Yang style Tai Chi, 1-1.5 hour per session, 5-7 sessions per week, for 72 weeks. | Physical performance; |
| Li YH 2003 | RCT | China | Health promotion | 42-form Tai Chi, for 24 weeks. The first 8 weeks, participants learnt Tai Chi, 2 hours per session; the remaining 16 weeks, participants practiced TC, 1 hour per session. | Physical performance; |
| Wang XJ2003 | RCT | China | Health promotion | 24-form simplified Yang style Tai Chi. The first 8 weeks, participants learnt Tai Chi, 2 hours per session; the remaining 16 weeks, participants practiced TC, 1 hour per session. | Physical performance; |
| Yao Y 2003 | RCT | China | Health promotion | 24-form simplified Yang style Tai Chi, 60 minutes per session, 7 sessions per week, for 16 weeks. The first 4 weeks focused on learning, and the remaining 12 weeks focused on practicing. Each session consisted of 10 minutes of warm up, 45 minutes pf practice and 5 minutes of cool down. | Physical performance: strength, BMI; |
| Shi L2012 | RCT | China | Chronic Nephritis | 24-form simplified Yang style Tai Chi, 30 minutes per session, once daily, for 12 weeks. | Physical performance: cardiovascular function, pulmonary function; |
| Xiao L 2008 | RCT | China | Lumbar muscle strain | 24-form simplified Yang style Tai Chi, 30 minutes per session, for 12 weeks. The intensity could be modified according to health status. | Symptom; Physical performance: strength; |
| Xu SS2012 | RCT | China | Hepatitis B | 24-form simplified Yang style Tai Chi, taught by a professional TC master and a senior nurse, 30 minutes per session, once daily for 12 weeks. | Physical performance: biomarker; Psychological: general status; |
| Mao HN2009 | RCT | China | Postmenopausal osteopenia | Tai Chi, 45-50 minutes per session, for 20 weeks. Each session consisted of 10 minutes of warm up, 30 minutes of TC practice and 5-10 minutes of cool down. The participants received instruction bi-weekly. | Physical performance; |
| Shen HJ2012 | RCT | China | Erectile dysfunction | 24-form simplified Yang style Tai Chi, 45-50 minutes per session, for 12 weeks. Each session consisted of 10 minutes of warm up, 30 minutes of TC practice and 5-10 minutes of cool down. The participants received instruction bi-weekly. | Symptom; Physical performance; Safety |
| Qin L 2006 | RCT | China | Postmenopausal osteopenia | 85-form Yang style Tai Chi, 45 minutes per session, 5 sessions per week, for 48 weeks. | Physical performance: strength, flexibility; |
| Sha P 2008 | RCT | China | Health promotion | Tai Chi, 45-50 minutes per session, for 16 weeks. Each session consisted of 10 minutes of warm up, 30 minutes of TC practice and 5-10 minutes of cool down. The participants received instruction bi-weekly. | Physical performance; |
| Qiu YY2008 | RCT | China | Falls Prevention | 24-form simplified Yang style Tai Chi. The first 2 weeks focused on learning, and the following 8 weeks focused on practicing. 50-55 minutes per session, once daily. Each session consisted of 5 minutes of warm up, 40 minutes of TC practice and 5-10 minutes of cool down and communication. The instructor had more than 5 years of TC teaching experience. | Physical performance: balance; |
| Zhang XA 2011 | RCT | China | Generalized anxiety disorder | 24-form simplified Yang style Tai Chi, 30-40 minutes per session, once daily, for 8 weeks. | Psychological: anxiety; |
| Jiang MY 2013 | RCT | China | Lung cancer | 24-form simplified Yang style Tai Chi, led by a nurse when the participants in hospitalization, twice daily, 30 minutes per session, for 4 weeks. Participants were required to practiced at home followed the VCD of TC after they left hospital. | Symptom; |
| Chen FZ 2013 | RCT | China | Hypertension | 24-form simplified Yang style Tai Chi, taught by a professional instructor, 30 minutes per session, 6 sessions per week, for 12 weeks. | Symptom; |
| Zhou Y 2002 | RCT | China | Ankle fracture | Tai Chi, for 8 weeks. The intensity were modified according to the health status. | Incidence: fracture; Symptom; |
| Liao GX 2012 | RCT | China | Depression | 24-form simplified Yang style Tai Chi, 60 minutes per session, 5 sessions per week, for 24 weeks. Each session consisted of 10 minutes of warm up, 40 minutes of practice and 10 minutes of cool down. | QOL:SF-36; Psychological: depression; |
| Wang XJ 2003 b | RCT | China | Health promotion | 24-form simplified Yang style Tai Chi, for 24 weeks. In the first 8 weeks, participants learnt TC, 1.5 hour per session, once daily. In the following 16 weeks, the participants practiced TC, 60 minutes per session. | Physical performance: biomarker; |
| Zeng YH 2009 | RCT | China | Balance control | 24-form simplified Yang style Tai Chi, 40-60 minutes per session, 3 sessions per week, for 96 weeks. | Incidence: falls and fracture; Physical performance: strength, balance; |
| Yao YP 2004 | RCT | China | COPD | Tai Chi, taught by a professional instructor, 30 minutes per session, once daily, for 12 weeks. | Symptom; Physical performance: pulmonary function; |
| Wang L 2012 | RCT | China | Acute myocardial infarction | 24-form simplified Yang style Tai Chi, 2 sessions per week, for 4 weeks. | Psychological: depression; |
| Zhou QA 2010 | RCT | China | Acute myocardial infarction | 24-form simplified Yang style Tai Chi, 2 sessions per week, for 4 weeks. | QOL:SF-36; Physical performance: balance; Psychological: anxiety; |
| Zhao SY 2005 | RCT | China | Health promotion | 24-form simplified Yang style Tai Chi and 85-form traditional Tai Chi, 40-80 minutes per session. | Psychological: general status; |
| Sha LW 2009 | RCT | China | Health promotion | Tai Chi, taught by a Wushu teacher, 7 sessions per week, for 8 weeks. A total of 300 minutes per week was required. | Psychological: general status; |
| Zhu Y 2011 | RCT | China | Parkinson disease | 24-form simplified Yang style Tai Chi, taught by a first grade sportsman, twice daily, 30-45 minutes per session, 5 days per week, for 4 weeks. | Physical performance: balance; |
| Zhan HN 2010 | RCT | China | Low Back Pain | 24-form simplified Yang style Tai Chi, taught by a professional instructor, 30 minutes per session, 3-4 sessions per week, for 24 weeks. | Symptom; |
| Tian HQ2004 | RCT | China | Low Back Pain | Tai Chi, twice daily, 2-3 times per session, for 12 weeks. | Symptom; Physical performance; |
| Liu TJ2009 | RCT | China | Stroke | 24-form simplified Yang style Tai Chi, 30 minutes per session, for 12 weeks. Nurses could stand beside the patients to help keep balance. | Physical performance: balance; |
| Zhang WJ 2011 | RCT | China | Health promotion | 24-form simplified Yang style Tai Chi, 30 minutes per session, 3 sessions per week. Each session consisted of 5 minutes of warm up, 20 minutes of TC practice and 5 minutes of cool down. | QOL:SF-36; |
| Sun W2009 | RCT | China | Balance control | 24-form simplified Yang style Tai Chi, 60 minutes per session, for 16 weeks. Each session consisted of 10 minutes of warm up, 40-45 minutes of practice and 5 minutes of cool down. | Physical performance: balance; |
| Liu D2010 | RCT | China | Health promotion | 24-form simplified Yang style Tai Chi, taught by a professional instructor, 60 minutes per session, 5 sessions per week, for 16 weeks. Each session consisted of warm up (10 minutes), TC practice (40-45 minutes) and cool down (5 minutes). | Physical performance; |
| Chen R 2011 | RCT | China | Health promotion | 24-form simplified Yang style Tai Chi, taught by a professional instructor, 60 minutes per session, for 20 weeks. | Physical performance; |
| Han YT 2009 | RCT | China | Health promotion | Tai Chi, 60 minuses per session, 3 sessions per week, for 16 weeks. | Physical performance: BMI; |
| Gong NN 2010 | RCT | China | Health promotion | 24-form simplified Yang style Tai Chi, taught by a professional instructor, 60 minutes per session, for 16 weeks. Each session consisted of warm up (10 minutes), TC practice (40-45 minutes) and cool down (10 minutes). | Physical performance; |
| Jia Q 2010 | RCT | China | Type 2 diabetes | 48-form Tai Chi, started with 30 minutes per session, 3 sessions per week, and increased gradually to 40-50 minutes per session, 4-5 sessions per week, for a total of 16 weeks. | Physical performance; |
| Xi JP 2004 | RCT | China | Insomnia | Tai Chi, 20-25 minutes per session, 1-2 sessions daily, for 8 weeks. | Symptom; |
| Li Q 2012 | RCT | China | COPD | 24-form simplified Yang style Tai Chi, 60 minutes per session, once daily, for 24 weeks. Before each session, the participants practiced Tai Chi breathing methods and then TC practice. Each session consisted of warm up (10 minutes), TC practice (40 minutes) and cool down (10 minutes). | QOL: St Georges Respiratory Questionnaire (SGRQ); Physical performance: pulmonary function; Psychological: general status; |
| Luo H 2006 | RCT | China | Essential Hypertension | 24-form simplified Yang style Tai Chi, once daily, 45 minutes per session, for 24 weeks. The intensity could be modified according to health status. | Symptom; Physical performance; |
| Lu H 2007 | RCT | China | Rheumatoid arthritis | 24-form simplified Yang style Tai Chi, once daily, 45 minutes per session, for 12 weeks. | Symptom; |
| Gan JL 2007 | RCT | China | schizophrenia | 24-form simplified Yang style Tai Chi, taught by a professional coach and a trained nurse, 60 minutes per session, 5 sessions per week, for 12 weeks. The first 2 weeks focused on movements learning, and from the 3rd week participants practiced the complete routine of TC. | Symptom; Psychological: general status; |
| Gan JL 2010 | RCT | China | schizophrenia | 24-form simplified Yang style Tai Chi, taught by a professional coach, 60 minutes per session, 5 sessions per week, for 12 weeks. The first 2 weeks focused on movements learning, and from the 3rd week participants practiced the complete routine of TC. | Physical performance: perception; |
| Zhang JH 2004 | RCT | China | Health promotion | Tai Chi, 4 sessions per week, 30 minutes per session, for 8 weeks. | Symptom; Physical performance; |
| Wei JJ 2006 | RCT | China | Health promotion | 24-form simplified Yang style Tai Chi, 40 minutes per session, 5 sessions per week, for 8 weeks. Each session consisted of 5 minutes of warm up, 30 minutes of practice and 5 minutes of cool down. | Symptom; |
| Zhang XL 2007 | RCT | China | Health promotion | 24-form simplified Yang style Tai Chi, 60 minutes per session, 5 sessions per week, for 16 weeks. Each session consisted of 10 minutes of warm up, 40-45 minutes of TC practice and 5 minutes of cool down. | Physical performance: pulmonary function; |
| Mao Y 2007 | RCT | China | Health promotion | 24-form simplified Yang style Tai Chi, 45-60 minutes per session, 3 sessions per week, for 48 weeks. | Physical performance; |
| Ma XW 2006 | RCT | China | Health promotion | 24-form simplified Yang style Tai Chi, 40 minutes per session, 5 sessions per week, for 48 weeks. | Physical performance; |
| Li CL 2008 | RCT | China | Irritable bowel syndrome(IBS) | 24-form simplified Yang style Tai Chi, taught by a professional coach, 60 minutes per session, for 8 weeks. | Symptom; Psychological: depression, anxiety; |
| Yan W 20006 | RCT | China | Hyperglycemia | Tai Chi, taught by a professional coach, started with 10 minutes per session and increased gradually to 30-40 minutes per session, 5-7 sessions per week, for 48 weeks. | Physical performance; |
| Zhou SW 2007 | RCT | China | Hypertension | 24-form simplified Yang style Tai Chi, 60 minutes per session, once daily, for 12 weeks. | Symptom; Physical performance; |
| Bi Y 2005 | RCT | China | Hypertension | 24-form simplified Yang style Tai Chi, 45 minutes per session, once daily, for 24 weeks. | Physical performance; |
| Chen JF 2013 | RCT | China | Coronary artery disease | 24-form simplified Yang style Tai Chi, taught by a professional instructor, 60 minutes per session, 4 sessions per week, for 12 weeks. | Physical performance; |
| Yao CD 2010 | RCT | China | Chronic Heart Failure | 42-form Chen style Tai Chi, taught by a professional TC instructor, started from 5-10 minutes per session and increased gradually to 30 minutes per session, 5 sessions per week, for 24 weeks. | QOL: Minnesota Living With Heart Failure Questionnaire (MLHF); Symptom; Physical performance: cardiovascular function; |
| Du ST 2013 | RCT | China | COPD | 24-form simplified Yang style Tai Chi, 60 minutes per session, or twice daily with 30 minutes per session. In addition, 2 group sessions per week, 60 minutes per session, taught by a professional instructor, for 12 weeks. | Physical performance: strength, pulmonary function; |
| Wang P 2009 | RCT | China | Type 2 diabetes | 24-form simplified Yang style Tai Chi, 45-60 minutes per session, 5-7 sessions per week, for 24 weeks. | QOL:SF-36; |
| Li GP2011 | RCT | China | Health promotion | 24-form simplified Yang style Tai Chi, 60 minutes per session, 5 sessions per week, for 24 weeks. Each session consisted of 10 minutes of warm up, 40 minutes of TC practice and 10 minutes of cool down. | Aging; |
| Wang WQ 2010 | RCT | China | Postmenopausal osteopenia | 24-form simplified Yang style Tai Chi, taught by a professional instructor, 45-60 minutes per session, for 12 weeks. | QOL:SF-36; Physical performance; |
| Sun QQ 2010 | RCT | China | Hypertension | 24-form simplified Yang style Tai Chi, taught by a professional instructor, 6 sessions per week, for 12 weeks. | QOL:SF-36; Symptom; |
| Luo BB 2012 | RCT | China | Balance control | 24-form simplified Yang style Tai Chi and 42-form Tai Chi, 60 minutes per session, 3 sessions per week, for 32 weeks. | Physical performance: balance; |
| Han QY 2010 | RCT | China | Essential Hypertension | 24-form simplified Yang style Tai Chi, taught by a professional TC instructor, 45-60 minutes per session, 1-2 sessions per week, for 12 weeks. | Incidence: heart failure, cerebral hemorrhage or renal function damage; QOL:SF-36; Symptom; |
| Sun MJ 2005 | RCT | China | Health promotion | Tai Chi, taught by a professional instructor, 2 hours per session, 4 sessions per week, for 24 weeks. | Physical performance: strength, flexibility, pulmonary function, balance; |
| Duan YM 2012 | RCT | China | Frozen shoulder | 24-form simplified Yang style Tai Chi, 50 minutes per session, 5 sessions per week, for 8 weeks. | Symptom; |
| Wang XJ 2011 | RCT | China | Hypertension | Tai Chi, 60 minutes per session, 5 sessions per week, for 16 weeks. | Symptom; |
| Song XH 2011 | RCT | China | Chronic disease | Yang style or Chen style Tai Chi, 0.6-1.5 hour per session, 3-5 sessions per week. | Symptom; |
| Wang YG 2002 | RCT | China | Type 2 diabetes | 24-form simplified Yang style Tai Chi, 15-60 minutes per session, once daily. | Symptom; |
| Xie Y 2008 | RCT | China | schizophrenia | 24-form simplified Yang style Tai Chi, 45 minutes per session, for 12 weeks. | Symptom; Physical performance; |
| Zhang WY 2011 | RCT | China | Osteoporosis | Tai Chi, led by a professional instructor, 30~60 minutes per session, 3-4 sessions per week, for 24 weeks. | Physical performance; |
| Xiao L 2010 | RCT | China | Type 2 diabetes | 24-form simplified Yang style Tai Chi, 60 minutes per session, 6 sessions per week, for 24 weeks. | Physical performance; |
| Xie YS 2011 | RCT | China | Irritable bowel syndrome(IBS) | 24-form simplified Yang style Tai Chi and 42-form Tai Chi, 60 minutes per session, twice daily, for 12 weeks. | Symptom; |
| Yang L 2009 | RCT | China | schizophrenia | 24-form simplified Yang style Tai Chi, 45 minutes per session, 3 sessions per week. | Symptom; |
| Ling JH 2012 | RCT | China | Diabetic nephropathy | 24-form simplified Yang style Tai Chi, 1.5 hour per session, once daily, for 12 weeks. | Symptom; Physical performance; Safety |
| Liu CZ 2010 | RCT | China | Chronic Fatigue Syndrome | 24-form simplified Yang style Tai Chi, led by a professional instructor, 30 minutes per session, once daily, for 4 weeks. | Physical performance; Safety |
| Liu C 2003 | RCT | China | Lumbar muscle strain | 24-form simplified Yang style Tai Chi, twice daily, for 4 weeks. | Symptom; |
| Ye D 2012 | RCT | China | Insomnia | 37-form Yang style Tai Chi, led by a professional instructor, 40 minutes per session, 2-3 sessions per week, for 8 weeks. | Symptom; |
| Li J 2007 | RCT | China | Carpal bone fracture | 24-form simplified Yang style Tai Chi, 15-30 minutes per session. | Symptom; |
| Liang YS 2010 | RCT | China | Irritable bowel syndrome(IBS) | 24-form simplified Yang style Tai Chi and 42-form Tai Chi, led by a professional instructor, twice daily, 30 minutes per session, for 12 weeks. | Symptom; |
| Liang YS 2010a | RCT | China | Irritable bowel syndrome(IBS) | 24-form simplified Yang style Tai Chi and 42-from Tai Chi, 60 minutes per session, twice daily, for 8 weeks. | Symptom; |
| Zhang TM 2006 | RCT | China | Health promotion | Yang style Tai Chi, 60 minutes per session, 3-4 sessions per week, for 24 weeks. | Physical performance: cardiovascular function, BMI; |
| Wang CY 2006 | RCT | China | Health promotion | 24-form simplified Yang style Tai Chi, 3 sessions per week, for 12 weeks. One session was a 90-minute class, and the other two sessions was 50-minutes practice in the morning. | QOL:SF-20; Psychological: general status; |
| He JH 2011 | RCT | China | Breast cancer | 24-form simplified Yang style Tai Chi, 40-50 minutes per session, 3 sessions per week, for 16 weeks. | QOL:FACT-B,HRQL; Physical performance: strength, pulmonary function; |
| Chen GL 2007 | RCT | China | Health promotion | 24-form simplified Yang style Tai Chi, twice per week, for 8 weeks. | Symptom; |
| Tian HQ2007 | RCT | China | Frozen shoulder | 24-form simplified Yang style Tai Chi. The intensity and time could be modified according to health status. In acute phase: 10-15 minutes per session, 3-4 sessions per day. In severe phase: 10-15 minutes per session, 4-5 sessions per day. In recover phase: 15-20 minutes per session, 3-4 sessions per day. | Symptom; |
| Yu Y 2006 | RCT | China | Type 2 diabetes | Tai Chi, 60 minutes per session, once daily, for 12 weeks. | Symptom; Physical performance: BMI; Psychological: general status; |
| Yang L 2008 | RCT | China | schizophrenia | 24-form simplified Yang style Tai Chi, 45 minutes per session, 3 sessions per week, for 12 weeks. | Symptom; |
| Guo HH 2012 | RCT | China | COPD | 24-form simplified Yang style Tai Chi, 3 sessions per week, for 12 weeks. | Physical performance; |
| Ni J 2010 | RCT | China | Coronary artery disease | 24-form simplified Yang style Tai Chi, 30-60 minutes per session, 2-3 sessions per week, for 24 weeks. | Physical performance; |
| Zhang DY 2011 | RCT | China | Low Back Pain | Tai Chi, 20 minutes per session, 1-2 sessions daily. | Symptom; |
| Zhou Y 2003 | RCT | China | Ankle fracture | Tai Chi, started from 10-20 minutes per session, and increased gradually to 40 minutes per session, for a total of 12 weeks. | Symptom; |
| Liu HL 2005 | RCT | China | Health promotion | 24-form simplified Yang style Tai Chi and 42-form Tai Chi, 30 minutes per session, once daily, for 24 weeks. | QOL:SF-36; |
| Tang L 2012 | RCT | China | Health promotion | 24-form simplified Yang style Tai Chi and 48-form Tai Chi, 30 minutes per session. | Symptom; |
| Zhang DY 2011 | RCT | China | Prostatitis | Tai Chi, 20-40 minutes per session, once daily, for 4 weeks. | Incidence: chronic nonbacterial prostatitis; Symptom; |
| Liu CZ 2010a | RCT | China | Chronic Fatigue Syndrome | 24-form simplified Yang style Tai Chi, led by a professional instructor, 30 minutes per session, once daily, for 4 weeks. | Symptom; Physical performance; Safety |
| Zhang DD 2009 | RCT | China | Coronary artery disease | 24-form simplified Yang style Tai Chi and 48-form Tai Chi, 40 minutes per session, twice daily, 6 sessions per week, for 12 weeks. | Incidence: angina; |
| Sun XY 2012 | RCT | China | Breast cancer | 24-form simplified Yang style Tai Chi, twice daily, 20 minutes per session. | QOL: World Health Organization quality of life questionnaire; Symptom; Physical performance: strength, flexibility, ADL; |
| Gan JL 2011 | RCT | China | Health promotion | 24-form simplified Yang style Tai Chi, 30 minutes per session, twice daily, for 6 weeks. Each session consisted of warm up (5 min), TC practice (20 min) and cool down (5 min) | Physical performance; Psychological: general status; |
| Harling A2008 | SR | UK | Falls Prevention | Modified TC, Classical Yang style, 10 movements of 108 forms Yang style TC, 6 movements of the 24 simplified forms Yang style TC, 24-form simplified Yang style TC. Average: 3 sessions per week (range one to seven); mean total intervention contact time was 54 hours (range 27–96 hours). 1 hour per week for 37 weeks; 3 sessions per week for 12 weeks; 1 hour per session, 3 sessions per week for 26 weeks; 3 sessions per week for 104 weeks; 2 sessions per week for 15 weeks; 60–90 min per session, 2 sessions per week for 48 weeks; 1 hour per session, 7 sessions per week for 8 weeks. | Incidence: falls; Psychological: fear of falling; |
| Wayne PM 2004 | SR | USA | Balance control | 10 movements of Yang style TC; 108-form Yang style TC; 9 movements of Yang style TC; 24-form simplified Yang style TC; Tai Chi–like dance program; Tai Chi-like movements; 20 form TC; 37-form Yang style TC; Tai Chi: Combination of Yang, Wu, and Pa Kua styles. Three 1-2 hour per session, once weekly for 12 week; two 1-hour sessions per week for 12 week; 2 times per week for 24 week; ten 2-hour sessions, 1 times weekly education; 3 sessions per week for 6 months; 90 min per week for 8 week; one 1-hour session weekly; 45 minutes per session, 3 times per week for 8 weeks; daily practice, 12 months; 1 hour per week for 10 weeks; daily practice, 6 months; 3 times per week for 16 weeks; 1 time per week for 8 weeks, or not available. | Incidence: falls; Physical performance: strength, flexibility, balance; Psychological: fear of falling; |
| Lee MS2008 | SR | UK | Parkinson disease | Tai Chi. 45-80 min, 2-5 times weekly for 8-16 weeks; or 90 min, 5 consecutive days. | Incidence: falls; QOL:SF-36; Physical performance: strength, flexibility, balance; Psychological: depression; |
| Lee MS2007 | SR | UK | Breast cancer | Tai Chi. 50-60 min, 3 times weekly for 6-12 weeks. | QOL:SF-36; Symptom; Physical performance: strength, flexibility, ADL; Psychological: depression, self-esteem; Safety |
| Liu H2010 | SR | USA | Osteopenia, Osteoporosis, Diabetes | Yang's style, 5-24 form, or not abailable; Sun, 12 forms; Chen, 13 forms; Wu (Ng), 108 forms; Mix of Yang and Sun, no standardized forms mentioned; or not available. In summary, most of the TC protocols required a duration of 12 weeks or more, with a frequency of at least 2 times a week and minimum of 45 minutes of TC session time. | Incidence: falls; Physical performance: strength, flexibility, balance; Psychological: fear of falling; |
| Mansky P2013 | SR | USA | Breast cancer, Rheumatoid arthritis, Vestibulopathy, Depression, Chronic heart failure, Hypertension, Chickenpox, Osteoarthritis, Myocardial infarction, HIV | Tai Chi. 1-hour classes, 3 times per week for 6 weeks; 60-min session, 3 times per week for 6 months; 3 (~60 min) times per week for 6 months; 2 times per week, increasing from 60 to 90 min over 49 weeks; 1 h per session; 3 times per week for 12 months; 3 times per week for 50 min per session for 12 weeks; 1 hour per session, 2 times per week for 3 weeks, then weekly for 5 weeks; or NA | QOL:SF-36;Symptom; Physical performance: cardiovascular function; Psychological: depression, stress, general status; |
| Yeh GY2009 | SR | USA | Cardiovascular disease | Wu style TC/Qigong, Yang style TC (Simplified 24, forms), Wu Chian Chuan style, Modified TC, TC/Qigong (15 style), Cheng 119 style TC, TC (unspecified style), Cheng 119 style TC, TC ( Simplified 24, 40, and 48 forms), TC (Simplified form), TC/Qigong (54 movements), Yang style TC (13 movements), Yang style TC (108 postures), TC/Qigong (18 postures; unspecified duration), Yang and Sun style TC (12 movements) Paul Lam’s Tai Chi for Diabetes Program, Yang style TC (24 posture short form), Cheng TC 37 forms. | Symptom; Physical performance: cardiovascular function; |
| Ng SM2012 | SR | China | Chronic heart failure, Coronary heart disease | Yang-style TC short form, Wu-style TC, 24 forms simplified Yang style TC, Short-form Wu-style TC, Combined form of Yang-style and Sun style TC, Classical Yang-style. 1/wk; 1 h, 2/wk; 1/d; 30 min, 2/wk for 3 wk, then 1/wk for 5 wk; 60 min, 1/wk. | QOL:SF-36; Symptom; Physical performance: cardiovascular function; Psychological: mood; |
| Lee MS2008a | SR | UK | Osteoarthritis | Tai Chi. 60 min per session, 2-3 times weekly for 12 weeks; 40 min per session, 3 times weekly for 6 weeks plus home based tai chi for 6 weeks; 60 min per session, once weekly for 10 weeks; 50 min per session, 3 times weekly for 4 weeks and 5 times weekly for 14 weeks; 50 min per session, 3 times weekly for 6 months; 60 min per session, 1-2 times weekly for 8 weeks; 60 min per session, 2 times weekly for 6 weeks; 120 min per session, 2 times weekly for 6 weeks. | QOL:SF-12; Symptom; Physical performance: flexibility, ADL, balance; |
| Lee MS2007 | SR | UK | Rheumatoid arthritis | Tai Chi. 60 min per session, 1-2 weekly for 6-12 weeks; 50 min per session, once weekly for 12 weeks | QOL:SF-36; Symptom; Physical performance: strength, flexibility, ADL; Psychological: depression, mood; |
| Ding M2012 | SR | China | Stroke | Short-form Tai Chi, or not available. 1 hr weekly for 12 wks, plus approximately 3 hrs of self-practice per week; 1hr twice weekly; A 60-min Tai Chi class three times a week for 12 wks; 50-min Tai Chi sessions conducted once a week for 12 wks; Twice weekly for 4 wks. (The Tai Chi exercise varied from 50 to 240 minutes, one to three times per week, for 4-12 wks.) | QOL; Physical performance: strength, flexibility, balance; Psychological: depression, anxiety, quality og sleep; |
| Yeh GY2008 | SR | USA | Hypertension, Coronary heart disease, Cardiovascular diseases, chronic rheumatologic and dermatologic conditions | Yang-style TC (13 movements, 108 postures, 18 postures, simplified 24 forms, 24 forms, 24-posture short form, unspecified style; simplified 24 forms, 108 movements; 42 styles; or NA ); Wu-style TC | Physical performance: cardiovascular function; |
| Wang CC2004 | SR | USA | Chronic disease | 108 forms of Yang style TC; Selected from Yang, Pa-Kua, and Wu; Simplified form; 24 form Simplified ; Beijing 24 forms; Classical Yang; 9 forms from Yang; A series of 15 movements extracted from the Yang style; Wu style short form; Long form of Yang; Either the long form or Yang style or Wu style; or not available. 8-16 weeks (1 h, 2 times/wk);12 weeks (3 times/wk); 8 weeks (1 h/wk); 16 weeks (50 min, 3 times/wk); 10 weeks (1 h/wk); 8 weeks (45 min, 3 times/wk); 6 months (1 h/wk); 1-35y; 10 weeks (90 min, 1 time/week in study 1), (90 min, 2 times/wk in study 2); 6 months (54 min/d); 8 weeks (1 h, 2 times/wk for 3 week and 1 time/wk for 5 wk); 3 y (every morning); 12 week (40 min, 2 times/wk); 6.3-6.7 y (54 min, 4 times/wk); 11.8 y (4.3 times/wk); 12 mo (54 min, 4.6 times/wk); 1 y (54 min, 3.8 times/wk); 18 months (30 min, 6 times/wk); 90 days (40-60min, 2 times/d; or 3 times/wk); 6 months (1 h, 2 times/wk); 16 week (45 min, 3 times/wk); 12 week (1 h, 2 times/wk); 1 months (30 min, 6 times/wk); 7 week (2 times/wk); 10-70 years (40 min/d); 6 months (1 h, 2 times/wk); 11.2 years (54 min, 5 times/wk); 20 days (5 min/d before bed). | Incidence: falls; QOL; Symptom; Physical performance: strength, flexibility, cardiovascular function, pulmonary function, biomarker, balance; Psychological: depression, stress, mood, anxiety, general status, self-efficacy; |
| Wang WC2009 | SR | Australia | Moderate heart failure, Hip osteoarthritis, Knee osteoarthritis, HIV/AIDS, Depression, Hypertension, Back pain | Mainly a modified Yang style (that to be performed slowly with a slow breathing technique). The intensity of TC varied between once and three times, 40 to 90 minutes per week for 6 to 48 weeks. Home practice was required only in some studies but was not monitored. | Psychological: depression, stress, mood, anxiety, general status, self-efficacy; |
| Sjosten N2008 | SR | Finland | Falls Prevention | Land-based Tai Chi. Twice a week in groups at increasing difficulty and unsupervised home- exercises advised to perform at least 15 minutes a day; 3 times a week for 1 hour; twice a week increasing progressively from 60 to 90 minutes. | Psychological: depression, fear of falling; |
| Maciaszek J2010 | SR | Poland | Falls Prevention | Tai Chi, 1-7 times a week, or not available. | Physical performance: strength, flexibility, balance; |
| Wayne PM 2007 | SR | UK | Falls Prevention | Long-term Tai Chi, Tai Chi solo form, Tai Chi push hands. 5 sessions per week, 45 min per session; or not available. | Physical performance; |
| Verhagen VP2004 | SR | Netherlands | Falls Prevention | Modified Yang style, varying from 10 to 24 forms. The intensity of TCC varies from 1 h weekly for 10 weeks to 1 h every morning for 1 year. (twice weekly for 15 weeks; every morning, 1 h for 1 year; 1 h, twice a week for 6 months; 1 h weekly for 10 weeks; three times per week, 45 min for 8 weeks; 4 days per week, 30 min, for 12 weeks). | Physical performance: strength, flexibility, cardiovascular function, balance; |
| Dalusung-Angosta A2011 | SR | USA | Coronary artery disease | Classic Yang form of Tai Chi that consists of 108 movements, short form of Tai Chi consisting of 24 movements. At least 60-minute per session, included warm-up and cool-down periods. Two, three, or five times per week. | Physical performance: cardiovascular function; |
| Serena L2009 | SR+meta | Singapore | Falls Prevention | Sun-style Tai Chi, 24-form Yang style, or not available. | Incidence: falls; Physical performance: strength, flexibility, balance; Psychological: fear of falling; |
| Yan JH2013 | SR+meta | China | COPD | 13-form Tai Chi Qigong, Yang-style short form, short-form Sun-style TC, 24-form simplified Yang style TC, Chen- style short form. 12 weeks ×2 times per week; 60 min per time; 24 weeks× 1 time per day; 60 min per time; 12 weeks ×1 time per day, 30 min per time; 48 weeks ×1 time per day, 30–60 minutes per time; 16 weeks ×5 times per day, 40 minutes per time. | QOL: Chronic Respiratory Disease Questionnaire, St George's RQ; Symptom; |
| Liu B2011 | SR+meta | China | Falls Prevention | Tai Chi. 1 hour per session, 3 sessions per week; 20 weeks; 50 minutes per session, 3 sessions per week for 12 weeks; 1 hour per session, (4.6±1.3) per week for (11.2±1.4) months; 1 hour per session, 7 sessions per week, for 16 weeks. | Physical performance: strength, flexibility, cardiovascular function; |
| Lei P2013 | SR+meta | China | Chronic Heart Failure | Wu Chian Chuan style, Chi Kung exercises (the Orchid Hand 21 Style and Wu’s Chi Kung), Yang style and cycling or walking, Master Cheng Man-Ching’s Yang-style short form. 50–60 min per session; twice weekly, or three times per week. | QOL: Minnesota Living with Heart Failure Questionnaire; Physical performance: cardiovascular function; |
| Brosseau L2004 | SR+meta | Canada | Osteoarthritis | Tai Chi, two 1-hour Tai Chi classes per week. | Symptom; Physical performance: strength, flexibility, pulmonary function; |
| Yan JH2013 | SR+meta | China | Osteoarthritis | Tai Chi, 40-60 minutes per session, once weekly, or three times weekly for 6 weeks plus home based Tai Chi Tai Chi for 6 weeks, or twice a week, twice weekly for the first 3 weeks and once weekly for the next weeks. | Symptom; Physical performance: strength, flexibility; |
| Rand D2011 | SR+meta | Israel | Balance control | Tai Chi, 10 weeks (2 × week), 26 weeks (3 × week for 1 hour), 48 weeks (2 × week), 8 weeks (7 × week for 1 hour), 13 weeks (2 × week for 1 hour). | Incidence: falls; Physical performance: strength, flexibility, balance; |
| Gillespie LD2003 | SR+meta | UK | Falls Prevention | Tai Chi, group sessions twice weekly, for 15 weeks. (Individual contact with instructor approximately 45 minutes per week.) | Incidence: falls; |
| Lee MS2009 | SR+meta | South Korea | Hypertension, Knee osteoarthritis, Chronic heart failure, breast cancer | Yang style (5-15 movements); Sun style (12 movements); or not available. The number of tai chi sessions ranged from 24 to about 48; The number of supervised interventions ranged from two to three sessions weekly. The session duration ranged from 60 min to 90 min per session. | Physical performance: pulmonary function; |
| Leung DP2011 | SR+meta | China | Osteoporosis, Osteoarthritis, Stroke | Yang styles (9, 10, 12, or 24 forms), Sun styles, a combination of two or more different styles, modified tai chi movements, or not available. 20 minutes to 1.5 hour per session, 1-2 sessions per week, for 7 weeks. | Incidence: falls; Physical performance: strength, flexibility, balance; |
| Lee MS2010 | SR+meta | South Korea | Breast cancer | Yang style of tai chi, combined style of Sun and Yang, or not available. The number of tai chi sessions ranged from 10 to 36. The number of supervised interventions ranged from one to three sessions weekly. The session duration ranged from 15 to 60 min per session. | QOL:FACT-B,HRQL; Symptom; Physical performance: strength, flexibility; Psychological: self-esteem; Safety |
| Lee MS2007 | SR+meta | UK | Cardiovascular disease | NA | Physical performance: cardiovascular function; |
| Lee MS2010 | SR+meta | South Korea | Hypertension | Home-based tai chi exercise, Sun and Yang style (12 forms), Simplified Yang style (24 forms), Simplified Yang style (six forms of 24 forms). (All of the included trials adopted a modified Yang style Tai Chi). 1 hour, twice weekly for 6-48 weeks; 10-50 minutes four to five times weekly or twice daily. (The average session frequency was two or three times a week, except one trial in which participants undertook tai chi four or five times a week. The duration of sessions for all included trials was 1 hour per session. Three RCTs employed an additional home-based tai chi program). | Physical performance: cardiovascular function; |
| Lee MS2011 | SR+meta | South Korea | Type 2 diabetes | 12-movement (Sun and Yang style), 20-movement (Sun and Yang style), or not available. The number of tai chi sessions ranged from 20 to approximately 168. The number of supervised interventions ranged from 2 to 7 sessions weekly, with a duration of 30-60 min per session. | Physical performance; |
| Lee MS2008b | SR+meta | South Korea | Osteoarthritis | Tai Chi, 45 minutes per session, 5 times weekly for 12 months; (45–60min, 5–7 times weekly for 6-10 months; (60 min twice weekly for 16 weeks; 40 min, 3 times weekly for 24 weeks; or NA. (The number of tai chi sessions ranged from 32 to about 280. The number of supervised interventions ranged from 2 to 7 sessions weekly. Duration ranged from 40 min to 60 min per session.) | Physical performance; |
| Kang JW2011 | SR+meta | South Korea | Osteoarthritis | Yang-style(simplified 24 forms OR 10 forms OR 9 forms), Sun-style (12 forms OR modified 24 forms), Wu-style(16 forms), or not available. 30 min, 2e4 times weekly for 24 weeks; 40 min, 3 times weekly for 6 weeks plus home-based Tai Chi for 6 weeks; 60 min, twice weekly for 12 weeks; 60 min, twice weekly for 8 weeks; 60 min, 3 times weekly for 12 weeks; 60 min, twice weekly for the first 3 weeks and once weekly for the next 6 months; 60 min, twice weekly for 12 weeks; 60 min, once weekly for 10 weeks. | QOL:SF-12; Symptom; |
| Han A2004 | SR+meta | Canada | Rheumatoid arthritis | Yang Style Tai Chi Chian Short Form, or not available. Every morning for one hour; 1 hour session 1-2 a week; a 15-20 minute warm-up period, followed by a 5 minute rest period, then was devoted to a repetition of a series of 15 movements derived from TC; or not available. | Incidence: tender joints or swollen joints; Symptom; Physical performance: strength, flexibility; |
| Wang CC2010 | SR+meta | USA | Fibromyalgia, dementia or Alzheimer's disease, Knee Osteoarthritis, Rheumatoid Arthritis, Autoimmune Deficiency Syndrome, Multiple Sclerosis, Attention Deficit Hyperactive Disorder, HIV, Breast cancer, depression, Sedentary obese | Classical Yang style, Yang style, short form of TC with 8 movements, modified 24 forms Sun style, unspecified style, 6 of the 24 simplified TC forms, 18 form of Yang style, 15 short form Yang style, 108 postures Yang style, 10 modified forms Yang style, long form, Yang style or Wu variation of Yang style, 8 movements short form; 24-posture short form, Yang style; simplified 24 forms Yang style & 42 forms TC sword, traditional segments of Yang style & MBSR, simplified 24 forms, Long Form Yang style or Wu variation of Yang style, 24, 28, 58, 88 forms of Chen, Wu, Yang style & TC sword, regular TC & TC sword, or not available. 1 hour per session, 2 sessions per week; 2 hour per session, once weekly; 90 min per session, once weekly; 40 minutes per session, 3 sessions per week; 60-90 min progression, 2 sessions per week; 45 min per session, 3 sessions per week; 1 hour per session, 3 sessions per week; 50 min per session, 3 sessions per week; 2 hours per session, once weekly for 10 sessions; 1 hour per session; 1 hour per session, 3 sessions per week; 90 min per session, once weekly for 12 sessions; 1 hour per session, once weekly; 1 hour per session, 4 sessions per week; 30 min per session, 2 sessions per week; 6 individual sessions; 2 sessions per week; 30 min per session, 6 sessions per week; 1 hour per session; Over 2 hours per day; >30 min, >3 times per week; at least 20 min per session, 3 times per week; at least 2 times per week & 30 min per session; or not available. | Psychological: depression, stress, mood, self-esteem, anxiety; |
| Taylor-Piliae RE2004 | SR+meta | USA | Heart disease | Yang style, 108 postures; Yang style, 13 movements; or not available. | Physical performance: cardiovascular function, pulmonary function; |
| Hall A2009 | SR+meta | Australia | Chronic arthritis, Chronic tension-type headache | Yang style, Sun style, Wu style; (50% of trials using a 12-week Tai Chi program comprising 18–24 sessions). Sessions lasted 40–60 minutes; 1 time/2 weeks;1-3 time/1 weeks. | QOL:SF-36; Symptom; Physical performance: strength, flexibility; Compliance; |
| Logghe IH2010 | SR+meta | Netherlands | Falls Prevention | Tai Chi. The dose of TC exercise programs ranged from 16 to 120 hours. | Incidence: falls; Physical performance: balance; Psychological: fear of falling; |
| Wei DY2011 | SR+meta | China | schizophrenia, IBS | 24-form simplified Yang style Tai Chi and 42-form Tai Chi, 1-7 times per week, 0.5-1 hour per session; twice daily, 60 minutes per session, for 8-48 weeks. | Physical performance; |
| Zhao Y2013 | SR+meta | China | Falls Prevention | Not available. | Incidence: falls; Physical performance: strength, ADL; |
| Li HG2011 | SR+meta | China | Essential Hypertension | 48-form or 24-form simplified Yang style Tai Chi, Chen style Tai Chi. 45 min per session, once daily, for 6 months; 60 minutes per session, 3 sessions per week, for 8 months; 60 minutes per session; twice daily, for 6 months; 40 minutes per session, once daily for 9 weeks; 90 minutes per session, 6 sessions per week, for 3 months. | Physical performance: cardiovascular function; |

**Note:** COPD, chronic obstructive pulmonary disease; TC, Tai Chi; ADL; activity of daily living; QOL, quality of life; SF, short form. SR, systematic review; RCT, randomized clinical trials; CCS, non-randomized controlled clinical studies (quasi-randomized clinical trial or observational studies such as cohort or case-control study); CS, case series; CR, case report.

**Reference of the included studies:**

| Carbonell-Baeza A2011 | Carbonell-Baeza A, Romero A, Aparicio VA, Ortega FB, Delgado-Fernández TP, et al. (2011) Preliminary findings of a 4-month Tai Chi intervention on tenderness, functional capacity, symptomatology, and quality of life in men with fibromyalgia. American Journal of Men's Health 5: 421–429. |
| --- | --- |
| Chan AWK2010 | Chan AW, Lee A, Suen LK, Tam WW. (2010) Effectiveness of a Tai chi Qigong program in promoting health-related quality of life and perceived social support in chronic obstructive pulmonary disease clients. Qual Life Res 19: 653-664. |
| Chan AWK2011 | Chan AW, Lee A, Suen LK, Tam WW. (2011) Tai chi Qigong improves lung functions and activity tolerance in COPD clients: A single blind, randomized controlled trial. Complement Ther Med 19: 3-11. |
| Chyu MC2010 | Chyu MC, James CR, Sawyer SF, Brismée JM, Xu KT, et al. (2010) Effects of tai chi exercise on posturography, gait, physical function and quality of life in postmenopausal women with osteopaenia: a randomized clinical study. Clin Rehabil 24: 1080-1090. |
| Gemmell C2006 | Gemmell C, Leathem JM. (2006) A study investigating the effects of Tai Chi Chuan: individuals with traumatic brain injury compared to controls. Brain Inj 20: 151-156. |
| Peppone LJ2010 | Peppone LJ, Mustian KM, Janelsins MC, Palesh OG, Rosier RN, et al. (2010) Effects of a structured weight-bearing exercise program on bone metabolism among breast cancer survivors: a feasibility trial. Clin Breast Cancer 10: 224-229. |
| Abbott RB2006 | Abbott RB, Hui KK, Hays RD, Li MD, Pan T. (2007) A randomized controlled trial of tai chi for tension headaches. Evid Based Complement Alternat Med 4: 107-113. |
| Ahn S2012 | Ahn S, Song R. (2012) Effects of Tai Chi Exercise on glucose control, neuropathy scores, balance, and quality of life in patients with type 2 diabetes and neuropathy. The Journal of Alternative and Complementary Medicine 18: 1172–1178. |
| Audette JF2006 | Audette JF, Jin YS, Newcomer R, Stein L, Duncan G, et al. (2006) Tai Chi versus brisk walking in elderly women. Age Ageing 35: 388-393. |
| Au-Yeung SSY2009 | Au-Yeung SS, Hui-Chan CW, Tang JC. (2009) Short-form Tai Chi improves standing balance of people with chronic stroke. Neurorehabil Neural Repair 23: 515-522. |
| Bao XM 2008 | Bao XM. (2008) A Study on the Effect of Taijiquan As An Optional Course on Students' Psychological Health. Psychological Science 31:1251-1254. |
| Barbat-Artigas S2011 | Barbat-Artigas S, Filion ME, Dupontgand S, Karelis AD, Aubertin-Leheudre M. (2011) Effects of tai chi training in dynapenic and nondynapenic postmenopausal women. The Journal of The North American Menopause Society 18: 974-979. |
| Barrow DE2007 | Barrow DE, Bedford A, Ives G, O'Toole L, Channer KS. (2007) An evaluation of the effects of Tai Chi Chuan and Chi Kung training in patients with symptomatic heart failure: a randomised controlled pilot study. Postgrad Med J 83: 717-721. |
| Bi Y 2005 | Bi Y, Chen WH. (2005) The effect of Taijiquan exercise on the hemorheology of patients with hypertension. Chin J Sports Med 24: 606-607. |
| Blake H2009 | Blake H, Batson M. (2009) Exercise intervention in brain injury: a pilot randomized study of Tai Chi Qigong. Clinical Rehabilitation 23: 589-598. |
| Brismee JM2007 | Brismee JM, Paige RL, Chyu MC, Boatright JD, Hagar JM, et al. (2007) Group and home-based tai chi in elderly subjects with knee osteoarthritis: a randomized controlled trial. Clinical Rehabilitation 21:99-111. |
| Brosseau L2004 | Brosseau L, Pelland L,Wells G, Macleay L, Lamothe C, et al. (2004) Efficacy of aerobic exercises for osteoarthritis (part II):a meta-analysis. Physical Therapy Reviews 9: 125–145 |
| Burgener SC2011 | Burgener SC, Marsh-Yant S, Nega KK. (2011) A combined, multimodal intervention for individuals with dementia. Res Gerontol Nurs 4: 64-75. |
| Cai Y 2010 | Cai Y, Zhou HF. (2010) The influence of taijiquan exercise on depressed mood. Medical Journal of Chinese People's Health 22:2051,2112. |
| Caminiti G2011 | Caminiti G, Volterrani M, Marazzi G, Cerrito A, Massaro R, et al. (2011) Tai chi enhances the effects of endurance training in the rehabilitation of elderly patients with chronic heart failure. Rehabil Res Pract 2011: ID761958. |
| Caminiti G2011 | Caminiti G, Volterrani M, Marazzi G, Cerrito A, Massaro R, et al. (2011) Tai chi enhances the effects of endurance training in the rehabilitation of elderly patients with chronic heart failure. Rehabil Res Pract 2011: 761958. |
| Campo RA2013 | Campo RA, Connor KO, Light KC, Nakamura Y, Lipschitz DL, et al. (2013) Feasibility and Acceptability of a Tai Chi Chih Randomized Controlled Trial in Senior Female Cancer Survivors. Integrative Cancer Therapies 2013: DOI: 10.1177/1534735413485418. |
| Cao J 2012 | Cao J, Cao YX, Xiao GQ. (2012) Aerobic and comprehensive practice taijiquan after liver damage and reserve function in patients with nonalcoholic fatty liver disease. Chin J Sports Med 31:1048-1102. |
| Chan K2004 | Chan K, Qin L, Lau M, Woo J, Au S, et al. (2004) A randomized, prospective study of the effects of Tai Chi Chun exercise on bone mineral density in postmenopausal women. Arch Phys Med Rehabil 85: 717-722. |
| Chang JY2011 | Chang JY, Tsai PF, Beck C, Hagen J, Huff DC, et al. (2011) The effect of tai chi on cognition in elders with cognitive impairment. Geriatr Nurs 20: 63–70. |
| Chang MY2012 | Chang MY, Chang MY, Yeh SCJ, Chu MC, Wu TM, Huang TH. (2012) Associations between Tai Chi Chung Program, Anxiety, and Cardiovascular Risk Factors. American Journal of Health Promotion 2012: DOI: 10.4278/ajhp.120720-QUAN-356. |
| Chang RY2010 | Chang RY, Koo M, Kan CB, Yu ZR, Chu IT, et al. (2010) Effects of Tai Chi rehabilitation on heart rate responses in patients with coronary artery disease. Am J Chin Med 38: 461-472. |
| Chang RY2011 | Chang RY, Koo M, Ho MY, Lin ZZ, Yu ZR, et al. (2011) Effects of Tai Chi on adiponectin and glucose homeostasis in individuals with cardiovascular risk factors. Eur J Appl Physiol 111: 57–66. |
| Chang YF2008 | Chang YF, Yang YH, Chen CC, Chiang BL. (2008) Tai Chi Chuan training improves the pulmonary function of asthmatic children. J Microbiol Immunol Infect 41: 88-95. |
| Channer KS1996 | Channer KS, Barrow D, Barrow R, Osborne M, Ives G. (1996) Changes in haemodynamic parameters following Tai Chi Chuan and aerobic exercise in patients recovering from acute myocardial infarction. Postgrad Med J 72: 349-351. |
| Chen EW2012 | Chen EW, Fu AS, Chan KM, Tsang WW. (2012) The effects of Tai Chi on the balance control of elderly persons with visual impairment_ a randomised clinical trial. Age Ageing 41: 254-259. |
| Chen FZ 2013 | Chen FZ, Lv QB. (2013) Effect of Taijiquan on blood pressure of patients with hypertension. Today Nurse 4: 18 |
| Chen GL 2007 | Chen GL. (2007) An experimental research on metacognition strategies exerting influences on Tai Ji. Dissertation for Master's Degree of Hebei Normal University. |
| Chen H 2012 | Chen H, Zhou YN. (2012) Effects of Tai Ji Quan exercise on serum TNF-α and IL-6 levels in patients with senile hypertension. Chinese Journal of Gerontology 32: 2361-2362. |
| Chen J 2009 | Chen J, Li ZW. (2009) Community respiratory rehabilitation for chronic obstructive pulmonary disease. Medical Information 22:2396-2397. |
| Chen J 2011 | Chen J. (2011) The influence of taijiquan to the elderly hypertension in community. Medical Information 24:3435-3436. |
| Chen JF 2013 | Chen JF. (2013) The effect of Taijiquan exercise on the blood lipid of patients with aged coronary heart disease. Guide of China Medicine 11: 583-584. |
| Chen JF 2013 | Chen JF. (2013) Observations of effects of integrated traditional Chinese and western medicine on stable angina pectoris in old people. Shanxi Journal of Traditional Chinese Medicine 29: 33-34. |
| Chen JY 2011 | Chen JY, Li JH, Chen WJ, Xie YL, Yang LZ, et al. (2012) Effect evaluation of Tai Chi on hypertension in Guangzhou Liurong Community. Journal of medical informatics 24:13-14. |
| Chen KM 2007 | Chen KM, Li CH, Lin JN, Chen WT, Lin HS, et al. (2007) A feasible method to enhance and maintain the health of elderly living in long-term care facilities through long-term, simplified tai chi exercises. Journal of Nursing research 15: 156-163. |
| Chen KM2007 | Chen KM, Hsu YC, Chen WT, Tseng HF. (2007) Well-being of institutionalized elders after Yang-style Tai Chi practice. Journal of Nursing 16: 845–852. |
| Chen KM2008 | Chen KM, Lin JN, Lin HS, Wu HC, Chen WT, et al. (2008) The effects of a Simplified Tai-Chi Exercise Program (STEP)on the physical health of older adults living in long-term care facilities: A single group design with multiple time points. International Journal of Nursing Studies 45: 501-507. |
| Chen L2007 | Chen L, Zhao B. (2007) Influences of different exercise prescriptions on malondialdehyde, superoxide dismutase, glutathione and glutathione-PX of the elderly people. Journal of Xinxiang Medical College 24:237-239. |
| Chen R 2011 | Chen R, Li SC. (2011) Effects of Taiji and fast-walking on the bone mineral density and metabolism in the old women. Journal of Jilin Institute of Physical Education 27: 87-88. |
| Chen YP 2004 | Chen YP, Yao HP, Yao YL, Jiang GH, Chen YW, et al. (2004) Experimental Research on Shadowboxing Prescription of Middle and Old-aged Hypertensive. Journal of Shenyang Institute of Physical Education 23:446-447. |
| Chen YS2011 | Chen YS, Zhou S, Cartwright C. (2011) Effect of 12 weeks of Tai Chi training on soleus Hoffmann reflex and control of static posture in older adults. Arch Phys Med Rehabil 92: 886-891. |
| Chen YS2012 | Chen YS, Crowley Z, Zhou S, Cartwright C. (2012) Effects of 12-week Tai Chi training on soleus H-reflex and muscle strength in older adults a pilot study. European Journal of Applied Physiology 112:2363–2368. |
| Cheung SY2007 | Cheung SY, Tsai E, Fung, NG J. (2007) Physical benefits of Tai Chi Chuan for individuals with lower-limb disabilities. Occupational Therapy International 14: 1–10. |
| Choi HJ2005 | Choi HJ, Moon JS, Song R. (2005) Effects of Sun-style Tai Chi exercise on physical fitness and fall prevention in fall-prone older adults. Journal of Advanced Nursing 2:150–157. |
| Chris J2004 | Hass CJ, Gregor RJ, Waddell DE, Oliver A, Smith DW, et al. (2004) The influence of Tai Chi training on the center of pressure trajectory during gait initiation in older adults. Arch Phys Med Rehabil 85: 1593-1598. |
| Courtney D2009 | Hall CD, Miszko T, Wolf SL. (2009) Effects of Tai Chi intervention on dual-task ability in older adults: a pilot study. Arch Phys Med Rehabil 90: 525-529. |
| Cui YQ 2010 | Cui YQ, Wu HY, Duan XH. (2010) The change of blood biochemical indices under different load taijiquan exercise analysis. Modern Preventive Medicine 37: 3881-3883. |
| Dalusung-Angosta A2011 | Dalusung-Angosta A. (2011) The impact of Tai Chi exercise on coronary heart disease: a systematic review. J Am Acad Nurse Pract 23: 376-381. |
| Day L2012 | Day L, Hill KD, Jolley D, Cicuttini F, Flicker L, et al. (2012) Impact of Tai Chi on Impairment, Functional Limitation, and Disability Among Preclinically Disabled Older People: A Randomized Controlled Trial. Arch Phys Med Rehabil 93:1400-1407 |
| Dechamps A2009 | Dechamps A, Onifade C, Decamps A, Marchasson IB. (2009) Health-related quality of life in frail institutionalized elderly effects of a cognition-action intervention and Tai Chi. Journal of Aging and Physical Activity 17:234-248. |
| Dechamps A2009 | Dechamps A, Gatta B, Bourdel-Marchasson I, Tabarin A, Roger P. (2009) Pilot study of a 10-week multidisciplinary Tai Chi intervention in sedentary obese women. Clin J Sport Med 19: 49-53. |
| Dechamps A2010 | Dechamps A, Diolez P, Thiaudière E, Tulon A, Onifade C, et al. (2010) Effects of exercise programs to prevent decline in health-related quality of life in highly deconditioned institutionalized elderly persons: a randomized controlled trial. Arch Intern Med 170: 162-169. |
| Deng YM 2001 | Deng YM. (2001) Study of effects of tai chi exercise influence on college students' mental health. Modern rehabilitation 5: 134. |
| Deng YX 2012 | Deng YX. (2012) Du moxibustion treatment of ankylosing spondylitis for adjustment of T cell subgroup. Journal of Practical Traditional Chinese Internal Medicine 26:77-78. |
| Ding FM2013 | Ding FM, Wang JH, Wang XK, Guan MD. (2013) Taijiquan combined with jogging has effected the short prognosis of patients with STEMI after PCI. Chinese Journal of Clinical Research 26: 126-127. |
| Ding M2012 | Ding M. (2012) Tai Chi for stroke rehabilitation: A focused review. Am J Phys Med Rehabil 91: 1091-1096. |
| Ding Z 2006 | Ding Z, Yang T. (2006) The influence of Taijiquan exercise for blood lipid and lipoprotein metabolism in patients with hyperlipidemia. Chinese Journal of Clinical Rehabilitation 10:172-173. |
| Du JZ 1961 | Du JZ. (1961) Positive effects of integrated traditional Chinese and western medicine on bacillary dysentery. People's Military Surgeon Z1: 21. |
| Du ST 2013 | Du ST, Ding LM, Wang CX, Yang FB, Xing B, et al. (2013) The effect of Taijiquan exercise on the exercise tolerance and lung function of patients with chronic obstructive pulmonary disease. Chin J Rehabil Med 28: 374-376. |
| Duan YM 2012 | Duan YM, Lu GT, Wang JC, Xia KX. (2012) Study on the curative effective of Taiji boxing for treating scapulohumeral periarthritis. Modern Preventive Medicine 39: 5320-5321, 5323. |
| Esch T2007 | Esch T. (2007) Mindbody techniques for physiological and psychological stress reduction stress management via Tai Chi training - a pilot study. Medical Science Monitor: International Medical Journal of Experimental and Clinical Research 13: CR488-497. |
| Evangelos A2003 | Evangelos A, Yang Y, Rosengren KS. (2003) Taiji training improves knee extensor strength and force control in older adults. Journal of Gerontology: MEDICAL SCIENCES 58: 763–766. |
| Faber MJ2006 | Faber MJ, Bosscher RJ, Chin A Paw MJ, van Wieringen PC. (2006) Effects of exercise programs on falls and mobility in frail and pre-frail older adults: A multicenter randomized controlled trial. Arch Phys Med Rehabil 87: 885-896. |
| Field T2013 | Field T, Diego M, Delgado J, Medina L. (2013) Tai chi/yoga reduces prenatal depression, anxiety and sleep disturbances. Complement Ther Clin Pract 19: 6-10. |
| Fransen M 2007 | Fransen M, Nairn L, Winstanley J, Lam P, Edmonds J. (2007) Physical activity for osteoarthritis management a randomized controlled clinical trial evaluating hydrotherapy or Tai Chi classes. Arthritis Rheum 57: 407-414. |
| Fu CY 1996 | Fu CY, Huang ZF, Wang YS, (1996) Effects of Tai Ji Quan on balanced state of autonomic nerve. China Journal of Rehabilitation Medicine 11: 88-89. |
| Fu ZL 2005 | Li F, Harmer P, Fisher KJ, McAuley E, Chaumeton N, et al. (2005) Tai Chi and fall reductions in older adults a randomized controlled trial. J Gerontol A Biol Sci Med Sci 60: 187-194. |
| Fu ZL2004 | Li F, Fisher KJ, Harmer P, Irbe D, Tearse RG, et al. (2004) Tai Chi and Self-Rated Quality of Sleep and Daytime Sleepiness in Older Adults: A Randomized Controlled Trial. J Am Geriatr Soc 52: 892-900. |
| Galantino ML 2005 | Galantino ML, Shepard K, Krafft L, Laperriere A, Ducette J, et al. (2005) The effect of group aerobic exercise and t'ai chi on functional outcomes and quality of life for persons living with acquired immunodeficiency syndrome. J Altern Complement Med 11: 1085-1092. |
| Gan JL 2007 | Gan JL, Duan HF, Gao CY. (2007) The influence of hexagram boxing training on rehabilitation of chronic schizophrenics in army men. Chin J of Behavioral Med Sci 16: 1090-1092. |
| Gan JL 2010 | Gan JL, Duan HF, Gao CY, Zhang DW, Zhang WH, et al. (2010) Effects of Taijiquan exercise on event-related potential P300 of and auditory evoked potential of patients with chronic schizophrenia. Chin J Nerv Ment Dis 36: 83, 87,99. |
| Gan JL 2010 | Gan JL, Zhang DW, Sun PZ, Duan HF, Zhu XQ, et al. (2010) A study on psychosomatic health of peacekeeping soldiers and psychological behavior intervention effect. Journal of Clinical Psychiatry 20: 361-364. |
| Gan JL 2011 | Gan JL, Duan HF, Zhu XQ, Zhao LM, Zhang DW, et al. (2011) Effects of the comprehensive psychological behavior intervention on mental health and event-related potential P300 of the servicemen under military exercise. Chin J Psychiatry 44: 17-20. |
| Gao J 2011 | Gao J, Liu W. (2011) The relation between Tai Chi Quan(Sword)exercise and mental health of the elderly. Chin J Convalescent Med 20:52-54. |
| Gao XF 2009 | Gao XF, Zhao CF. (2009) Observations of effects of acupuncture combined with Tai Ji Quan exercise on primary hypertension in 56 cases. Guide of China Medicine 7: 108-109. |
| Gao XF 2011 | Gao XF, Wang KS, Zhao CF. (2011) Chaihushugansan and Taijiquan with 56 cases of treatment of insomnia. Medical Information 3: 1188-1189. |
| Gatts SK2007 | Gatts SK, Woollacott MH. (2007) How Tai Chi improves balance biomechanics of recovery to a walking slip in impaired seniors. Gait&Posture 25:205-214. |
| Gillespie LD2003 | Gillespie LD, Gillespie WJ, Robertson MC, Lamb SE, Cumming RG, et al. (2007) Interventions for preventing falls in elderly people (Review). The Cochrane Library (4):CD000340. |
| Gloria Y2008 | Gloria YY, Joseph EM, Chung KP, Russell SP, Roger BD, et al. (2008) Enhancement of sleep stability with Tai Chi exercise in chronic heart failure: Preliminary findings using an ECG-based spectrogram method. Sleep Med 9:527-536. |
| Gong NN 2010 | Gong NN. (2010) Effect of tai chi and brisk walking exercise on hemorheology indexes of the mid-aged and old women. Dissertation for Master's Degree of Shandong Sport University. |
| Gong YH 2012 | Gong YH, Wang BJ. (2012) Study of intervention of sports for internet addiction of college students.Journal of private science and technology. Chinese Journal of Sports Medicine 10:153. |
| Greenspan AI2007 | Greenspan AI, Wolf SL, Kelley ME, O'Grady M. (2007) Tai chi and perceived health status in older adults who are transitionally frail_ a randomized controlled trial. Phys Ther 87: 525-535. |
| Gu G 2011 | Gu G. (2011) Effects of TaiChiquan exercise on exercise tolerance and life quality of patients with chronic obstructive pulmonary disease in stable phases. Dissertation for Master's Degree of Guangzhou Medical College. |
| Gu G 2012 | Gang G, Zhou YM, Wang DL, Chen L, Zhong NS, et al. (2012) Effects of shadow boxing training on exercise endurance and quality of life of patients with chronic obstructive pulmonary disease. Natl Med J China 92:952-955. |
| Guo HH 2012 | Guo HH, Zhang CH, Cai XX, Wang HH, Wang ZL, et al. (2012) Influence of doing exercise for different length of time and with different degree of intensity on serum cytokine levels in COPD patients. Journal of Hainan Medical University 18: 754-757. |
| Guo SJ 2002 | Guo SJ, Lu CS. (2002) The comparative study of the treatment of Tai Chi Quan and gate ball of the Technical Impact Index of blood lipid and blood rheology for the elderly. Chinese Joumal of Rehabilitation 6:3565. |
| Guo XN 2010 | Ni GX, Song L, Yu B, Huang CH, Lin JH. (2010) Tai Chi Improves Physical Function in Older Chinese Women With Knee Osteoarthritis. J Clin Rheumatol 16: 64-67. |
| Hackney ME2009 | Hackney ME, Earhart GM. (2009) Health-related quality of life and alternative forms of exercise in Parkinson disease. Parkinsonism Relat Disord 15: 644-648. |
| Hall A2009 | Hall A, Maher C, Latimer J, Ferreira M. (2009) The effectiveness of Tai Chi for chronic musculoskeletal pain conditions: a systematic review and meta-analysis. Arthritis Rheum 61:717-724. |
| Hall AM2011 | Hall AM, Maher CG, Lam P, Ferreira M, Latimer J. (2011) Tai chi exercise for treatment of pain and disability in people with persistent low back pain a randomizedcontrolled trial. Arthritis Care Res 63: 1576-1583. |
| Hammond A2006 | Hammond A, Freeman K. (2006) Community patient education and exercise for people with fibromyalgia: a parallel group randomized controlled trial. Clin Rehabil 20: 835-846. |
| Han A2004 | Han A, Judd M, Welch V, Wu TX, Tugwell P, Wells GA. (2010) Tai chi for treating rheumatoid arthritis. The Cochrane Library (3): CD004849. |
| Han QY 2010 | Han QY, Huang XF, Li L, Chen LQ. (2010) The effect of shadow boxing exercise on the long-term quality of life in middle-aged and elderly patients with primary hypertension. Chin J Mod Nurs 16: 1617-1619. |
| Han YB 2012 | Han YB. (2012) Effects on postmenopausal women serum adiponectin 12w tai chi training.Chinese Journal of Gerontology 32:38-40. |
| Han YT 2009 | Han YT, Li WP, Mao DW, Zhang L, Gao L. (2009) Effect of Tai Chi and brisk walking on body composition of elderly women. Proceedings of 13th National Conference on Sports Biomechanics. |
| Han YZ 2010 | Han YZ. (2010) Study on the phased evaluation of Taichi training on Lung and Heart Function and ability of balance for elderly person. Dissertation for Master's Degree of Northeast Normal University. |
| Harling A2008 | Harling A, Simpson JP. (2008) A systematic review to determine the effectiveness of Tai Chi in reducing falls and fear of falling in older adults. Physical Therapy Reviews 13:237-248. |
| Hart J2004 | Hart J, Kanner H, Gilboa-Mayo R, Haroeh-Peer O, Rozenthul-Sorokin N, et al. (2004) Tai Chi Chuan practice in community-dwelling persons after stroke. Int J Rehabil Res 27: 303-304. |
| He JH 2011 | He JH, Yao L, Chang Z, Liu GN. (2011) Rehabilitation Effect of Systematic Exercise in Adjuvant Chemotherapy for Breast Cancer Patients. Chinese Journal of Rehabilitation 26: 204-206. |
| He JH 2011 | He JH, Yao L, Chang Z, Liu GN. (2011 )Effects of Tai Chi Chuan combined with vibration training on the reflex activity of peripheral neuron. Chinese Joumal of Rehabiliation Medicine 26:968-971. |
| He YF 2009 | He YF, Jiang YC, Li F. (2009) Clinical observation of 48 cases of patients with muscle strain practicing Taijiquan. Journal of Traditional Chinese Medicine 24: 454. |
| He YP 2012 | He YP, Lv P. (2012) The effect and mechanism of Taijiquan on primary hypertension. Journal of Anhui Normal University (Natural Science) 35: 83-7. |
| Ho RTH2012 | Ho RT, Au Yeung FS, Lo PH, Law KY, Wong KO, et al. (2012) Tai Chi for residential patients with schizophrenia on movement coordination, negative symptoms, and functioning: a pilot randomized controlled trial. Evid Based Complement Alternat Med 2012: 923925. |
| Ho RTH2012 | Ho RT, Au Yeung FS, Lo PH, Law KY, Wong KO, et al. (2012) Tai-chi for residential patients with schizophrenia on movement coordination, negative symptoms, and functioning: a pilot randomized controlled trial. Evid Based Complement Alternat Med 2012: 923925. |
| Hosseini H 2011 | Hosseini H, Esfirizi MF, Marandi SM, Rezaei A. (2011) The effect of Ti Chi exercise on the sleep quality of the elderly residents in Isfahan Sadeghieh elderly home. Iran J Nurs Midwifery Res 16: 55-60. |
| Huang YY 2010 | Huang YY. (2010) Effect of Tai Ji Quan on mental health in undergraduates. Technology Trend 13: 18. |
| Hui CH2009 | Huang HC, Liu CY, Huang YT, Kernohan WG. (2010) Community-based interventions to reduce falls among older adults in Taiwan - long time follow-up randomised controlled study. J Clin Nurs 19: 959-968. |
| Hung JW2009 | Hung JW, Liou W, Wang PW, Yeh SH, Lin LW, et al. (2009) Effect of 12-week tai chi chuan exercise on peripheral nerve modulation in patients with type 2 diabetes mellitus.Journal of Rehabilitation medicine 41: 924–929. |
| Integrated Traditional Chinese and Western Medicine Group of Tuberculosis Hospital of Chongqing 1975 | Integrated Traditional Chinese and Western Medicine Group of Tuberculosis Hospital of Chongqing. (1975) A preliminary summary of LvXia Tang for tuberculosis in 21 cases. Chongqing Medical Journal 4: 50-53. |
| Irwin MR 2008 | Irwin MR, Olmstead R, Motivala SJ. (2008) Improving sleep quality in older adults with moderate sleep complaints A randomized controlled trial of Tai Chi Chih. SLEEP 31:1001-1008. |
| Irwin MR2003 | Irwin MR, Pike JL, Cole JC, Oxman MN. (2003) Effects of a behavioral intervention, Tai Chi Chih, on varicella-zoster virus specific immunity and health functioning in older adults. Psychosom Med 65: 824-830. |
| Irwin MR2007 | Irwin MR, Olmstead R, Oxman MN. (2007) Augmenting immune responses to varicella zoster virus in older adults: a randomized, controlled trial of Tai Chi. J Am Geriatr Soc 55: 511-517. |
| Irwin MR2012 | Irwin MR, Olmstead R. (2012) Mitigating cellular inflammation in older adults: a randomized controlled trial of Tai Chi Chih. Am J Geriatr Psychiatry 20: 764-772. |
| Jahnke RA2010 | Jahnke RA, Larkey LK, Rogers C, BC APRN. (2010) Dissemination and benefits of a replicable Tai Chi and Qigong program for older adults. Geriatric Nursing31: 272-280. |
| James A2012 | Mortimer JA, Ding D, Borenstein AR, DeCarli C, Guo Q, et al. (2012) Changes in brain volume and cognition in a randomized trial of exercise and social interaction in a community-based sample of non-demented Chinese elders. J Alzheimers Dis 30: 757-766. |
| Janelsins MC2011 | Janelsins MC, Davis PG, Wideman L, Katula JA, Sprod LK, et al. (2011) Effects of Tai Chi Chuan on insulin and cytokine levels in a randomized controlled pilot study on breast cancer survivors. Clin Breast Cancer 11: 161-170. |
| Ji CN 2010 | Ji CN, Liu JJ. (2010) Effects of acupuncture combined with Tai Ji Quan exercise therapy on myofascitis of the back region rehabilitation. Chinese Community Doctors 12: 127. |
| Ji CN 2010 | Ji CN, Ji CN, Liu JJ. (2010) Electroacupuncture combined impact of 24 Style of lumbar disc herniation. Guide of China Medicine18: 61-62. |
| Ji ZY 2012 | Jin ZY. (2012) Effect of Tai Chi on the hypertension and blood lipid level among middle-aged people. Mordern Preventive Medicine 39: 4468-9, 4471. |
| Jia Q 2010 | Jia Q, Zhu LL. (2010) Effect of Shadow boxing combined with psychological intervention on risk factors and adiponectin of metabolic syndrome. Chin J Cardiovasc Reh abil Med 19: 337-339, 345. |
| Jiang JX 1984 | Jiang JX. (1984) Taijiquan on serum high density lipoprotein cholesterol (HDL - C) and other blood lipid effect observation. Chinese Journal of Sports Medicine 3:99-101,128. |
| Jiang MY 2013 | Jiang MY, Wang M, Song CA. (2013) Influence of shadowboxing on improving cancer-related fatigue and sleeping quality of patients with advanced lung cancer. Chinese Nursing Research 27: 420-421. |
| Jiang XL 2010 | Jiang XL, Wang KS. (2010) Tai Ji Quan exercise for the treatment of scapulohumeral periarthritis. China Foreign Medical Treatment 5: 75. |
| Jiang Y 2013 | Jiang Y, Wan K, Xiao YG. (2013) Traditional sports research effect on medical students' mental health intervention. Hubei Journal of TCM 35: 27. |
| Jin H 2000 | Jin H. (2000) The influence of Tai Chi exercise on human peripheral blood T cell subsets. Journal of Beijing University of Chinese Medicine 15:16-17. |
| Jin H 2012 | Jin H. (2012) Experimental control of different intervention methods in Higher Vocational College students with psychological sub-health. Chinese Community Doctors 14: 150-151. |
| Jin M 2012 | Jin M, Ma LN, Qi XY. (2012) The case report of "Ten" the wheelchair taijiquan of mental health in patients with spinal cord injury. Chinese journal Rehabil Theory Practice 18: 386-388. |
| Jin XQ2010 | Jin XQ. (2010) The comparative study on the fitness effect of the elderly by taking health Qigong, Tai Chi and fitness Yangko. Dissertation for Master Degree of Hebei Normal University. |
| Jing JH 2012 | Jing JH, Liu ZK. (2012) Different sports exercise on fatigue subhealth Students basic physical fitness Effect of improvement.Contemporary Sports Technology 2: 13-14. |
| Jing XL2009 | Li JX, Xu DQ, Hong Y. (2009) Changes in muscle strength, endurance, and reaction of the lower extremities with Tai Chi intervention. J Biomech 42: 967-971. |
| Jones AY2005 | Jones AY, Dean E, Scudds RJ. (2005) Effectiveness of a community-based Tai Chi program and implications for public health initiatives. Arch Phys Med Rehabil 86: 619-625. |
| Jung S2012 | Jung S, Lee EN, Lee SR, Kim MS, Lee MS. (2012) Tai chi for lower urinary tract symptoms and quality of life in elderly patients with benign prostate hypertrophy. Evid Based Complement Alternat Med 2012: 624692. |
| Kang JW2011 | Kang JW, Lee MS, Posadzki P, Ernst E. (2011) Tai chi for the treatment of osteoarthritis: a systematic review and meta-analysis. BMJ Open 1: e000035. |
| Karen M2004 | Mustian KM, Katula JA, Gill DL, Roscoe JA, Lang D, et al. (2004) Tai Chi Chuan, health-related quality of life and self-esteem_ a randomized trial with breast cancer survivors. Support Care Cancer 12: 871-876. |
| Ke JB 2007 | Ke JB, Ma WL, Zhong M, Jiang L, Lin JD, et al. (2007) Global gene expression in human skeletal muscle of the elderly person influenced by Tai Chi Chuan training. Chinese Journal of Rehabilitation Medicine. 22: 306-309, 322. |
| Ke JB 2009 | Ke JB, Nan N, Yang WB, Sun Y, Xiong YY, et al. (2009) Global gene expression in human skeletal muscle of the COPD patient through aerobic exercise. Chinese Journal of Rehabilitation Medicine 24: 690-694, 670. |
| Kim D2012 | Jones KD, Sherman CA, Mist SD, Carson JW, Bennett RM, et al. (2012) A randomized controlled trial of 8-form Tai chi improves symptoms and functional mobility in fibromyalgia patients. Clin Rheumatol 31: 1205-1214. |
| Kluding P 2006 | Kluding P, McGinnis PQ. (2006) Multidimensional exercise for people with Parkinson's disease a case report. Physiotherapy Theory and Practice 22:153-162. |
| Kuang GJ 2005 | Kuang GJ, Lin DP. (2005) Effects of 42-form Tai Ji Quan exercise on chronic heart failure in 60 cases. Traditional Chinese Medicinal Research 18: 49-50. |
| Kui RQ 1990 | Kui RQ, Lin YH, Sun YX, Zhou N. (1990) The effect of Qigong and Taijiquan on pulmonary function in the elderly. Chinese Journal of Rehabilitation Medicine 5:115-117,130. |
| Kutner NG1997 | Kutner NG, Barnhart H, Wolf SL, McNeely E, Xu T. (1997) Self-Report Benefits of Tai Chi Practice by Older Adults. J Gerontol B Psychol Sci Soc Sci 52: 242-246. |
| Lam LCW2011 | Lam LCW, Chau RCM, Wong BML, Fung AWT, Lui VWC, et al. (2011) Interim followup of a randomized controlled trial comparing Chinese style mind body (Tai Chi) and stretching exercises on cognitive function in subjects at risk of progressive cognitive decline. international Journal of Geriatric Psychiatry 26:733-740. |
| Lam P2008 | Lam P, Dennis SM, Diamond TH. (2008) Improving glycaemic and BP control in type 2 diabetes The effectiveness of tai chi. Australian Family Physician 37:884-887. |
| Lan C1999 | Lan C, Chen SY, Lai JS, Wong MK. (1999) The effect of Tai Chi on cardiorespiratory function in patients with coronary artery bypass. Medicine & AMP Science in Sports & Exercise 31: 634-638. |
| Lan C2000 | Lan C, Lai JS, Chen SY, Wong MK .(2000) Tai Chi Chuan to improve muscular strength and endurance in elderly individuals:a pilot study. Archives of Physical Medicine and Rehabilitation 2000: DOI: 10.1016/S0003-9993(00)90042-X. |
| Lan C2008 | Lan C, Ching Lan, Ssu-Yuan Chen and Jin-Shin Lai. (2008) Changes of aerobic capacity, fat ratio and flexibility in older TCC practitioners a five-year follow-up. The American Journal of Chinese Medicine 36:1041-1050. |
| Lan YS2009 | Lan YS. (2009) Effects of 24 form TCC practice on congnitive function. Dissertation for Master Degree of Shandong Normal University. |
| Lavretsky H2011 | Lavretsky H, Alstein LL, Olmstead RE, Ercoli LM, Riparetti-Brown M, et al. (2011) Complementary use of tai chi chih augments escitalopram treatment of geriatric depression: a randomized controlled trial. Am J Geriatr Psychiatry 19: 839-850. |
| Lee EN2004 | Lee EN. (2004) The effects of tai chi exercise program on blood pressure, total cholesterol and cortisol level in patients withessential hypertension. Taehan Kanho Hakhoe Chi 34: 829-837. |
| Lee EN2007 | Lee EN, Kim YH, Chung WT, Lee MS. (2007) Tai chi for disease activity and flexibility in patients with ankylosing spondylitis a controlled clinical trial. eCAM 5: 457–462. |
| Lee EO2010 | Lee EO, Chae YR, Song R, Eom A, Lam P, et al. (2010) Feasibility and effects of a tai chi self-help education program for Korean gastric cancer survivors. Oncology Nursing 37: 1-6. |
| Lee HY2006 | Lee HY, Lee EO. (2006) Comparison of effects among Tai-Chi exercise, aquatic exercise, and a self-help program for patients with knee osteoarthritis. The 5th International Nursing Conference 10: 72. |
| Lee HY2008 | Lee HY, Lee KJ. (2008) Effects of Tai Chi exercise in elderly with knee osteoarthritis. J Korean Acad Nurs 38: 11-18. |
| Lee HY2012 | Lee HY, Hale CA, Hemingway B, Woolridge MW. (2009) Tai Chi exercise and auricular acupressure for people with rheumatoid arthritis an evaluation study. Journal of Nursing 21: 2812–2822. |
| Lee KY2006 | Lee KY, Jeong OY. (2006) The Effect of Tai Chi Movement in Patients with Rheumatoid Arthritis. Taehan Kanho Hakhoe Chi 36: 278-285. |
| Lee LYK2007 | Lee LYK, Lee DTF, Fprkam JW, Frcpe F, Frcp et al. (2007) Effect of Tai Chi on state self-esteem and health-related quality of life in older Chinese residential care home residents. Journal of clinical nursing 16:1580-1580. |
| Lee LYK2009 | Lee LYK, Diana Lee TF, Woo J, Frakm, Fracp, et al. (2009) Tai Chi and health-related quality of life in nursing home residents. Journal of Nursing Scholarship 41: 35–43. |
| Lee LYK2010 | Lee LYK, Lee DTF, Woo J. (2010) The psychosocial effect of Tai Chi on nursing home residents. Journal of Nursing 19: 927–938. |
| Lee LYK2012 | Lee LYK, Chong YL, Li NY, Li MC, Lin LN, et al. (2012) Feasibility and effectiveness of a Chen-style Tai Chi programme for stress reduction in junior secondary school students. Stress and Health 29: 117–124. |
| Lee MS2007 | Lee MS, Pittler MH, Ernst E. (2007) Is tai chi an effective adjunct in cancer care? A systematic review of controlled clinical trials. Support Care Cancer 15: 597-601. |
| Lee MS2007 | Lee MS, Pittler MH, Ernst E. (2007) Tai chi for rheumatoid arthritis: systematic review. Rheumatology 46: 1648-1651. |
| Lee MS2007 | Lee MS, Pittler MH, Taylor-Piliae RE, Ernst E. (2007) Tai chi for cardiovascular disease and its risk factors: a systematic review. J Hypertens 25: 1974-1975. |
| Lee MS2008 | Lee MS, Lam P, Ernst E. (2008) Effectiveness of tai chi for Parkinson’s disease: a critical review. Parkinsonism Relat Disord 14: 589-594. |
| Lee MS2008a | Lee MS, Pittler MH, Ernst E. (2008) Tai chi for osteoarthritis: a systematic review. Clinical Rheumatology 27: 211–218. |
| Lee MS2008b | Lee MS, Pittler MH, Shin BC, Ernst E. (2008) Tai chi for osteoporosis: a systematic review. Osteoporos Int 19: 139–146. |
| Lee MS2009 | Lee MS, Lee EN, Ernst E. (2009) Is tai chi beneficial for improving aerobic capacity? A systematic review. Br J Sports Med 43: 569-573. |
| Lee MS2010 | Lee MS, Choi TY, Ernst E. (2010) Tai chi for breast cancer patients: a systematic review. Breast Cancer Res Treat 120: 309-316. |
| Lee MS2010 | Lee MS, Lee EN, Kim JI, Ernst E. (2011) Tai chi for lowering resting blood pressure in the elderly: a systematic review. J Eval Clin Pract 16: 818-24. |
| Lee MS2011 | Lee MS, Choi TY, Lim HJ, Ernst E. (2011) Tai Chi for Management of Type 2 Diabetes Mellitus: A Systematic Review. Chin J Integr Med 17:789-793. |
| Lei P2013 | Pan L, Yan J, Guo Y, Yan J. (2013) Effects of Tai Chi training on exercise capacity and quality of life in patients with chronic heart failure: a meta-analysis. Eur J Heart Fail 15: 316-323. |
| Lelard T2010 | Lelard T, Doutrellot PL, David P, Ahmaidi S. (2010) Effects of a 12-week Tai Chi Chuan program versus a balance training program on postural control and walking ability in older people. Arch Phys Med Rehabil 91: 9-14. |
| Leung DP2011 | Leung DP, Chan CK, Tsang HW, Tsang WW, Jones AY. (2011) Tai chi as an intervention to improve balance and reduce falls in older adults: A systematic and meta-analytical review. Altern Ther Health Med 17: 40-48. |
| Li C2009 | Li C, Yu Y, Xu FL. Observation on the effect of simple Taijiquan on seniors with insomnia in community. Shanghai Medical & Pharmaceutical Journal 31: 66-67. |
| Li CL 2008 | Li CL, Feng HY, Xiao SC, Huang HY. (2008) The influence of Tai Ji Quan on the therapeutic effect for irritable bowel syndrome. Journal of Practical Medical Techniques 15: 1384-1386. |
| Li CW 2007 | Li CW, Zeng Y, Peng FL. (2007) Effect of Taijiquan on endothelial diastolic function in the elderly. Journal of Clinical Rehabilitative Tissue Engineering Research 27:5414-5416. |
| Li DX2013 | Li DX, Zhuang XY, Zhang YP, Guo H, Wang Z, et al. (2013) Effects of Tai Chi on the protracted abstinence syndrome: a time trial analysis. Am J Chin Med 41: 43-57. |
| Li F2007 | Li F, Harmer, Fisher KJ, Xu J, Fitzgerald K, Vongjaturapat N. (2007) Tai Chi based exercise for older adults with Parkinson's disease a pilot program evaluation. Journal of Aging and Physical Activity 15: 139-151. |
| Li FZ2001 | Li F, Harmer P, McAuley E, Duncan TE, Duncan SC, et al. (2001) An evaluation of the effects of Tai Chi exercise on physical function among older persons: a randomized contolled trial. Ann Behav Med 23: 139-146. |
| Li FZ2001 | Li F, Harmer P, McAuley E, Fisher KJ, Duncan TE, et al. (2001) Tai Chi, self-efficacy, and physical function in the elderly. Prev Sci 2: 229-239. |
| Li FZ2002 | Li F, Fisher KJ, Harmer P, McAuley E. (2002) Delineating the impact of Tai Chi training on physical function among the elderly. Am J Prev Med 23: 92-97. |
| Li FZ2004 | Li F, Harmer P, Fisher KJ, McAuley E. (2004) Tai Chi: improving functional balance and predicting subsequent falls in older persons. Med Sci Sports Exerc 36: 2046-2052. |
| Li FZ2005 | Fuzhong Li FZ, Fisher KJ, Harmer P, McAuley E. (2005) Falls Self-Efficacy as a Mediator of Fear of Falling in an Exercise Intervention for Older Adults. Journal of Gerontology:PSYCHOLOGICAL SCIENCES 60: 34–40. |
| Li FZ2008 | Li FZ, Peter H, Russell G, Karin MA, et al. (2008) Translation of an effective tai chi intervention into a community-based falls-prevention program. American Journal of Public Health 98: 1195-1198. |
| Li FZ2012 | Li F, Harmer P, Fitzgerald K, Eckstrom E, Stock R, et al. (2012) Tai chi and postural stability in patients with Parkinson's disease. N Engl J Med 366: 511-519. |
| Li G2012 | Li G, Wang JC. (2012) The significance of *Chan Si strength* of Chen-style Tai Chi for the treatment of lumbar disc herniation. Journal of Harbin Medical University 46: 403-404. |
| Li GP2011 | Li GP, Zuo XH, Xie W, Zhang YJ, Duan GX. (2011) The effect of Taijiquan exercise on gerotranscendence of the elderly in Community. Chin J Gerontol 31:1129-1131. |
| Li HG2011 | Li HG, Xu ZW. (2013) Tai Chi for the treatment of essential hypertension: a systematic review. Science & technology of stationery & sporting goods (7): 35-37. |
| Li HY2012 | Li HY. (2012) The effect of Tai Chi training and the ProKin balance therapy system training on the equilibrium function in hemiplegic patient after stroke. Dissertation for Master Degree of Soochow University |
| Li J 2007 | Li JG, Liu GB. (2007) Application of elim inating inflammatory granules and easy Tai Ji Quan of 24 styles in the stable form of carpal scaphoid bone fractures. Guiding Journal of TCM 13: 39-40, 51. |
| Li JQ2007 | Li JQ, Wang MC, Lin F. (2007) The effects of chronic disease self-management program for Chinese patients in Shenzhen. Chinese Journal of Rehabilitation 22: 289-291. |
| Li JX2008 | Li JX, Xu DQ, Hong Y. (2008) Effects of 16-week Tai Chi intervention on postural stability and proprioception of knee and ankle in older people. Age Ageing 37: 575-578. |
| Li JZ 2006 | Li JZ, Zhai DP, Qiu C, Zhao X, Wang XW, et al. (2006) The antioxidant effect of taijiquan exercise on coronary heart disease (CHD) patients Chinese .Journal of Clinical Rehabilitation 10:155-156. |
| Li L2010 | Li L, Manor B. (2010) Long term Tai Chi exercise improves physical performance among people with peripheral neuropathy. The American Journal of Chinese Medicine 38: 449–459. |
| Li N 2006 | Li N, Dong YP, Zeng XY. (2007) Study on the electroencephalogram characteristic of 24 style Taijiquan exercise. Journal of Wuhan Institute of Physical Education 5: 50-52, 73. |
| Li Q 2012 | Li Q, Fang WH, Liu C. (2012) The effect of taijiquan combined with respiratory exercise training on rehabilitation of patients with stable chronic obstructive pulmonary disease. Chinese Journal of Rehabilitation Medicine 27: 825-828. |
| Li RZ2013 | Li RZ, Liu YH, Wang LX, Zhao XH, Pi XH. (2013) Effect of Naoling decoction combined with Taichi on the rehabilitation of patients with Alzheime's disease.Modern Journal of Integrated Traditional Chinese and Western Medicine 22: 693, 760. |
| Li X 2011 | Li X, Yang SL, Li TJ, Cai SF. (2011) Taijiquan exercise imagination in the application of rehabilitation therapy in patients with cerebral apoplex. Fujian Journal of TCM 42:5-6. |
| Li YH 2003 | Li YH, Yu ZX. (2003) Influence of Taijiquan on blood rheology in the female seniors. Chinese Journal of Clinical Rehabilitation 7: 651. |
| Li YH2007 | Li YH, Devault CN, Oteghen SV. (2007) Effects of extended Tai Chi intervention on balance and selected motor functions of the elderly. The American Journal of Chinese Medicine 35: 383–391. |
| Li YQ 2011 | Li YQ, Zhang Y. (2011) The impact on the strain of back pain practice tai chi - the "Yellow Emperor", "disease" theory application in orthopedics. Gansu Journal of TCM 24:63-64. |
| Liang YS 2010 | Liang YS, Xie S, Zhang Y, Wang LP, Zhu CL. (2010) Treatment of 40 cases of irritable bowel syndrome by acupoint thread burial therapy and taijiquan. SH. J. TCM 44: 38-39, 43. |
| Liang YS 2010a | Liang YS, Zhang Y, Xie S, Wang LP, Zhu CL, et al. (2010) Observations on the efficacy of acupoint catgut embedding plus Taijiquan in treating irritable bowel syndrome of constipation type. Shanghai J Acu-mox 29: 634-635. |
| Liao GX 2012 | Liao GX. (2012) Effect of Taijiquan on depression and quality of life of Empty Nester. Journal of Community Medicine 10: 5-6. |
| Lin HZ2012 | Lin HZ. (2012) Clinical observation on effect of Irbesartan combined with Taijiquan movement in the treatment of early diabetic nephropathy. Chinese journal of ethnomedicine 8: 108-109. |
| Lin MR2006 | Lin MR, Hwang HF, Wang YW, Chang SH, Wolf SL. (2006) Community-based tai chi and its effect on injurious falls balance gait and fear of falling in older people. Physical Therapy 86:1189-1200. |
| Lin YB 2009 | Lin YB, Zhang SJ, Ye ZH, Pan HT. (2009) The influence of exercise prescription intervention for older women's physical and mental health. Chinese Journal of Gerontology 29:350-352. |
| Ling JH 2012 | Ling JH. (2012) Clinical observation on effect of Telmisartan combined with Taijiquan movement in the treatment of early diabetic Nephropathy. Chinese Manipulation & Rehabbilitaion Medicine 3: 31-32. |
| Ling KW2003 | Ling KW, Wong FSY, Chan Wk, Chan SY, Chan EPY, et al. (2003) Effect of a home exercise program based on tai chi in patients with end-stage renal disease. Peritoneal Dialysis International 23: 99-103. |
| Liu B2011 | Liu B, Liu ZH, Zhu HE, Mo JC, Cheng DH. (2011) Effects of tai chi on lower-limb myodynamia in the elderly people: a meta-analysis. J Tradit Chin Med 31: 141-146. |
| Liu C 2003 | Liu C, Hua CL, Wang W. (2003) Observation on the efficacy of Tuina plus taijiquan in treating chronic lumbar strain. Chinese Manipulation & Qi Gong therapy 19: 14. |
| Liu CZ 2010 | Liu CZ, Lei B. (2010) Effect of Tuina on oxygen free radicals metabol ism in patients with chronic fatigue syndrome.Chinese Acupuncture & Moxibustion 30: 946-948. |
| Liu CZ 2010a | Liu CZ, Lei B. (2010) A randomized controlled trial of manipulation for chronic fatigue syndrome. Liaoning Journal of Traditional Chinese Medicine 37: 272-273. |
| Liu D2008 | Liu D, Gao L. (2008) Experimental study of effect on bone mass in female seniors after 16-week Taijiquan or brisk walking exercise. Conference Proceedings of People Physical Exercise Conference 2008:128. |
| Liu D2010 | Liu D. (2010) Effect of tai chi and brisk walking exercise on bone mass in the middle-aged elderly women. Dissertation for Master's Degree of Shandong Sport University. |
| Liu H2010 | Liu H, Frank A. (2010) Tai chi as a balance improvement exercise for older adults: a systematic review. J Geriatr Phys Ther 33: 103-109. |
| Liu HL 2005 | Liu HL. (2005) Research on how health care massage of traditional Chinese medicine improves the living qualities of the old. Dissertation for Doctor's Degree of Guangzhou University of Traditional Chinese Medicine. |
| Liu J 2003 | Liu J, Chen PJ, Qiu PX, Chen XF. (2003) A tracking study of Effects of long-term Tai Ji Quan exercise on cardiopulmonary function in middle-aged and old women. Chinese Journal of Sports Medicine 22: 290-293. |
| Liu J 2007 | Liu J, Chen PJ, Qiu PX. (2007) Effects of long-term Tai Ji Quan exercise on NKT cells in middle-aged and old women. Chinese Journal of Sports Medicine 26: 738-739. |
| Liu J 2009 | Liu J, Chen PJ, Wang R. (2009) Effects of Tai Ji Quan exercise on white cell cytokine IFN-γ and IL-4 in peripheral blood in middle-aged and old women. Chinese Journal of Sports Medicine 28: 557-558. |
| Liu J2012 | Liu J, Wang XQ, Zheng JJ, Pan YJ, Hua YH, et al. (2012) Effects of Tai Chi versus Proprioception Exercise Program on Neuromuscular Function of the Ankle in Elderly People: A Randomized Controlled Trial. Evid Based Complement Alternat Med 2012: ID265486. |
| Liu JS 1991 | Liu JS, Ren HY, Liu YF, Liu ZJ. (1991) Observations of effects of Tai Ji Quan exercise on cardiac function. Journal of Convalescence & Rehabilitation 24: 29-31. |
| Liu JW 2006 | Liu JW, Zhang XX. (2006) The influence of Tai chi on high altitude military psychological status of the Tibetan plateau. Chin J of Behavioral Med Sci 15:373. |
| Liu MR2008 | Liu MR, So HY. (2008) Effects of Tai Chi Exercise Program on Physical Fitness, Fall related Perception and Health Status in Institutionalized Elders. J Korean Acad Nurs 38: 620-628. |
| Liu Q 2011 | Liu Q, Yang GY, Chen WQ, Xiang ZY, Zhuang RR. (2011) Effects of Tai Chi Chuan combined with vibration training on the reflex activity of peripheral neuron. Joumal of Medical Biomechanic 26:329-334. |
| Liu SK 2010 | Sha KH, Liu TG, Wang H, Yu HB. (2010) Effects of Tai Ji Quan exercise on body parameters in elderly women. Chinese Journal of Gerontology. 30: 848-9. |
| Liu TJ2009 | Liu TJ, Qin P, Chen XZ. (2009) Effect of Taijiquan on the balance function of stroke patients. Chin J Phys Med Rehabil 31: 781-782. |
| Liu X 2008 | Liu X, Miller YD, Burton NW, Brown WJ. (2008) A preliminary study of the effects of Tai Chi and Qigong medical exercise on indicators of metabolic syndrome, glycaemic control. Br J Sports Med 44: 704–709. |
| Liu X2012 | Liu X, Miller YD, Burton NW, Chang JH, Brown WJ. (2013) The effect of Tai Chi on health-related quality of life in people with elevated blood glucose or diabetes: a randomized controlled trial. Qual Life Res 22: 1783-1786. |
| Liu XD 2010 | Liu XD, Jin HZ. (2010) Observa tion on Effect of TaiChiChuan on Cardiopulmonary Function of Older People. China Prac Med 5:34-35. |
| Liu XK 2011 | Liu XK. (2011) Effective comparison study of psychological intervention of Taijiquan and group counseling. Dissertation for Master's Degree of Shandong Medical University. |
| Liu Y 2007 | Liu Y, Wang CG. (2007) Chen tai chi practice combining with the sports rehabilitation corrective scoliosis 40 cases, curative effect observation. Guiding Journal of TCM 13:42-43. |
| Liu YY 2003 | Liu YY. (2003) Different projects of small and medium strength of special elective course influence on female students' state of mind and mental health. Dissertation for Master's Degree of Yangzhou University. |
| Logghe IH2010 | Logghe IH, Verhagen AP, Rademaker AC, Bierma-Zeinstra SM, van Rossum E, et al. (2010) The effects of Tai Chi on fall prevention, fear of falling and balance in older people: a meta-analysis. Prev Med 51:222-227. |
| Logghe IHJ2009 | Logghe IH, Zeeuwe PE, Verhagen AP, Wijnen-Sponselee RM, Willemsen SP, et al. (2009) Lack of effect of Tai Chi Chuan in preventing falls in elderly people living at home a randomized clinical trial. J Am Geriatr Soc 57: 70-75. |
| Logghe IHJ2011 | Logghe IHJ, Verhagen AP, Rademaker ACHJ, Zeeuwe PEM, Bierma-Zeinstra SMA, et al. (2011) Explaining the ineffectiveness of a Tai Chi fall prevention training for community-living older people a process evaluation alongside a randomized clinical trial (RCT). Archives of Gerontology and Geriatrics 52: 357-362. |
| Lu H 2007 | Luo H. (2007) Effect of Taijiquan combined with Chinese drugs on rheumatoid arthritis. Chin J Rehabil Theory Pract 13: 397-398. |
| Lu X2013 | Lu X, Hui-Chan CW, Tsang WW. (2013) Effects of Tai Chi training on arterial compliance and muscle strength in female seniors: a randomized clinical trial. Eur J Prev Cardiol 20: 238-245. |
| Luo BB 2012 | Luo BB, Wang R, Chen PJ. (2012) The effects of Tai Ji Quan exercise on the Th1/Th2 balance in middle- and older-aged women. Chin J Sports Med 31: 396-401. |
| Luo D2012 | Luo D, Li L, Li YH, Zhou T, Tao ZH, et al (2012) The impact of different sports on physical and mental health of elderly. Today Nurse 8: 1-4. |
| Luo H 2006 | Luo H. (2006) Effect of Taijiquan combined with Chinese drugs on primary hypertension. China Medical Herald 3: 43-44. |
| Lv JB 1987 | Lv JB, Wang YF, Wu M, Zha ZB. (1987) Preliminary observations of therapeutic effect of Qi Gong and Tai Ji Quan exercise for senile hypertension. Journal of West China University of Medical Sciences 1: 37-39. |
| Ma XP 2003 | Ma XP, Song WM. (2003) Effects of Tai Ji Quan on physical functions in gerontal patients. Chinese Journal of Clinical Rehabilitation 7: 2636. |
| Ma XW 2006 | Ma XW, Wang WZ. (2006) Clincal research on 32 cases to prevent losing sclerotin of elderly men . Shaanxi J Tradit Chin Med 27: 1214-1215. |
| Ma ZJ 2012 | Ma ZJ. (2012) Eercise prescription of dyslipidemia prevention in the elderly. Sports World 9: 53-54. |
| Maciaszek J2007 | Maciaszek J, Osiński W, Szeklicki R, Stemplewski R. (2007) Effect of Tai Chi on body balance: randomized controlled trial in men with osteopenia or osteoporosis. Am J Chin Med 35: 1-9. |
| Maciaszek J2010 | Maciaszek J, Osiński W. (2010) The effects of Tai Chi on body balance in elderly people--a review of studies from the early 21st century. Am J Chin Med 38: 219-229. |
| Maciaszek J2012 | Maciaszek J, Osinski W. (2012) Effect of Tai Chi on body balance: randomized controlled trial in elderly men with dizziness. Am J Chin Med 40: 245-253. |
| Madeleine E2008 | Hackney ME, Earhart GM. (2008) Tai Chi improves balance and mobility in people with Parkinson disease. Gait Posture 28: 456-460. |
| Mansky P2013 | Mansky P, Sannes T, Wallerstedt D, Ge A, Ryan M, et al. (2006) Tai chi chuan: mind-body practice or exercise intervention? Studying the benefit for cancer survivors. Integr Cancer Ther 5: 192-201. |
| Mao HN2006 | Mao HN, Sha P. (2006) Effect of Tai Chi exercise on blood pressure, plasma nitrogen monoxidum and endothelin in hypertensive patients. Chinese Journal of Clinical Rehabilitation 10: 65-67. |
| Mao HN2009 | Mao HN. (2009) Effects of taijiquan exercises combined with orally calcium supplement on postmenopausal women's bone mineral density. Chinese Journal of Rehabilitation Medicine 24: 814-816. |
| Mao Y 2007 | Mao Y, Ma LJ, Lv YA, Han XY. (2007) The influences of Taijiquan and comprehensive exercises on blood lipid metabolism of senile women. Chinese Journal of Woman and Child Health Research 18: 374-376. |
| McCain NL2008 | McCain NL, Gray DP, Elswick RK, Robins JW, Tuck I, et al. (2008) A randomized clinical trial of alternative stress management interventions in persons with HIV infection. J Consult Clin Psychol 76: 431-441. |
| McGibbon CA2004 | McGibbon CA, Krebs DE, Wolf SL, Wayne PM, Scarborough DM, et al. (2004) Tai Chi and vestibular rehabilitation effects on gaze and whole-body stability. J Vestib Res 14: 467-478. |
| McGibbon CA2005 | McGibbon CA, Krebs DE, Parker SW, Scarborough DM, Wayne PM, et al. (2005) Tai Chi and vestibular rehabilitation improve vestibulopathic gait via different neuromuscular mechanisms preliminary report. BMC Neurol 5: 3. |
| Motivala SJ 2006 | Motivala SJ, Sollers J, Thayer J, Irwin MR. (2006) Tai Chi Chih acutely decreases sympathetic nervous system activity in older adults. J Gerontol A Biol Sci Med Sci 61: 1177-1180. |
| Mustian KM2006 | Mustian KM, Katula JA, Zhao H. (2006) A pilot study to assess the influence of tai chi chuan on functional capacity among breast cancer survivors. J Support Oncol 4: 139-145. |
| Nedeljkovic M2012 | Nedeljkovic M, Ausfeld-Hafter B, Streitberger K, Seiler R, Wirtz PH. (2012) Taiji practice attenuates psychobiological stress reactivity--a randomized controlled trial in healthy subjects. Psychoneuroendocrinology 37: 1171-1180. |
| Ng SM2012 | Ng SM, Wang CW, Ho RT, Ziea TC, He J, et al. (2012) Tai chi exercise for patients with heart disease: a systematic review of controlled clinical trials. Altern Ther Health Med 18: 16-22. |
| Nguyen MH2012 | Nguyen MH, Kruse A. (2012) Randomized controlled trial of Tai chi for balance, sleep quality and cognitive performance in elderly Vietnamese. Clin Interv Aging 7: 185-190. |
| Ni HY 2000 | Ni HY, Lei XS. (2000) Hemorheology effects of 42-form Tai Ji Quan exercise in middle-aged and old intellectuals. Chinese Journal of Sports Medicine 19: 194-196. |
| Ni HY 2001 | Ni HY, Lei XS, Ye HJ, Zheng YZ. (2001) Effects of a 42-form Tai Ji Quan practice on cardiovascular fitness and blood condition in Middle-aged and old intellectuals. Chinese Journal of Sports Medicine 20: 102-4. |
| Ni J 2010 | Ni J, Shen GY, Su JY, Ju SQ, Zhu ZJ. (2010) Effect of long-term Taijiquan exercise on the ischemia modified albumin of patients with coronary heart disease. Chongqing Medicine 39: 320-322. |
| Nie CR 1961 | Nie CR, Yu SY. (1962) Effects of integrated traditional Chinese and western medicine on ulcer in 108 cases. People's Military Surgeon 2: 3-5. |
| Nnodim JO2006 | Nnodim JO, Strasburg D, Nabozny M, Nyquist L, Galecki A, et al. (2006) Dynamic balance and stepping versus tai chi training to improve balance and stepping in at-risk older adults. Journal of the American Geriatrics Society 54:1825-1831. |
| Nomura T2011 | Nomura T, Nagano K, Takato J, Ueki S, Matsuzaki Y, et al. (2011) The development of a Tai Chi exercise regimen for the prevention of conditions requiring long-term care in Japan.Archives of Gerontology and Geriatrics 52: 198–203. |
| Nowalk MP2001 | Nowalk MP, Prendergast JM, Bayles CM, D'Amico FJ, Colvin GC. (2001) A randomized trial of exercise programs among older individuals living in two long-term care facilities: the FallsFREE program. J Am Geriatr Soc 49: 859-865. |
| Palasuwan A2011 | Palasuwan A, Sukso D, Margaritis I, Soogarun S, Rousseau AS. (2011) Effects of tai chi training on antioxidant capacity in pre- and postmenopausal women. Journal of Aging Research 20: 1-8. |
| Palumbo MV2012 | Palumbo MV, Wu G, Shaner-McRae H, Rambur B, McIntosh B. (2012) Tai Chi for older nurses a workplace wellness pilot study. Appl Nurs Res 25: 54-59. |
| Pan Y 2011 | Pan Y. (2011) Effects of Tai Ji Quan on anxious emotion in gerontal patients. Chinese Journal of Clinical Healthcare 14: 74-75. |
| Pan ZJ1999 | Pan ZJ, Cao Y. (1999) Influence of Taijiquan exercise on lipid metabolism of college students. Chinese Journal of Sports Medicine 18: 380-382. |
| Park IS2010 | Park IS, Song R, Oh KO, So HY, Kim DS, et al. (2010) Managing cardiovascular risks with Tai Chi in people with coronary artery disease. Journal of Advanced Nursing 66: 282–292. |
| Pluchino A2012 | Pluchino A, Lee SY, Asfour S, Roos BA, Signorile JF. (2012) Pilot Study Comparing Changes in Postural Control After Training Using a Video Game Balance Board Program and 2 Standard Activity-Based Balance Intervention Programs. Arch Phys Med Rehabil 93: 1138-1146. |
| Qian GQ2012 | Qian G, Xue K, Tang L, Wang F, Song X, et al. (2012) Mitigation of oxidative damage by green tea polyphenols and Tai Chi exercise in postmenopausal women with osteopenia. PLoS One 7: e48090. |
| Qian JA 1986 | Qian JA, Gong LS, Yang Q, Jiang J, Ye JP, et al. (1986) Effects of Tai Ji Quan exercise in patients with cardiopathy. Acta Universitatis Medicinalis Secondae Shanghai 6: 366-367, 369. |
| Qian YW 1962 | Qian YN, Chen DF, Wu HM. (1962) Amateurish integrated traditional Chinese and Western medicines for the treatment of hypertension in 65 cases. People's Military Surgeon 2: 11-12. |
| Qin L 2006 | Qin L, Chen QM, Ou SQ, Cai YY, Kong YY, et al. (2006) Taijiquan exercise has benefit in slowing down bone loss in postmenopausal women. Conference Proceeding of National Conference of the elderly with osteoporosis 2000: 216-217. |
| Qiu YY2008 | Qiu YY, Li QP, Cui Y, Qian M, Guan HY. (2008) Study of Taijiquan exercise on prevention of fall in elder people. Chinese Journal of Gerontology 28: 2055-2056. |
| Ran B2009 | Ran B. (2009) Different exercise intensities on cardiovascular function in the elderly impact analysis. West China Medical Journal 24: 1842-1843. |
| Rand D2011 | Rand D, Miller WC, Yiu J, Eng JJ. (2011) Interventions for addressing low balance confidence in older adults: a systematic review and meta-analysis. Age Ageing 40: 297–306. |
| Reid-Arndt SA2012 | Reid-Arndt SA, Matsuda S, Cox CR. (2012) Tai Chi effects on neuropsychological, emotional, and physical functioning following cancer treatment a pilot study.Complementary Therapies in Clinical Practice 18: 26-30. |
| Ren HY 1992 | Ren HY, Liu JS, Peng LL, Liu YF. (1992) Effects of Tai Ji Quan exercise on brain impedance plethysmogram. Journal of Convalescence & Rehabilitation. 7: 60-61. |
| Rhonda ORR2006 | Orr R, Tsang T, Lam P, Comino E, Singh MF. (2006) Mobility impairment in type 2 diabetes: association with muscle power and effect of Tai Chi intervention. Diabetes Care 29: 2120-2122. |
| Richerson S 2006 | Richerson S, Rosendale KYLE. (2006) Does Tai Chi improve plantar sensory ability A pilot study. Diabetes Technology & Therapeutics 9: 276-286. |
| Robins JLW2013 | Robins JL, McCain NL, Elswick RK Jr, Walter JM, Gray DP, et al. (2013) Psychoneuroimmunology-Based Stress Management during Adjuvant Chemotherapy for Early Breast Cancer. Evid Based Complement Alternat Med 2013: 372908. |
| Romero-Zurita A2012 | Romero-Zurita A, Carbonell-Baeza A, Aparicio VA, Ruiz JR, Tercedor P, et al. (2012) Effectiveness of a tai-chi training and detraining on functional capacity symptomatology and psychological outcomes in women with fibromyalgia. Evidence-Based Complementary and Alternative Medicine 2012: ID614196. |
| Rosado-Perez J2012 | Rosado-Pérez J, Santiago-Osorio E, Ortiz R, Mendoza-Núñez VM. (2012) Tai chi diminishes oxidative stress in Mexican older adults. J Nutr Health Aging 16: 642-646. |
| Ross MC1999 | Ross MC, Bohannon AS, Davis DC, Gurchiek L. (1999) The effects of a short-term exercise program on movement, pain, and mood in the elderly. Results of a pilot study.Journal of Holistic Nursing 17 : 139-147. |
| Sandy C2008 | Burgener SC, Yang Y, Gilbert R, Marsh-Yant S. (2008) The effects of a multimodal intervention on outcomes of persons with early-stage dementia. Am J Alzheimers Dis Other Demen 23: 382-394. |
| Sattin RW2005 | Sattin RW, Easley KA, Wolf SL, Chen Y, Kutner MH. (2005) Reduction in fear of falling through intense tai chi exercise training in older, transitionally frail adults. J Am Geriatr Soc 53: 1168-1178. |
| Serena L2009 | Low S, Ang LW, Goh KS, Chew SK. (2009) A systematic review of the effectiveness of Tai Chi on fall reduction among the elderly. Arch Gerontol Geriatr 48: 325-331. |
| Sha LW 2009 | Sha LW, Gao W. (2009) Experimental study on effect of non- intelligence factor of Taijiquan on taekwondo athletes. Journal of Jilin Institute of Physical Education 25: 138-139. |
| Sha P 2008 | Sha P. Mao HN. (2008) Intervention of Taijiquan exercise and supplement hormone to content of nitrogen monoxide menopause women's blood plasma. Journal of Shaanxi Normal University (Natural Science Edition) 36: 106-108. |
| Sha P2007 | Sha P. (2007) Effect of Taijiquan exercise on the nitrogen monoxidum and blood lipid of elder patients with atherosclerosis. Journal of Clinical Rehabilitative Tissue Engineering Research 11: 6832-6834 |
| Shapira MY 2001 | Shapira MY, Chelouche M, Yanai R, Kaner C, Szold A. (2001) Chi Chuan practice as a tool for rehabilitation of severe head trauma 3 case reports. Arch Phys Med Rehabil 82: 1283-1285. |
| Shen CL2007 | Shen CL, Williams JS, Chyu MC, Paige RL, Stephens AL, et al. (2007) Comparison of the effects of Tai Chi and resistance training on bone metabolism in the elderly: a feasibility study. Am J Chin Med 35: 369-381. |
| Shen CL2007 | Shen CL, Feng D, Esperat MCR, Irons BK, Chyu MC, et al. (2007) Effects of Tai Chi exercise on patients with type 2 diabetes. Integrative Medicine Insights 2:15–23. |
| Shen CL2008 | Shen CL, James CR, Chyu MC, Bixby WR, Brismee JM, et al. (2008) Effects of Tai Chi on gait kinematics, physical function, and pain in elderly with knee osteoarthriti. The American Journal of Chinese Medicine 36: 219–232. |
| Shen CL2009 | Shen CL, Chyu MC, Yeh KJ, Felton CK, Xu KT, et al. (2009) Green tea polyphenols and Tai Chi for bone health: Designing a placebo-controlled randomized trial. BMC Musculoskeletal Disorders 10:110. |
| Shen CL2010 | Shen CL, Chyu MC, Pence BC, Yeh JK, Zhang Y, et al. (2010) Green tea polyphenols supplementation and Tai Chi exercise for postmenopausal osteopenic women: safety and quality of life report. Shenet al. BMC Complementary and Alternative Medicine 10:76. |
| Shen CL2012 | Shen CL, Chyu MC, Yeh JK, Zhang Y, Pence BC, et al. (2012) Effect of green tea and Tai Chi on bone health in postmenopausal osteopenic women: a 6-month randomized placebo-controlled trial. Osteoporos Int 23: 1541-1552. |
| Shen HJ 2012 | Shen HJ, Jing T. (2012) Jujing pill combined taijiquan exercise prescription on seminal plasma biochemical less weak sperm sterility patients and psychological impact study. Chinese Archives of Traditional Chinese Medicine 30: 1685-1689. |
| Shen HJ2012 | Shen HJ, Jing T. (2012) Effect of Taijiquan exercise and Huanshao Capsule on the erectile function and sex hormone levels in patients with psychogenic erectile dysfunction. Journal of Traditional Chinese Medicine 53: 1129-1132. |
| Shen YW 2011 | Shen YW. (2011) A comparative study in patients with type 2 diabetes exercise prescription. Boxing (Physical Education) 3:1-2. |
| Shi L2012 | Shi L. (2012) Effect of Taijiquan exercise on patients with chronic nephritis. Chinese Journal of Physical Medicine and Rehabilitation 34: 467-469. |
| Sjosten N2008 | Sjösten N, Vaapio S, Kivelä SL. (2008) The effects of fall prevention trials on depressive symptoms and fear of falling among the aged: a systematic review. Aging Ment Health 12: 30-46. |
| Song H2008 | Song H, Gao L. (2008) A study on effect of Taijiquan on lumbar disc protrusion. Journal of Beijing Sport University 31: 627-629. |
| Song R2003 | Song R, Lee EO, Lam P, Bae SC. (2003) Effects of tai chi exercise on pain, balance, muscle strength, and perceived difficulties in physical functioning in older women with osteoarthritis: a randomized clinical trial. J Rheumatol 30: 2039-2044. |
| Song R2007 | Song R, Lee EO, Lam P, Bae SC. (2007) Effects of a Sun-style Tai Chi exercise on arthritic symptoms, motivation and the performance of health behaviors in women with osteoarthritis. Taehan Kanho Hakhoe Chi 37: 249-256. |
| Song R2009 | Song R, Roberts BL, Lee EO, Lam P, Bae SC. (2010) A randomized study of the effects of t'ai chi on muscle strength, bone mineral density, and fear of falling in women with osteoarthritis. J Altern Complement Med 16: 227-233. |
| Song R2009 | Song R, Ahn S, So HY, Park IS, Kim HLK, et al.(2009) Effects of Tai Chi exercise on cardiovascular risk factors and quality of life in post-menopausal women. J Korean Acad Nurs 39: 136-144. |
| Song XH 2011 | Song XH. (2011) The scientific connotation of Taijiquan therapy in traditional Chinese medicine. Seek Medical And Ask The Medicine 9: 15-16. |
| Sprod LK2012 | Sprod LK, Janelsins MC, Palesh OG, Carroll JK, Heckler CE, et al. (2012) Health-related quality of life and biomarkers in breast cancer survivors participating in tai chi chuan. J Cancer Surviv 6:146-164. |
| Sui CF 2012 | Sui MF. (2012) TaiJiQuan in treating 36 cases of climacteric syndrome. Western Journal of Traditional Chinese Medicine 25: 84-85. |
| Sun JS 2002 | Sun JS, Meng GC, Wang HB. (2002) The efficacy of Tai chi chuan with spleen-invigorating brain treatment of neurasthenia. J GANSU COLLEGE OF TCM 19:30-31. |
| Sun MJ 2005 | Sun MJ, Yu DD, Lin DM, Lu SD. (2005) Effect of shadow boxing on the physical quality of middle-aged and old women. Chinese Journal of Clincal Rehabilitation 9: 188-189. |
| Sun QQ 2010 | Sun QQ. (2010) Research on the effect of middle一aging and elderly high blood pressure patients quality of life by practice taichi. Dissertation for Master's Degree of Beijing Sport University. |
| Sun W2009 | Sun W. (2009) Effect of tai chi and brisk walking exercise on balance in elderly women. Dissertation for Master's Degree of Shandong Sport University. |
| Sun W2012 | Sun W, Mao DW, Zhang L, Pang F, Zhang XL. (2012) Effect of 16-week Tai Ji Quan practice and brisk walk on respiratory function of elderly women. Chinese Journal of Sports Medicine 31: 669-672. |
| Sun XS 1996 | Sun XS, Wang WW. (1996) Improved simplified types of Taijiquan research on coronary heart disease prevention and control. Shanghai J Prev Med 8:241-242. |
| Sun XS 1998 | Sun XS, Lnsimies Esko. (1998) Simplified taijiquan's influence on the index of heart rate variation, the object of study for the athletics fasten to healthy people to play tai chi chuan again. Chinese Journal of Rehabilitation Medicine 13:225-226. |
| Sun XY 2012 | Sun XY, Tang LL, Zhou LH, Liu LN, Zhou YP, et al. (2012) Effect of comprehensive rehabilitation exercise on the upper limb function rehabilitation and living quality in stage 1 breast cancer patients with postoperative of breast reconstruction . Chin J Phys Med Rehabil 34: 302-305. |
| Sun YX 1988 | Sun YX, Zhou N, Wang XP, Yu XZ, Kui RQ, et al. (1988) The influence of Qi Gong and Taijiquan on lung function in respiratory rehabilitation. Chinese Journal of Rehabilitation 3:168-171. |
| Sun YX1988 | Sun YX, Zhou N, Wang XP, Yu XZ, Yang JY, et al. (1988) The exercise procedures of cardiac rehabilitation and the assessment of the effect on 20 patients. China Journal of Rehabilitation Medicine 3: 2-5. |
| Taggart HM2003 | Taggart HM, A CL, Bae S, Singh K. (2003) Effects of T'ai Chi exercise on fibromyalgia symptoms and health-related quality of life. Orthopaedic Nursing 22: 353-360. |
| Tamim H2009 | Tamim H, Castela ES, Jamnika V, Keirb J, Gracea SL, et al. (2009) Tai Chi workplace program for improving musculoskeletal fitness among female computer users. Work 34: 331-338. |
| Tan L 2004 | Tan L. (2004) Discussion on the Effect of Shadowboxing Exercise on the Cardiovascular Function of Undergraduates. Journal of Shenyang Institute of Physical Education 23:375-376. |
| Tang L 2012 | Tang L, Zeng LQ. (2012) Clinical observation on treating sub-health state in TCM plus Taiji Quan. Clinical Journal of Chinese Medicine 4: 102-103. |
| Tang QH2009 | Tang QH. (2009) Effects of traditional sports on clinical symptom of aged intellectual patients with essential hypertension. Journal of Beijing Sports University 32: 67-69. |
| Taylor D2012 | Taylor D, Hale L, Schluter P, Waters DL, Binns EE, et al. (2012) Effectiveness of tai chi as a community-based falls prevention intervention: a randomized controlled trial. J Am Geriatr Soc 60: 841-848. |
| Taylor-Piliae RE 2006 | Taylor-Piliae RE, Haskell WL, Waters CM, Waters ESF. (2006) Change in perceived psychosocial status following a 12-week Tai Chi exercise programme. Issues and Innovations in Nursing Practice 54: 313–329. |
| Taylor-Piliae RE2004 | Taylor-Piliae RE. (2008) The effectiveness of Tai Chi exercise in improving aerobic capacity: an updated meta-analysis. Med Sport Sci 52: 40-53. |
| Taylor-Piliae RE2006 | Taylor-Piliae RE, Haskell WL ,Froelicher ES. (2006) Hemodynamic responses to a community-based Tai Chi exercise intervention in ethnic Chinese adults with cardiovascular disease risk factors. European Journal of Cardiovascular Nursing 5: 165 – 174. |
| Taylor-Piliae RE2010 | Taylor-Piliae RE, Newell KA, Cherin R, Lee MJ, King AC, et al. (2010) Effects of Tai Chi and Western exercise on physical and cognitive functioning in healthy community-dwelling older adults. J Aging Phys Act 18: 261-279. |
| Taylor-Piliae RE2011 | Taylor-Piliae RE, Coull BM. (2012) Community-based Yang-style Tai Chi is safe and feasible in chronic stroke: a pilot study. Clin Rehabil 26: 121-131. |
| Teng GH 2012 | Teng GH, Chen JF, Liu HY. (2012) The preventive and curative effect analysis of tai chi chuan for lumbar intervertebral disc protrusion. Science and Technology Innovation Herald 9:236. |
| Thomas GN2005 | Thomas GN, Hong AW, Tomlinson B, Lau E, Lam CW et al. (2005) Effects of Tai Chi and resistance training on cardiovascular risk factors in elderly Chinese subjects: a 12-month longitudinal, randomized, controlled intervention study. Clin Endocrinol (Oxf) 63: 663-669. |
| Thornton EW2004 | Thornton EW, Sykes K, Tang WK. (2004) Health benefits of Tai Chi exercise_ improved balance and blood pressure in middle-aged women. Health Promotion International 19: 33-38. |
| Tian GH 2011 | Tian GH. (2011) Clinical observation on 28 cases of treatment of functional dyspepsia. Medical Information 6: 2629. |
| Tian HQ2004 | Tian HQ, Ning HZ, Chen DM. (2004) Taijiquan as adjuvant treatment of senility and middle- age lumber vertebrae diseases. Journal of Hubei Institute for Nationalities (Medical Edition) 21: 24-26. |
| Tian HQ2007 | Tian HQ, Chen DM. (2007) Experimental research on the treatment of Taijiquan exercise prescription as adjuvant therapy to scapulohumeral periarthritis. Chinese Journal of Clinical Medicinal Research 168: 1-2. |
| Tie Y 2008 | Tie Y. (2008) Research on the Impact of the Tai chi Chuan on the Upper Respiratory Tract Infection for Female Students. Health Medicine Research and Practice 5:69-70. |
| Tsai JC2003 | Tsai JC, Wang WH, Chan P, Lin LJ, Wang CH, et al. (2003) The beneficial effects of Tai Chi Chuan on blood pressure and lipid profile and anxiety status in a randomized controlled trial. J Altern Complement Med 9: 747-754. |
| Tsai PF2009 | Tsai PF, Beck C, Jason YC, Kuo YF, Roberson PK, et al. (2009). The effect of tai chi on knee osteoarthritis pain in cognitively impaired elders pilot study.Geriatr Nurs 30: 132–139. |
| Tsai PF2013 | Tsai PF, Chang JY, Beck C, Kuo YF, Keefe FJ. (2013) A pilot cluster-randomized trial of a 20-week Tai Chi program in elders with cognitive impairment and osteoarthritic knee: effects on pain and other health outcomes. J Pain Symptom Manage 45: 660-669. |
| Tsang T2007 | Tsang T, Orr R, Lam P, Comino EJ, Singh MF. (2007) Health benefits of Tai Chi for older patients with type 2 diabetes: The “Move It for Diabetes Study”-A randomized controlled trial. Clinical Interventions in Aging 2:429-439. |
| Tsang T2008 | Tsang T, Orr R, Lam P, Comino E, Singh MF. (2008) Effects of Tai Chi on glucose homeostasis and insulin sensitivity in older adults with type 2 diabetes: a randomised double-blind sham-exercise-controlled trial. Age Ageing 37: 64-71. |
| Tsang TW2009 | Tsang TW, Kohn M, Chow CM, Singh MF. (2009) A randomized controlled trial of Kung Fu training for metabolic health in overweight/obese adolescents: the "martial fitness" study. J Pediatr Endocrinol Metab 22: 595-607. |
| Tsang TW2010 | Tsang TW, Kohn MR, Chow CM, Singh MAF. (2010) Kung fu training improves physical fitness measures in overweightobese adolescents the martial fitness study. Journal of Obesity 2010: ID672751. |
| Tsang WWN2004 | Tsang WWN, Hu-Chan CWY. (2004) Effect of 4- and 8-wk intensive Tai Chi Training on balance control in the elderly. Official Journal of the American College of Sports Medicine 36: 648-657. |
| Tu HL 2005 | Tu HL. (2012) Research on the effects of different exercise on the elderly cardiovascular system. Journal o f Wuhan Institute o f Physical Education 39: 70-73. |
| Tzu TH 2011 | Huang TT, Yang LH, Liu CY. (2011) Reducing the fear of falling among community-dwelling elderly adults through cognitive-behavioural strategies and intense Tai Chi exercise a randomized controlled trial. J Adv Nurs 67: 961-971. |
| Uhlig T2010 | Uhlig T, Fongen C, Steen E, Christie1 A, Ødegård S. (2010) Exploring Tai Chi in rheumatoid arthritis a quantitative and qualitative study. BMC Musculoskeletal Disorders11: 2-7. |
| Venglar M 2005 | Venglar M. (2005) Case report Tai Chi and Parkinsonism. Physiotherapy Research International 10: 116-121. |
| Verhagen VP2004 | Verhagen AP, Immink M, van der Meulen A, Bierma-Zeinstra SM. (2004) The efficacy of Tai Chi Chuan in older adults: a systematic review. Fam Pract 21: 107-113. |
| Voukelatos A2007 | Voukelatos A, Cumming RG, Lord SR, Rissel C. (2007) A randomized, controlled trial of tai chi for the prevention of falls: the Central Sydney tai chi trial. J Am Geriatr Soc 55: 1185-1191. |
| Wall RB2005 | Wall RB, Div M. (2005) Tai Chi and mindfulness-based stress reduction in a Boston Public Middle School. Journal of Pediatric Health Care 19: 230-237. |
| Wang C 2007 | Wang C, Lu W, Wu ZY. (2007) Comparison of the effects between Taiji Quan and Walking excercises on the rehablitation of hypertension on symptoms. Modern Preventive Medicine 34:3535-3536,3543. |
| Wang CC2004 | Wang C, Collet JP, Lau J. (2004) The effect of Tai Chi on health outcomes in patients with chronic conditions: a systematic review. Arch Intern Med 164: 493-501. |
| Wang CC2008 | Wang C, Schmid CH, Hibberd PL, Kalish R, Roubenoff R, et al. (2008) Tai Chi for treating knee osteoarthritis designing a longterm follow up randomized controlled trial. BMC Musculoskelet Disord 9: 108. |
| Wang CC2009 | Wang C, Schmid CH, Hibberd PL, Kalish R, Roubenoff R, et al. (2009) Tai Chi is effective in treating knee osteoarthritis a randomized controlled trial. Arthritis Rheum 61: 1545-1553. |
| Wang CC2010 | Wang CC, Schmid CH, Rones R, Kalish R, Yinh J, et al. (2010) A randomized trial of tai chi for fibromyalgia. N Engl J Med 363: 743-754. |
| Wang CC2010 | Wang C, Bannuru R, Ramel J, Kupelnick B, Scott T, et al. (2010) Tai Chi on psychological well-being: systematic review and meta-analysis. BMC Complement Altern Med 10: 23. |
| Wang CY 2006 | Wang ZY. (2006) The Experimental Study on the Effects of Aerobic Exercises on Health and Mood State of the University Student's of Health Care Class. Dissertation for Master's Degree of Suzhou University. |
| Wang DC 1958 | Wang DC. (1958) Tai Ji Quan for the treatment of tuberculosis. Chinese Journal of Antituberculosis 5: 33-36. |
| Wang DP 2009 | Wang DP, Wang SR, Zhang Y. (2009) Observations of effect of Tai Ji Quan exercise on myofascitis of the back region. Guangming Journal of Chinese Medicine 24: 453-454. |
| Wang DW 2009 | Wang DW, Xu GH. (2009) Study on influence of movement for health preserving in traditional Chinese medicine on type 2 diabetes patients in community. Chinese Nursing Research 23: 2622-2623. |
| Wang JH 2001 | Wang JH, Huang SH. (2001) Effects and mechanism of Tai Chi exercise on hyperlipidemia and diabetes Ⅱ. Journal of Sports and Science. 22: 61-64. |
| Wang JH 2002 | Wang JH, Huang SH, Qiu ZG. (2002) observations of therapeutic effect and discussion on the mechanism of Tai Ji Quan for type 2 diabetes. Chinese Journal of Sports Medicine 21: 357-359. |
| Wang KH 2010 | Wang KH. (2010) A experimental study of effect of Tai Ji Quan on psychology and immune system in undergraduates. Journal of Qiqihar Medical College 31: 773-774. |
| Wang KS 2009 | Wang KS, Zhao CF. (2009) Acupuncture combined with Tai Ji Quan exercise for the treatment of primary hypertension. Guangming Journal of Chinese Medicine 24: 47-48. |
| Wang L 2012 | Wang L, Zhou QA, Xu JY, Yang LJ, Yang B, et al. (2012) Observation of the effect of Taijiquan on rehabilitation of depression after cerebral infarction. Chinese Community Doctors 14: 222. |
| Wang LJ 2001 | Wang LJ, Zhai DP, Qiu C. (2001) Effects of Tai Ji Quan exercise on body balance functions. Modern Rehabilitation 5: 101. |
| Wang LJ 2001 | Wang LJ, Zhai DP, Qiu C. (2001) Effects of Tai Ji Quan exercise on body balance. Modern Rehabilitation 5: 101. |
| Wang LL 2010 | Wang LL. (2010) Taijiquan training on reaction time analysis and research to improve. Dissertation for Master's Degree of Yangzhou University. |
| Wang P 2009 | Wang P, Han QY, Liang RR. (2009) The effect of Taijiquan exercise on health-related quality of life in patients with type 2 diabetes in Community. China Modern Medicine 16: 108-109. |
| Wang P2009 | Wang P, Han XY, Li GT, Liang RR. (2009) Evaluation of varying aerobics interferential effects on type 2 diabetes patients in community. China Medical Herald 6: 34-35. |
| Wang R2013 | Wang R, Liu J, Chen P, Yu D. (2013) Regular tai chi exercise decreases the percentage of type 2 cytokine-producing cells in postsurgical non-small cell lung cancer survivors. Cancer Nurs 36: E27-34. |
| Wang WC2009 | Wang WC, Zhang AL, Rasmussen B, Lin LW, Dunning T, et al. (2009) The effect of Tai Chi on psychosocial well-being: a systematic review of randomized controlled trials. J Acupunct Meridian Stud 2: 171-181. |
| Wang WC2010 | Wang W, Sawada M, Noriyama Y, Arita K, Ota T, et al. (2010) Tai Chi exercise versus rehabilitation for the elderly with cerebral vascular disorder a single-blinded randomized controlled trial. Psychogeriatrics 10: 160-166. |
| Wang WQ 2010 | Li WQ, Huang XF, Bai M. (2010) Impact of Tai Chi exercise on the quality of life of women with osteoporosis in perimenopausal period. Journal of Qilu Nursing 16: 16-17. |
| Wang XC 2009 | Wang XC, Chen SW. (2009) Effect of Yangshi Taijiquan on index of university students’hearts and lungs functions. Journal of Shandong Institute of Physical Education and Sports 25:41-43. |
| Wang XJ 2003 b | Wang XJ.(2003) Effect of Taijiquan on IL-2 of aged peple. Journal of Shangdong Institute of Physical Education and Sports 19: 48-50. |
| Wang XJ 2009 | Wang XJ, Bai YL, Liu QX. (2009) Effects of Tai Ji boxing on negetive mood of teenagers with diabetes mellitus. Journal of Nursing Science. 24: 74-75. |
| Wang XJ 2011 | Wang XJ, Li YJ, Liu NN. (2011) Empirical study of Taijiquan interventions on the prevention and cure of hypertension. Journal of Beijing Sport University 34: 75-77. |
| Wang XJ2003 | Wang XJ.(2003) Influence of Taijiquan on blood viscosity in the elderly. Shandong Sports Science & Technology 25: 34-37. |
| Wang Y 2000 | Wang Y. (2000) About investigation report that Tai Ji Quan can treat chronic disease. Natural Sciences Journal of Harbin Normal University 16: 111-112. |
| Wang Y 2011 | Wang Y. (2011) The regulation of taijiquan exercise on senile hypertension. Chinese Journal of Gerontology 31:4894-4895. |
| Wang YG 2002 | Wang YG, Lv GF, Ren YB. (2002) Research on the efficacy of Tai Ji Quan therapy to type II diabetes mellitus. China Sport Science 22: 72-73. |
| Wang YG 2007 | Wang YG, Zhou WL. (2007) The research of Patients with type 2 diabetes, the IRS - 2 genotype susceptibility differences for sports research. Journal of liaoning normal university (natural science edition) 30: 506-508. |
| Wang YL 2010 | Wang YL, Sun XY, Wang YB, Zhou LH, Fang HX, et al. (2010) Effect of Taijiquan Exercise on the Recovery of Limb Function and the Quality of Life after Surgery of Breast Cancer Patients. China Sport Science and Technology 46:125-128. |
| Wang YL 2012 | Wang YL, Sun XY, Wang YB, Niu F, Liu Y, et al.(2012) Effects of different exercises on the upper limb function and quality of life in patients after breast cancer operation. Chinese Journal of Physical Medicine and Rehabilitation 34: 64-66. |
| Wang YS 1994 | Wang YS, Lin BS, Zhou Y, Han Y, Fu CY. (1994) The study of the plant nerve regulation of Tai Chi exercise. Journal of Beijing University of Traditional Chinese Medicine 17:30-31. |
| Wang YT2004 | Wang YT, Taylor, Pearl M, Chang LS. (2004) Effects of Tai Chi exercise on physical and mental health of college students. The American Journal of Chinese Medicine 32: 453– 459. |
| Wayne PM 2004 | Wayne PM, Krebs DE, Wolf SL, Gill-Body KM, Scarborough DM, et al. (2004) Can Tai Chi improve vestibulopathic postural control? Arch Phys Med Rehabil 85: 142-152. |
| Wayne PM 2007 | Wayne PM, Kiel DP, Krebs DE, Davis RB, Savetsky-German J, et al. (2007) The effects of Tai Chi on bone mineral density in postmenopausal women: a systematic review. Arch Phys Med Rehabil 88: 673-680. |
| Wayne PM2012 | Wayne PM, Kiel DP, Buring JE, Connor EM, Bonato P, et al. (2012) Impact of Tai Chi exercise on multiple fracture-related risk factors in post-menopausal osteopenic women. BMC Complementary&Alternative Medicine 12:7-18. |
| Wayne PM2013 | Wayne PM, Manor B, Novak V, Costa MD, Hausdorff JM, et al. (2013) A systems biology approach to studying Tai Chi, physiological complexity and healthy aging: design and rationale of a pragmatic randomized controlled trial. Contemp Clin Trials 34: 21-34. |
| Wei D 2003 | Wei D, Liu HY. (2003) The simplified Taijiquan exercise has improved heart function in patients with heart failure. Chinese Journal of Clinical Rehabilitation 7: 1460. |
| Wei DY2011 | Wei DY. (2011) Tai ji quan Exercise and Mental Health: a Meta-Analysis. Jour nal of Fujian Normal University ( Natural Science Edition) 27: 111-116. |
| Wei JJ 2006 | Wei JJ. (2006) Effects of Taijiquan and fit running on the cardiopulmonary function of male college students. Dissertation for Master's Degree of Beijing Sport University. |
| Wei Y 2007 | Wei Y, Chen PJ, Tian ML. (2007) Middle-aged and old women before and after 6 months taijiquan exercise cardiopulmonary function change related indicators. Chinese Journal Spots Medicine 26: 604. |
| Wen HX 2010 | Wen HX. (2010) Experimental study of Taijiquan on hyperactive children. Anal Technology 11:45,163. |
| Wolf SL2003 | Wolf SL, Sattin RW, Kutner M, Grady MO, Greenspan AI, et al. (2003) Intense tai chi exercise training and fall occurrences in older, transitionally frail adults a randomized, controlled trial. JAGS 51:1693–1701. |
| Wolf SL1997 | Wolf SL, Barnhart HX, Ellison GL, Coogler CE. (1997) The effect of Tai Chi Quan and computerized balance training on postural stability in older subjects. Atlanta FICSIT Group. Frailty and Injuries: Cooperative Studies on Intervention Techniques. Phys Ther 77: 371-381 |
| Wolf SL2003 | Wolf SL, Barnhart HX, Kutner NG, McNeely E, Coogler C, et al. (2003) Selected as the best paper in the 1990s Reducing frailty and falls in older persons an investigation of tai chi and computerized balance training. J Am Geriatr Soc 51: 1794-1803. |
| Wolf SL2006 | Wolf SL, O'Grady M, Easley KA, Guo Y, Kressig RW, et al. (2006) The influence of intense Tai Chi training on physical performance and hemodynamic outcomes in transitionally frail, older adults. J Gerontol A Biol Sci Med Sci 61: 184-189. |
| Wolfson L1996 | Wolfson L, Whipple R, Derby C, Judge J, King M, et al. (1996) Balance and strength training in older adults: intervention gains and Tai Chi maintenance. J Am Geriatr Soc 44: 498-506. |
| Woo J2007 | Woo J, Hong A, Lau E, Lynn H. (2007) A randomised controlled trial of Tai Chi and resistance exercise on bone health, muscle strength and balance in community-living elderly people. Age Ageing 36: 262-268. |
| Wu F2010 | Wu F, Song EF, Bao Y, Xiang JW, Jia RH. (2010) Tai Chi for the treatment of type 2 diabetes. Chinese Journal of Physical Medicine and Rehabilitation 32: 205-207. |
| Wu G 2006 | Wu G, Keyes LM. (2006) Group tele-exercise for improving balance in elders. Telemedicine and e-Health 12: 561-570. |
| Wu G2010 | Wu G, Keyes L, Callas P, Ren X, Bookchin B. (2010) Comparison of telecommunication, community, and home-based Tai Chi exercise programs on compliance and effectiveness in elders at risk for falls. Arch Phys Med Rehabil 91: 849-856. |
| Wu HC 2008 | Wu HC, Wang FY, Wang ZH. (2008) The influence of 7 days taijiquan exercise on lymphocyte apoptosis. Journal of Yangtze University 5: 119-125. |
| Wu XP 1996 | Wu XP. (1996) Effects of Tai Ji Quan exercise on cardiovascular function in elderly patients with chronic disease. Chinese Journal of Convalescent Medicine. 5: 32-35. |
| Wu YH 2000 | Wu YH. (2000) The investigation of shadow boxing offer old folks body and mind healthy. Journal of Anhui Sports Science 88: 90-92. |
| Xi JP 2004 | Xi JP. (2004) Effect of Taijiquan combined with acupuncture on insomnia. Hebei J TCM 26: 208. |
| Xia Q 2004 | Xia Q, Wang HB, Liu XQ. (2004) A clinical study of prevention of tumble of patients with senile osteoporosis. Chinese Journal of Physical Medicine and Rehabilitation 26: 638. |
| Xiao L 2008 | Xiao L, Zhou Y. (2008) Recovered mechanism of the waist muscle injury by Taijiquan exercise. Journal of Shaanxi Normal University (Natural Science Edition) 36: 101-103. |
| Xiao L 2010 | Xiao L, Zhou Y, Li J. (2010) Effects of fasting blood sugar nitrogen monoxide content and nitric oxide synthase activity in blood serum content in patients with diabetes after intervention of Taijiquan exercise and puerarin. 陕西师范大学学报( 自然科学版) Journal of Shaanxi Normal University (Natural Science Edition) 39: 104-108. |
| Xie W 2012 | Xie W, Wang HQ, Kang SQ, Xie YF, Peng T, Duan GX. (2012) Three kinds of exercise for older people beyond the research on the effects of aging.journal of nursing science 27: 73-75. |
| Xie Y 2008 | Xie Y, Yang L, Zhang BJ, Lan GR. (2008) Rehabilitaion effect of Taijiquan therapy on patients with chronic schizophrenia. J Med Theor & Prac 21: 49-51. |
| Xie YL 2011 | Xie YL, Ren J, Yu DH, Yang HX, Liu J. (2011) The influence of 24 weeks of tai chi exercise on heart rate variability in the elderly. Chinese Journal Spots Medicine 30:842-844. |
| Xie YL 2011 | Xie YL, Chen JY, Zhou ZH, Zhang JJ. (2011) Effect of Taijiquan Exercise on Hyperfipemia in Middle-aged and old People. Chinese Journal of General Practice 9:245-246. |
| Xie YS 2011 | Liang YS, Xie S, Zhang Y. (2011) Observation on the efficacy of taijiquan in treating irritable bowel syndrome. Modern Journal of Integrated Traditional Chinese and Western Medicine 20: 417-418. |
| Xu H 2010 | Xu H, Chen DS. (2010) Wu-style Tai Ji Quan and syndrome of protrusion of lumbar intervertebral disc. New Physical Education-Social Physical Instructor 4: 60-61. |
| Xu SS2012 | Xu SS. (2012) Observation of efficacy of Taijiquan training on rehabilitation of the hepatitis B virus carriers. Chinese Journal of Traditional Medicical Science and Technology 19: 551-552. |
| Yan JH1999 | Yan JH. (1999) Tai chi practice reduces movement force variability for seniors. Journal of Gerontology 54: 629-634. |
| Yan JH2013 | Yan JH, Guo YZ, Yao HM, Pan L. (2013) Effects of Tai Chi in patients with chronic obstructive pulmonary disease: preliminary evidence. PLoS One 8: e61806. |
| Yan JH2013 | Yan JH, Gu WJ, Sun J, Zhang WX, Li BW, et al. (2013) Efficacy of Tai Chi on pain, stiffness and function in patients with osteoarthritis: a meta-analysis. PLoS One 8: e61672. |
| Yan W 20006 | Yan W, Zhang J. (2006) The effect of Taijiquan exercise on the blood glucose and blood lipid of patients with hyperglycemia. Acta Academiae Medicinae Jiangxi 46: 194-195. |
| Yang DH 2011 | Yang DH, Wei Y.(2011) Heat Shock Protein 70 mRNA expression in peripheral white blood cells after Taiji-quan exercise. Journal of Clinical Rehabilitative Tissue Engineering Research 15: 7787-7790. |
| Yang L 2008 | Yang L, Xie Y, Xu ZN, Lu S, Lan GR, et al. (2008) A study on effect of motortherapy on rehabilitation of chronic schizophrenia patients. Chinese Nursing Research 22: 3317-3318. |
| Yang L 2009 | Yang L, Xie Y, Xu ZN, Lu S, Lan GR, et al. (2009) Rehabilitation of patients with chronic schizophrenia by sports therapy. Chinese Journal of Rehabilitation 24: 65-66. |
| Yang ZH 2005 | Yang ZH, Zhou XW. (2005) Chen style tai chi chuan achievement method of the research on the effects of lung function and immune function in the elderly. Journal of Beijing Sport University 28:1212-1223. |
| Yao CD 2010 | Yao CD, Li F, Ma YB. (2010) Effects of Shadow boxing on rehabilitation in patients with chronic heart failure. Chin J Cardiovasc Rehabil Med 19: 364-367. |
| Yao L2013 | Yao L, Giordani BJ, Algase DL, You M, Neil B, et al. (2013) Fall risk-relevant functional mobility outcomes in dementia following dyadic tai chi exercise. West J Nurs Res 35: 281–296. |
| Yao Y 2003 | Yao Y, Yang SD. (2003) Effect of Taijiquan exercise on muscle strength of lower limbs in elderly people. Chinese Journal of Sports Medicine 22: 75-77. |
| Yao YP 2004 | Yao YP. (2004) Effect of tai chi chuan on chronic obstructive pulmonary disease. Chinese Journal of Rehabilitation Theory and Practice 10: 439-440. |
| Ye D 2012 | Ye D. (2012) Research on traditional Chinese medicine syndrome of insomnia in Hongkong and the curative effect of taijiquan. Dissertation for Doctor's Degree of China Academy of Chinese Medical Sciences |
| Yeh GY2004 | Yeh GY, Wood MJ, Lorell BH. (2004) Effects of TaiChi Mind-Body Movement Therapy on Functional Statusand Exercise Capacity in Patients with Chronic Heart Failure: A Randomized Controlled Trial. Acc Current Journal Review 14: 35. |
| Yeh GY2008 | Yeh GY, Wang C, Wayne PM, Phillips RS. (2008) The effect of tai chi exercise on blood pressure: a systematic review. Prev Cardiol 11: 82-89. |
| Yeh GY2009 | Yeh GY, Wang C, Wayne PM, Phillips R. (2009) Tai chi exercise for patients with cardiovascular conditions and risk factors: a systematic review. J Cardiopulm Rehabil Prev 29: 152-160. |
| Yeh GY2010 | Yeh GY, Roberts DH, Wayne PM, Davis RB, Quilty MT, et al. (2010) Tai chi exercise for patients with chronic obstructive pulmonary disease: a pilot study. Respir Care 55: 1475-1482. |
| Yeh GY2011 | Yeh GY, McCarthy EP, Wayne PM, Stevenson LW, Wood MJ, et al. (2011) Tai chi exercise in patients with chronic heart failure a randomized clinical trial. Arch Intern Med 171: 750-757. |
| Yeh GY2013 | Yeh GY, Wood MJ, Wayne PM, Quilty MT, Stevenson LW, et al. (2013) Tai chi in patients with heart failure with preserved ejection fraction. Congest Heart Fail 19: 77-84. |
| Yeh SH 2007 | Yeh SH, Chuang H, Lin LW, Hsiao MY, Wang MW, Yang KD. (2007) Tai chi chuan exercise decreases A1C levels along with increase of regulatory T-cells and decrease of cytotoxic T-cell population in type 2 diabetic patients. Diabetes Care 30: 716-718. |
| Yeh SH2006 | Yeh SH, Chuang H, Lin LW, Hsiao CY, Eng HL. (2006) Regular tai chi chuan exercise enhances functional mobility and CD4CD25 regulatory T cells. British Journal of Sports Medicine 40: 239–243. |
| Yeh SH2009 | Yeh SH, Chuang H, Lin LW, Hsiao CY, Wang PW, et al. (2009) Regular Tai Chi Chuan exercise improves T cell helper function of patients with type 2 diabetes mellitus with an increase in T-bet transcription factor and IL-12 production. British Journal of Sports Medicine 43: 845-50. |
| Yeung A2012 | Yeung A, Lepoutre V, Wayne P, Yeh G, Slipp LE, et al. (2012) Tai Chi treatment for depression in Chinese Americans: a pilot study. Am J Phys Med Rehabil 91: 863-870. |
| Yin H 2007 | Yin H, Yang J. (2007) Rehabilitative effects of Taichi exercise on the subhealthy status of college students. Journal of Clinical Rehabilitative Tissue Engineering Research. 11: 7991-2, 8001. |
| Yu CL 2001 | Yu CL, Xu Y. (2001) Tai Ji Quan and pushing hands for the treatment of scapulohumeral periarthritis. Modern Rehabilitation 5: 99. |
| Yu DH 2011 | Yu DH, Wang S, Yang HX, Ban YS. (2011) The influence of 24 weeks of tai chi exercise on the static balance function of the elderly. Chinese Journal Spots Medicine 30:1115-1118. |
| Yu JF 2005 | Yu JF, Lu HZ, Zhu H, Yu QP, Chen YJ, et al. (2005) Effect analyz of Tai chi chuan in gynecologic malignant cancer clinical application. MODERN NURSING 11:1630-1631. |
| Yu WJ 1997 | Yu WJ, Peng LL. (1997) Clinical observations of Tai Ji Quan for rehabilitation of senile hypertension. Chinese Journal of cardiovascular Rehabilitation Medicine 6: 78-79. |
| Yu Y 2004 | Yu Y.(2004) Effects and mechanism of physical exercise on Type 2 diabetes mellitus associated with hypertension. Journal of Shenyang Insitute of Physical Education 23: 444-445 |
| Yu Y 2006 | Yu Y. (2006) Effect of sports on the body and mind rehabilitation of patients with type 2 diabetes mellitus combining hypertension. Proceedings of 2006 annual conference of China Association for Science and Technology 2006: 63-68. |
| Zeng YH 2009 | Zeng YH, Zhu HX, Liang GY, Gao YP, Ye ZH. (2009) Effect of Taijiquan on equilibrium function and fracture after fall in the elderly. Conference Proceeding of the 10th National Conference of Exercise Therapy 134-137. |
| Zeng YH 2012 | Zeng YH, Zeng YP, Li L, Zhu HX, Liu BL, et al. (2012) Effects of Long-term Tai Ji Exercise on Risk Factors of Cardiovascular Diseases and Incidence of Chronic Disease. Chin J Rehabil Theory Pract 18:1148-1150. |
| Zhan HN 2010 | Zhan HN, Mo Y. (2010) Efficacy observation of Taijiquan as adjuvant treatment on pain of 38 cases with chronic lumber vertebrae diseases. Journal of Guangdong Medical College 28: 657-659. |
| Zhang CH 2012 | Zhang CH, Jiang F, Cai XX, Wang HH, Wang ZL, et al. (2012) Different sports to mitigate the impact of COPD patients with serum cytokines. Progress in Modern Biomedicine 12: 942-943. |
| Zhang DD 2009 | Zhang D. (2009) 60cases of Chinese medicine intervention to occurrence of coronary heart disease angina pectoris. Shaanxi J Tradit Chin Med 30: 824. |
| Zhang DY 2011 | Zhang DY, Pan XC, Zhang YW. (2011) Effect of Chinese medicine combined with Taijiquan on choronic lumbar pain. Journal of China Traditional Chinese Medicine Information 3: 196 |
| Zhang DY 2011 | Zhang DY, Pan XC. (2011) Chinese medicine union Taijiquan treatment chronicity non-bacterial prostatitis curative effect observation. Chinese Manipulation & Rehabilitation Medicine 42: 181. |
| Zhang JG2006 | Zhang JG, Ishikawa-Takata K, Yamazaki H, Morita T, Ohta T. (2006) The effects of Tai Chi Chuan on physiological function and fear of falling in the less robust elderly an intervention study for preventing falls. Arch Gerontol Geriatr 42: 107-116. |
| Zhang JH 2004 | Zhang JH, Yue GF. (2004) Effects of Taijiquan and fit running exercises on blood pressure. Chinese Journal of Clincal Rehabilitation 8: 2957. |
| Zhang L 1994 | Zhang L, Cheng SM. (1994) The influence of fitness exercise prescription for cardiovascular function in the elderly. Shandong Sports Technology 4:18-21. |
| Zhang L 1994 | Zhang L. (1994) The influence of hemodynamic parameters and STI for the elderly PWC 130 before and after test for Tai chi exercise prescription. Chinese Journal of Sports Medicine 13:167-170. |
| Zhang LH2012 | Zhang LH, Wu JJ, Wang ZC.(2012) Effect of 24-form Tai Chi with respiratory rehabilitation training on pulmonary function and quanlity of life of patients with COPD.Acta Universititatis Traditionis Medicalis Pharmacologiaeque Shanghai 26: 53-56. |
| Zhang LJ 2012 | Zhang LJ. (2012) The effect analysis of improvement of carotid atherosclerosis of Taijiquan exercise. Chinese Journal of Rural Medicine and Pharmacy 19: 13-14. |
| Zhang LW 2008 | Zhang LW, Ren SJ, Zhang Y. (2008) Tuina manipulation combined with Tai Ji Quan exercise for the treatment of syndrome of protrusion of lumbar intervertebral disc. China Medical Herald 5: 82-83. |
| Zhang SQ2011 | Zhang SQ, Chen XH. (2011) Effects of Taijiquan exercises on rehabilitation in patients with acute myocardial infarction. Chinese Maniulation & Rehabilitation Medcine 7: 32-33 |
| Zhang TM 2006 | Zhang TM, Tan YM. (2006) Comparison of Yangko and Taijiquan in body building in the middle-aged and elderly women. Chinese Journal of Clinical Rehabilitation 10: 76-78. |
| Zhang W 2007 | Zhang W. (2007)The influence of taijiquan exercise on middle-aged men's sexual function. Chinese Journal of Rehabilitation Medicine 22:57. |
| Zhang WJ 1991 | Zhang WJ, Fu CY. (1991) Tai Chi Exercise on Cardiovascular Function Activities. Journal of Beijing University of Traditional Chinese Medicine 14:46-47. |
| Zhang WJ 2011 | Zhang JW, Yang Y, Tang HL. (2011) The clinical comparison trial of the intervention on the sub-health elders between Tai Chi and others through randomized controlled. Journal of Nanjing Institute of Physical Education (Natural Science) 10: 18-20. |
| Zhang WY 2011 | Zhang WZ. (2011) Observations on the effects of Tai Ji Quan with Caltrate D to Primary Osteoporosis. Dissertation for Master's Degree of Guangzhou University of Chinese Medicine. |
| Zhang XA 2011 | Zhang XA. (2011) The observation to the efficacy of Tai Chi Chuan training in the treatment of generalized anxiety disorder. Medical Journal of Chinese People's Health 23: 937-938. |
| Zhang XH 2009 | Zhang XH. (2009) A review of acupuncture combined with Tai Ji Quan exercise for the treatment of syndrome of protrusion of lumbar intervertebral disc. Guangming Journal of Chinese Medicine 24: 1523-1524. |
| Zhang XL 2007 | Zhang XL. (2010) Effects of Taijiquan and brisk walking exercise on the respiratory function of elderly women. Dissertation for Master's Degree of Shandong Sport University. |
| Zhang Y2008 | Zhang Y, Fu FH. (2008) Effects of 14-week Tai Ji Quan exercise on metabolic control in women with type 2 diabetes. Am J Chin Med 36: 647-654. |
| Zhang YX 2012 | Zhang YX, Zheng ZX. (2012) Brace combined taijiquan exercise therapy of chronic lumbocrural pain. Chinese Journal of Convalescent Medicine 21:904-905. |
| Zhang ZR 2012 | Zhang ZR. (2012) A correlation study of Tai Ji Quan exercise and angiocarpy or lung function in elderly people. Chinese Journal of Primary Medicine and Pharmacy 19: 3087-3088. |
| Zhao CF 2008 | Zhao CF, Ren SJ, Zhang Y. (2008) Observations of effects of acupuncture combined with Tai Ji Quan exercise on syndrome of protrusion of lumbar intervertebral disc. China Medical Herald 5: 86-87. |
| Zhao JG 2000 | Zhao JG, Tian AJ, Chang XY. (2000) The fluence of 42 type shadowboxing on cardiovascular function in elderly intellectuals. Modern Rehabilitation 4:532-533. |
| Zhao LY 1963 | Zhao LY, Zhang YQ. (1963) Observations of effects of Qigong based combination therapy on lung function in patients with tuberculosis. Tianjin Medical Journal 2: 106-109. |
| Zhao SY 2005 | Zhao SY. (2005) Influence of shadowboxing on the personality characteristics of common university students. Chinese Journal of Clinical Rehabilitation 9: 33-35. |
| Zhao XM 2006 | Zhao XM, Wu GS, Li GL. (2006) The influence of body function of simple taijiquan exercise on middle-aged women. Chin J Rehabil Theory Practice 12:167-168. |
| Zhao Y2013 | Zhao Y, Wang Y, Xu XD, Liu YL. (2013) Effectiveness of Tai Chi in Fall Prevention and Balance Function in the Elderly: A Meta-Analysis. Chinese Journal of Evidence-Based Medicine 13: 339-345. |
| Zheng J 2011 | Zheng J, Li J, Song LY, Ni S, Chen YC, et al. (2011) Comprehensive t raditional Chinese medicine intervention for perimenopausal syndrome in women: a community study. Journal of Chinese Integrative Medicine 9:287-291. |
| Zheng JQ 2004 | Zheng JQ. (2004) Observations of effect of Tai Ji Quan on Rehabilitation in patients with senile coronary heart disease. Chinese Journal of Rehabilitation Theory and Practice 10: 429. |
| Zhongn YJ 2009 | Zhongn YJ, Zhong YJ, Liu DM, Zheng SB. (2009) Acute Taijiquan on blood pressure in patients with essential hypertension and its mechanism. Journal Fourth Mil Medicne university 30: 3136-3139. |
| Zhou LP2010 | Zhou LP. (2010) The influence of different exercise modes on ankle proprioception in elderly women . Teach Magazine 11:184-187. |
| Zhou QA 2010 | Zhou QA, Xu JY, Hu AP, Jiang WN, Wang L, et al. (2010) Observation on recover of cerebral infarction patients by Taijiquan training. Chinese Journal of Practicial Nervous Diseases 13: 20-22. |
| Zhou QQ 2011 | Zhou QQ. (2011) Observation on rehabilitation effect of Tai ji Quan therapy for patients with schizophrenia. Nursing and Rehabilitation Journal 10:97-99. |
| Zhou SW 2007 | Zhou SW. (2007) The effect of Taijiquan exercise on the blood pressure and lipid profile in patients with stage 1 essential hypertension. International Medicine & Health Guidance News 13: 60-64. |
| Zhou T 2012 | Zhou T, Qiu ZZ, Liu W. (2012) Correlational research on the effect of Taijiquan exercise on intestinal probiotics and blood lipid of obese old people. Journal of Shandong Institute of Physical Education and Sports 28: 62-66. |
| Zhou Y 2002 | Zhou Y. (2012) Effect of Taijiquan on rehabilition of patients with ankle fractures. Chinese Journal of Sports Medicine 21: 197-199. |
| Zhou Y 2003 | Zhou Y. (2003) Effect of smoking to wash by Chinese medicine cooperation with shadowboxing on convalescence treatment stiff of ankle after fracutures. Shandong Sports Science & Technology 25: 31-33. |
| Zhu JJ 2011 | Zhu JJ. (2011) Different exercises on camparison of the effects of oseteoporosis rearch. Dissertation for Master Degree of Harbin Institute of Physical Education. |
| Zhu XQ 2009 | Zheu XQ, Gan JL, Duan HF, Gao CY, Zhang DW, et al. (2009) Influence of hexagram boxing training on the event-related potential N400 in patients with chronic schizophrenia. Journal of Xinxiang Medical College 26:465-467. |
| Zhu Y 2011 | Li Y, Li JX, Li N, Jin HZ, Hua L, et al. (2011) Effect of Taijiquan on motion control for Parkinson's disease at early stage. Chin J Rehabil Theory Pract 17: 355-358. |
| Zou WL 1995 | Zou WL, Li Z. (1995) Symptoms and brain function in patients with mild cerebral arteriosclerosis. Medical anthology 16: 19-20. |
